# Anti-infective macrozones: design, biological evaluation and structure-activity relationships

**DOI:** 10.5599/admet.3139

**Published:** 2026-01-14

**Authors:** Tomislav Jednačak, Višnja Stepanić, Iva Habinovec, Ivana Mikulandra, Kristina Smokrović, Hana Čipčić Paljetak, Mirjana Bukvić, Jelena Parlov Vuković, Ivan Grgičević, Leda Divjak, Klaus Zangger, Predrag Novak

**Affiliations:** 1 Department of Chemistry, Faculty of Science, University of Zagreb, Horvatovac 102a, HR-10000 Zagreb, Croatia; 2 Ruđer Bošković Institute, Bijenička cesta 54, HR-10000 Zagreb, Croatia; 3 Center for Translational and Clinical Research, School of Medicine, University of Zagreb, Šalata 3, HR-10000 Zagreb, Croatia; 4 Selvita, Prilaz baruna Filipovića 29, HR-10000 Zagreb, Croatia; 5 NMR Centre, Ruđer Bošković Institute, Bijenička cesta 54, HR-10000 Zagreb, Croatia; 6 Labtim Adria d.o.o., Jaruščica 7A, HR-10020 Zagreb, Croatia; 7 Organic and Bioorganic Chemistry, Institute of Chemistry, University of Graz, Heinrichstraße 28 A-8010 Graz, Austria

**Keywords:** Azithromycin conjugates, thiosemicarbazones, synthesis, biological activity, resistance

## Abstract

**Background and purpose:**

To discover novel compounds active against sensitive and resistant bacterial strains, a series of novel azithromycin-thiosemicarbazone conjugates, the macrozones, have been synthesized and their biological activity evaluated with corresponding (quantitative) structure-activity relationship ((Q)SAR) analyses conducted.

**Experimental approach:**

A systematic variation of thiosemicarbazone side-chains and coupling at positions 4"-, 3-, and 9a of the azithromycin scaffold has resulted in a novel class of bacterial ribosome inhibitors.

**Key results:**

Compared to azithromycin, the activity of 4"-macrozones has shown the greatest improvements against efflux-resistant *S. pneumoniae* and *S. aureus*, as well as very good activity of 4" derivatives against *E. faecalis*. QSAR calculations indicate that the antibacterial activity of macrozones is primarily determined by the position of the thiosemicarbazone side chain. Among the conjugated derivatives, the 4"-substituted macrozones exhibit the highest overall activity against a range of sensitive and efflux-resistant Gram-positive bacteria, as well as against Gram-negative *E. coli* strains, while those substituted at 9a- and 3- positions are found to be less potent. The antibacterial activity of macrozones is favourably influenced by larger fractions of their cationic and zwitterionic forms, their capacity for hydrogen bond formation, and the extension of π-electron delocalization involving the thiosemicarbazone moiety.

**Conclusion:**

The results obtained provide a sound basis for guiding further medicinal chemistry efforts toward the discovery of more potent macrolide anti-infectives, with particular emphasis on resistant bacteria that pose a serious threat to human health.

## Introduction

The global rise in bacterial resistance to available antibiotics poses a serious threat to human health worldwide by dramatically compromising our ability to treat infections effectively [1-3]. Antibiotic resistance leads to millions of deaths annually and this number is expected to rise in the future unless new anti-infective agents are developed. Hence, resistance to antibiotics has become a serious threat to human health and to combat resistance, considerable research efforts are focused on discovering novel and safe drugs active against resistant bacterial strains.

Macrolide antibiotics represent a class of bacterial protein synthesis inhibitors that have been used for decades as bacteriostatic or bactericidal agents to treat infections caused by Gram-positive and some Gram-negative bacteria. They bind to 23S rRNA of a 50S bacterial ribosome subunit at or near the peptidyl transferase center (PTC) and block the nascent peptide exit tunnel (NPET), thus disrupting (obstructing) protein biosynthesis. Bound in the NPET macrolides interfere with the ribosome functioning and thus prevent amino acid polymerization process, resulting in the arrest of protein synthesis and bacterial growth [1].

**Scheme 1.**
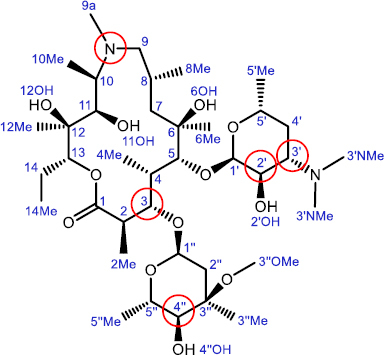
Chemical structure of azithromycin (AZI, **1**) with atom numbering. Substitution sites for macrozone synthesis are shown in red circles

Azithromycin is the first in the class of semi-synthetic 15-membered aza-macrolide antibiotics consisting of a macrolactone ring and two saccharide units attached to positions 3 and 5 (Scheme 1). It was derived from naturally occurring Erythromycin isolated from *Saccharopolyspora erythrea*, by the Beckman rearrangement of the oxime group followed by hydrogenation and methylation [4].

Azithromycin exhibits a broad spectrum of antibacterial activity and possesses an exceptional pharmacokinetic profile, with high metabolic stability, very good cell accumulation and good tolerability. These characteristics contribute to its status as one of the top-selling antibiotics. It is commonly prescribed to treat upper and lower respiratory tract infections and certain sexually transmitted diseases. However, the use of azithromycin and other macrolide antibiotics has declined recently due to the emergence of macrolide resistance. The most prevalent resistance mechanisms are methylation of the ribosomal 23S rRNA catalyzed by the Erm family of methyltransferases (encoded by constitutively or inducibly expressed erm genes and resulting in MLS-resistance), expression of efflux pumps encoded by mef genes, and inactivation of the antibiotic and ribosome protection [1,2].

In recent years, numerous efforts have been made to synthesize novel macrolide compounds by modifying previously successful macrolide scaffolds to enhance their antibacterial and ADMET properties [5-17]. Despite advances in modern biological and chemical methods, the emergence of microbial resistance continues to pose serious challenges to the discovery of better and safer antimicrobial drugs. To address this issue, the strategies that are being developed should include a clear understanding of interactions with biological receptors at the atomic level. Deciphering binding mechanisms and resolving ligand-receptor complexes are essential steps before advancing medicinal chemistry efforts to design and prepare novel inhibitors. X-ray crystallography and NMR spectroscopy have frequently been used to study molecular interactions and their complexes with biological targets and revealed important structural elements responsible for binding [18-28].

We have recently reported that the two-site binding mechanism of interactions of ethyl amino azithromycin derivatives and macrozones, conjugates of azithromycin and thiosemicarbazones, with the *E. coli* ribosome is the most probable mechanism for their activity against some azithromycin-resistant strains [25,26]. This hypothesis has further been explored by the development of a new series of macrozones for which synthesis, antibacterial activities and associated structure-activity relationship (SAR) analysis are reported here.

## Experimental

### In vitro antibacterial activity

The organisms tested represent relevant Gram-positive (*S. pneumoniae*, *S. pyogenes*, *S. aureus* and *E. faecalis*) and Gram-negative (*E. coli*) bacterial pathogens and were either sensitive or resistant to macrolide antibiotics. Macrolide resistance was due to two major mechanisms: the production of efflux pumps (M phenotype) or ribosome modification by methylation (MLSb phenotype). MICs were determined by the broth microdilution method according to CLSI guidelines [29], except that in the medium used to grow *Streptococcus* strains, lysed blood was replaced by 5 % horse serum. The compounds were dissolved in dimethyl sulfoxide (DMSO) at a concentration of 10 mg mL^–1^. Azithromycin and ciprofloxacin were used as controls. Serial twofold dilutions of the test compounds in Mueller-Hinton broth were prepared in 96-well microplates at final concentrations ranging from 0.125 to 64 μg mL^–1^. Bacteria were grown on appropriate agar plates (Becton Dickinson, USA): Columbia agar with 5 % sheep blood for streptococci and Mueller-Hinton agar for staphylococci and *E. coli*. Inocula were prepared by direct colony suspension and microtiter plates were inoculated with approximately 5×10^5^ CFU/mL (5×10^4^ CFU/well). MICs were determined by visual inspection after 18 to 22 hours of incubation at 37 °C in ambient air in J. P. SELECTA Incudigit bacteriological incubator.

### HPLC analyses

The compounds were eluted on an Agilent 1260 Infinity HPLC system with quaternary pump, thermosstatted column compartment, DAD detector and XBridge Phenyl chromatographic column (150×4.6 mm; 3.5 μm) using a mixture of acetonitrile (A) and ammonium buffer solution, pH 10 (B). The mobile phase composition for chromatographic analyses is presented in Table 1. Separations were carried out at 25 °C with 1 mL min^–1^ flow rate, 15 μL injection volume and 210 nm detection wavelength.

**Table 1.** The mobile phase composition for HPLC analyses

*t* / min	*φ*(A) / %
0.0	50
20.0	68
21.0	100
25.0	100
25.1	50
30.0	50

### NMR spectroscopy

NMR experiments were carried out on Bruker Avance III HD 400 MHz and Bruker Avance NEO 600 MHz spectrometers equipped with broadband observed (BBO) Prodigy and inverse TCI Prodigy cryoprobes, respectively, and *z*-gradient accessories. The spectra were recorded in acetonitrile-d_3_, DMSO-d_6_, CDCl_3_ and tris-d_11_ buffer (*c* = 1 mol dm^−3^, pH 7.4) at 298 K using tetramethylsilane (TMS) as an internal standard.

^1^H NMR spectra were measured with 16 to 256 scans, 8.0 to 11.9 kHz spectral width and a digital resolution of 0.24 to 0.36 Hz per point. For DEPTQ spectra, 14336 to 20000 scans were used with a spectral width of 22.1 kHz and a digital resolution of 0.67 Hz.

In COSY experiments, 2048 points in the f2 dimension and 256 increments in the f1 dimension were applied. For each increment, 3 to 18 scans were acquired, with spectral widths of 5600 to 9000 Hz. Digital resolution was 5.47 to 8.88 Hz and 43.76 to 70.33 Hz per point in f2 and f1 dimensions, respectively.

HSQC spectra were recorded with 4096 points in the f2 dimension and 256 increments in the f1 dimension. The number of scans for each increment was 24 to 60 with 6.4 to 9.6 kHz and 18.1 to 27.2 kHz spectral width and a digital resolution of 3.1 to 4.7 and 141.5 to 212.2 Hz per point in the f2 and f1 dimensions, respectively.

For recording HMBC spectra, 4096 points in f2 dimension with a digital resolution of 2.9 to 4.4 Hz per point and 256 increments in the f1 dimension with a digital resolution of 172.9 to 259.4 Hz per point were used. The number of scans per increment was 70 to 100 with 6.0 to 9.1 kHz and 22.1 to 33.2 kHz spectral width in the f2 and f1 dimensions, respectively.

Spectral conditions for NOESY experiments were as follows: 2048 points in the f2 dimension and 1024 increments in the f1 dimension with a digital resolution of 5.9 to 9.4 Hz and 11.7 to 18.7 Hz per point, respectively. For each increment, 24 to 50 scans with a spectral width of 6.0 to 9.6 kHz and a mixing time of 400 ms were acquired.

### Structure-activity relationship analysis

The macrozone dataset is composed of 82 azithromycin derivatives with MIC values determined for 6 bacterial strains: Gram-positive sensitive *S. aureus* 29213, *S. pyogenes* B0542, *S. pneumoniae* B0326, *E. faecalis* 29212 and efflux-resistant *S. aureus* B0331, and a Gram-negative strain *E. coli* 25922 (Tables S1 and S2). The data set also involves the so-called core subset of azithromycin (AZI, **1**) and its aminopropyl-substituted derivatives at the positions 9a (**2**), 4" (**34**) and 3 (**54**), and 14 metal-coordinated compounds containing nickel or copper in the arm. To identify SARs between the MIC activities of macrozones and their molecular features, macrozones are characterized by using several types of descriptors. The structural characteristics of 82 macrozones are represented by the ECFP4 fingerprints [30]. A set of 146 ECFP4 fingerprint bins (out of 1024) present in more than 10 molecules was used for structure similarity analysis. For the subset of 68 macrozones without metal-coordinated compounds, additionally, 374 descriptors are calculated by the software ADMET Predictor™ 12.0 available through the cloud-based SLP University+ program of Simulations Plus Inc., USA and they were used for SAR analyses conducted by machine learning (ML) techniques, PCA and decision trees [31]. The coordination compounds with metal ions are outside the applicability domain of ADMET Predictor models. The so-called Descriptor set of ADMET Predictor includes simple constitutional descriptors (*e.g*. MW, number of bonds N_Bond and electrons N_Electr), topological indices (representing molecular shape through topological distance *i.e*. bond separation matrix, like T_Grav3, TRadb, T_Radc), atom-type electrotopological state (E-state) descriptors, charge-based descriptors, hydrogen bonding and ionization descriptors. A set of charge-based descriptors include partial atomic charge and reactivity sigma and Pi Fukui indices and EEM (electronegativity equalization method), and hardness parameters derived by Simulation Plus (S+) approaches.

Molecular ionization descriptors found significant for antibacterial activity of synthesized macrozones set are FCation, FUnion, FZwitter, QAvgPos and F_HBP, whose meanings are explained in the Results and discussion section. Ionization descriptors are derived from *S* + p*K*_a_ microstate analysis. In addition, physicochemical features like *S* + log*P*, *S* + log*D*, and solubility parameters were included in SAR analyses. However, the synthesized macrozones are similar in their calculated lipophilicity and solubility. They differ considerably according to the solubility factor for salts, SolFactor, as estimated by ADMET Predictor. The latter depends upon ionization and this has been taken into account by the calculated features associated with fractions of ionized (FCation, FZwitter) and unionized (FUnion) species from the ADMET Predictor Descriptor set. Because of this observation, the analyses of physicochemical parameters are not shown or discussed.

ECFP4 fingerprint representations of macrozones and their structural similarity were calculated by R packages rcdk and factoextra, respectively, within RStudio (R version 4.4.1) environment [32]. The multivariate SAR analyses were performed and visualized by the PCA approach available in DataWarrior 6.03 [33]. The PCA results are visualized using score plots and the associated loading plots. For binary classification, the RuleQuest See5 decision-tree approach with 10-fold cross-validation was applied [34]. In machine learning (ML) modeling, the set of macrozones was divided into training and test sets at a 4:1 ratio [35].

## Results and discussion

### Scaffold design

As already mentioned, there are several resistance mechanisms encountered with macrolides and the most frequent are methylation of a specific nucleotide (A2058) of the 50S bacterial ribosomal subunit and expression of efflux pumps. One approach to circumvent resistance includes structural modification of the existing antibiotics. We have previously shown that coupling of azithromycin scaffold and sulfonamides and thioureas yielded conjugates active against both susceptible and macrolide-resistant bacteria [36,37].

Telithromycin, a 14-membered ketolide with an alkyl-aryl side chain at positions 11 and 12 of the macrolactone ring, was essential to restore the activity against macrolide-resistant pathogens. However, it was soon withdrawn from the market due to severe adverse effects, including visual disturbances, hepatotoxicity, syncope and myasthenia gravis. The latter two effects were associated with the involvement of the nicotinic acetylcholine receptor, due to structural homology of nicotine and telithromycin [38]. Consequently, the latest generation 14-membered ketolide solithromycin was not approved by regulators primarily because of its structural similarity with telithromycin and the concern that it might cause similar side effects. Both compounds exert their activity by binding to two different sites at the bacterial ribosome.

Hence, our aim was to develop novel macrolide compounds that maintain the safety profile and favorable pharmacokinetics of azithromycin while targeting multiple ribosomal sites to reduce the risk of bacterial resistance. To achieve that goal, we have coupled thiosemicarbazone moieties to the azithromycin scaffold. Thiosemicarbazones are a class of compounds with a wide spectrum of biological effects. It has been shown that certain thiosemicarbazide conjugates avoid efflux mechanisms in some resistant bacteria and could serve as promising antimicrobial agents [39]. By coupling thiosemicarbazones to azithromycin, we introduced aromatic moiety(ies) and increased lipophilicity, as well as the number of positively charged centers, increasing the alkaline (cationic) properties compared to azithromycin [40]. We hypothesized that systematic alterations to thiosemicarbazone side chains and coupling positions would yield synergistic effects and that novel classes of two-site ribosome-interacting macrolides could be obtained. Our preliminary docking studies of thus formed 15-membered macrozones showed interactions with ribosomal 23S rRNA at two different sites.

### Synthesis

**Figure 1.**
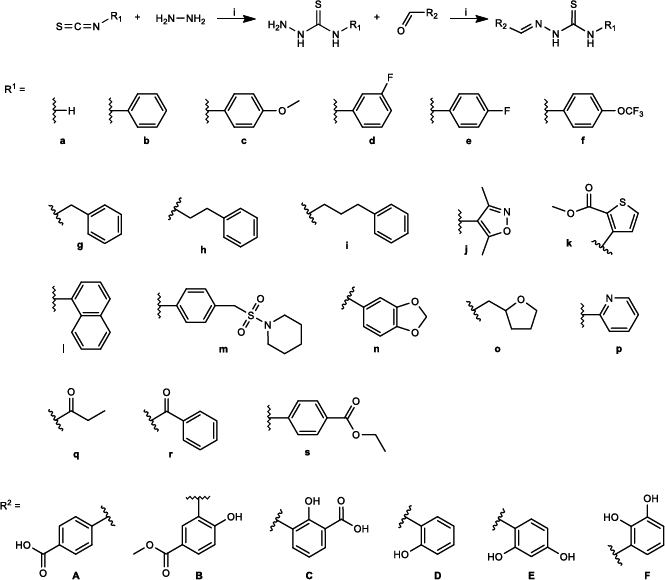
Synthetic route for the preparation of thiosemicarbazones. R^1^ denotes all functional groups on the thiosemicarbazide moiety (labelled with letters **a**–**s**), while R^2^ denotes those of the aldehyde used in the synthesis (labelled with letters **A** to **F**)

**Figure 2.**
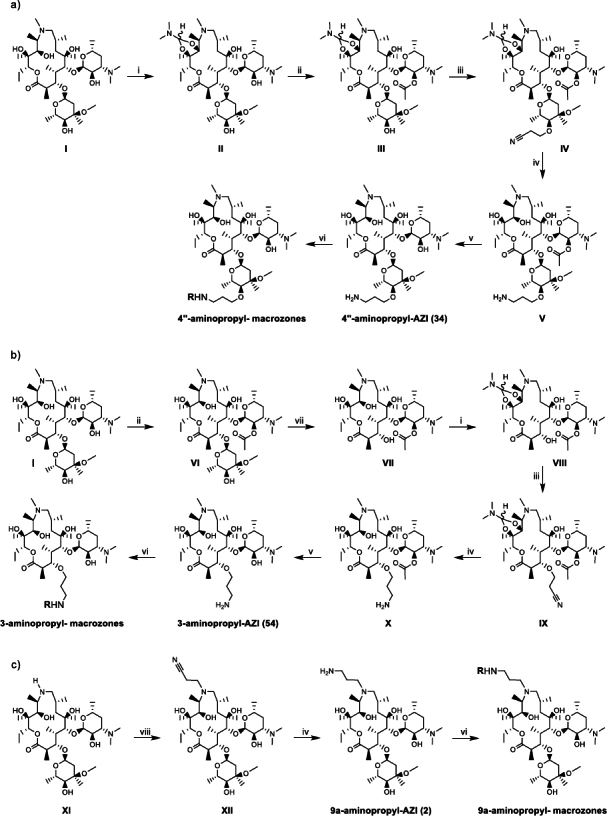
Synthetic route for the preparation of: a) 4"-aminopropyl-, b) 3-aminopropyl- and c) 9a-aminopropyl- macrozones. (i) *N*,*N*-dimethylformamide dimethyl acetal (DMF/DMA) (8 eq), toluene, 60 °C, 24 h; (ii) Ac_2_O, EtOAc, rt, 5 h; (iii) t-BuOH/THF 1/1, acrylonitrile (6 eq), NaH (1.1 eq), -10 °C to r.t., 2 h; (iv) PtO_2_, H_2_, 3.5 bar, AcOH, r.t., 24 h; (v) MeOH, 45 °C, 24 h; (vi) thiosemicarbazone, HATU (1.1 eq), DIPEA (3 eq), DCM, r.t., 24 h; (vii) 6M HCl, 0 °C, 4 h; (viii) BuOH, acrylonitrile, 80 °C, 24 h. The thiosemicarbazone substituents are labeled with R and their structures are shown in Figure 1

**Figure 3.**
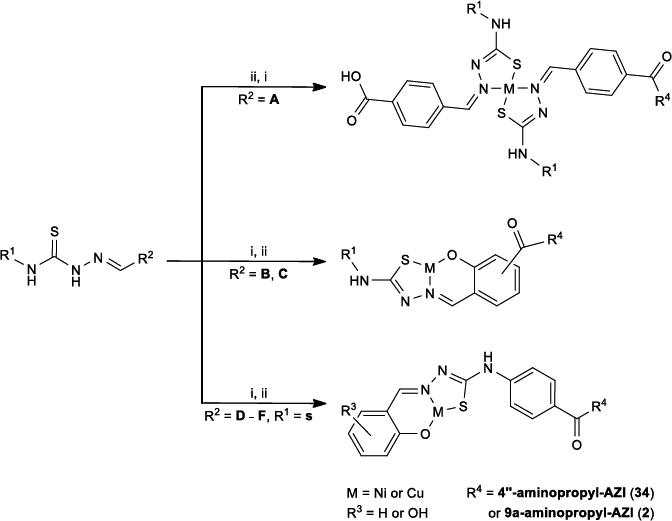
i) 1. HATU (1.2 eq), DIPEA (1.3 eq), DCM, 1 h; 2. 4"-aminopropyl-AZI (**34**) or 9a-aminopropyl-AZI (**2**) (1.1 eq), 2 h; ii) 1. MCl_2_ ·6H_2_O, MeOH, Et_3_N (1.1 eq); 2. HCl (1 mol L^–1^, 2 eq). Chemical structures for R^1^ and R^2^ fragments are shown in Figure 1

The syntheses of novel thiosemicarbazones, macrozones and macrozone metal complexes are depicted in Figures 1 to 3. Their names with corresponding numbers, SMILES, class and antibacterial activities are summarized in the Supplementary material (Tables S1 and S2). Synthetic pathways for preparing 2'- and 3'-aminopropyl- macrozones are given in Figure S1 (Supplementary material). As expected, these compounds did not show antibacterial activity (Table S2), consistent with previous results for macrolides with structural modifications to the desosamine sugar.

Thiosemicarbazones were provided by the addition of aryl/heteroaryl isothiocyanates with hydrazine hydrate followed by the reaction with 4-formyl-benzoic acid. Synthesis of thiosemicarbazones is depicted in Figure 1.

Aminopropyl-azithromycin derivative scaffolds (at the 4", 3 and 9a site, labeled as 4"-aminopropyl-AZI (**34**), 3-aminopropyl-AZI (**54**) and 9a-aminopropyl-AZI (**2**), respectively) were prepared as previously reported [40]. These were used for the preparation of macrozones and their metal complexes.

Multi-step transformations by introducing the acetyl- and cyclic amide acetal- protecting groups at the positions 2'-OH and 11,12-OH, respectively, afforded precursor IV for the regioselective Michael addition with excess of acrylonitrile (Figure 2a). The subsequent reduction of the resulting cyanoethyl adduct IV and deprotection of 2'-OH, 4"-aminopropyl-AZI (**34**) was provided. In a similar manner, by the hydrolysis of the cladinose sugar glycosidic bond, intermediate VII was obtained (Figure 2b).

Furthermore, selective protection of 11-OH, 12-OH and 2'-OH, with acetyl- and cyclic amide acetal- protecting groups, afforded compound VIII. Introduction of the cyanoethyl group at the 3-OH position and final reduction to aminopropyl-chain afforded 3-aminopropyl-AZI (**54**).

Aminopropyl-azithromycin derivative 9a-aminopropyl-AZI (**2**) was obtained in two steps (Figure 2c). The regioselective Michael addition of acrylonitrile at the 9a-*N*-position of the starting intermediate (XI) followed by subsequent catalytic hydrogenation of the 9a-*N*-cyanoethyl group, afforded the 9a-aminopropyl-AZI (**2**) intermediate.

The 4"-aminopropyl-, 3-aminopropyl- and 9a-aminopropyl- macrozones were prepared by the amidation of 4"-aminopropyl-AZI (**34**), 3-aminopropyl-AZI (**54**) and 9a-aminopropyl-AZI (**2**), respectively, with corresponding thiosemicarbazone derivatives (Figures 1 and 2).

We can divide the metal complexes into two groups: those that have two thiosemicarbazone units bound to the metal and those with only one thiosemicarbazone unit and a hydroxyl group at the *ortho*- position relative to the aldehyde. The main difference in the preparation of the group 1 and group 2 macrozone complexes is whether the complex formation between the thiosemicarbazone and metal happens before or after binding to the azithromycin derivative scaffold.

Compounds M4"_NiP12 (**69**), M4"_NiP5 (**70**), M4"_NiP7 (**71**), M4"_NiP16 (**72**) and M4"_NiP6 (**76**) were prepared using the protocol described in ref. [26] with various thiosemicarbazide derivatives shown in Figure 3.

Compounds M4"_NiH6 (**73**), M4"_Ni_4abaR4 (**74**), M4"_Ni3FS6 (**75**), M9_1_Cu (**77**), M9_2_Cu (**78**), M9_3_Cu (**79**), M9_1_Ni (**80**), M9_2_Ni (**81**) and M9_3_Ni (**82**) were prepared by synthesizing the appropriate macrozone [26], which was followed by metal complexation. The complexation was carried out in methanol with 1 equivalent of macrozone and 1.2 equivalents of the MCl_2_ · 6H_2_O (where M is nickel(II) or copper(II)) and several drops of concentrated aqueous ammonia solution. The resulting solution was stirred at 60 °C for 2 h, evaporated under reduced pressure, and suspended in distilled water. The suspension was then extracted with ethyl acetate (3×20 mL). The extract was dried with anhydrous potassium carbonate and evaporated.

### Antibacterial activity

Antibacterial activity of macrozones was tested *in vitro* by the standard broth microdilution method against a panel of Gram-positive and Gram-negative bacteria, including the strains resistant to macrolide antibiotics due to efflux (M phenotype) or ribosomal methylation (cMLSb phenotype) resistance mechanisms. Minimum inhibitory concentrations (MICs) of selected 9a-, 4"- and 3-aminopropyl- macrozones are shown in Table 2 (and Table S2 in Supplementary material), while MICs of selected 4"- and 9a-aminopropyl- macrozone metal complexes are given in Table 3.

**Table 2.** *In vitro* antibacterial activity of selected 9a-, 4"- and 3-aminopropyl- macrozones, represented as minimum inhibitory concentration in μg mL^–1^

Compound	MIC, μg mL^–1^									
	*S. pyogenes* B0542	*S. pneumoniae* B0652	*S. pneumoniae* B0326	*S. pneumoniae* B0633	*S. aureus* ATCC29213	*S. aureus* B0331	*S. aureus* B0330	*E. faecalis* ATCC 29212	*S. cerevisae* ATCC 7752	*E. coli* ATCC 25922
	eryS	eryS	M	cMLS	eryS	M	cMLS						
9_b (**4**)	≤ 0.125		32	> 64	8	> 64	> 64	32	> 64	64
9_c (**5**)	≤ 0.125		16	> 64	8	> 64	> 64	32	> 64	64
9_d (**6**)	≤ 0.125		16	> 64	8	> 64	> 64	32	> 64	32
9_e (**7**)	≤ 0.125		16	> 64	8	> 64	> 64	16	> 64	> 64
9_f (**8**)	4		> 64	64	64	32	32	8	> 64	64
9_h (**10**)	0.5		64	64	8	> 64	> 64	16	> 64	64
9_4 (**22**)	0.5	≤ 0.125	32	> 64	4	> 64	> 64	32	> 64	64
9_13 (**23**)	0.25	≤ 0.125	32	> 64	2	> 64	> 64	32	> 64	32
4"_b (**35**)	0.25		≤ 0.125	> 64	4	4	> 64	1	> 64	64
4"_c (**36**)	≤ 0.125		≤ 0.125	> 64	8	8	> 64	1	> 64	64
4"_e (**38**)	0.5		0.25	> 64	4	4	> 64	1	> 64	32
4"_f (**39**)	4		64	> 64	8	16	16	4	> 64	64
4"_h (**41**)	1		2	32	4	8	> 64	2	> 64	> 64
4"_k (**44**)	0.5		1	> 64	8	16	> 64	4	> 64	64
4"_l (**45**)	0.5		2	64	4	8	> 64	1	> 64	64
4"_9 (**48**)	2	≤ 0.125	8	32	4	16	64	2	64	64
3_a (**55**)	0.25		≤ 0.125	> 64	> 64	> 64	> 64	0.125	2	> 64
3_b (**56**)	1		2	> 64	32	> 64	> 64	8	> 64	> 64
3_c (**57**)	1		1	> 64	16	32	> 64	8	> 64	> 64
3_d (**58**)	1		2	> 64	32	64	> 64	8	> 64	> 64
3_e (**59**)	1		1	> 64	32	> 64	> 64	8	> 64	64
3_1 (**63**)	4	1	16	> 64	8	64	> 64	16	> 64	> 64
3_12 (**67**)	4	2	16	> 64	16	64	> 64	16	> 64	> 64
3_7 (**68**)	1	1	8	64	16	32	32	16	64	64
AZI (**1**)	≤ 0.125	≤ 0.125	8	> 64	1	> 64	> 64	8	> 64	8

**Table 3.** *In vitro* antibacterial activity of selected macrozone metal complexes, represented as minimum inhibitory concentration

Compound	MIC, μg mL^–1^									
	*S. pyogenes* B0542	*S. pneumoniae* B0652	*S. pneumoniae* B0326	*S. pneumoniae* B0633	*S. aureus* ATCC 29213	*S. aureus* B0331	*S. aureus* B0330	*E. faecalis* ATCC 29212	*S. cerevisae* ATCC 7752	*E. coli* ATCC 25922
	eryS	eryS	M	cMLS	eryS	M	cMLS						
M4"_NiP12 (**69**)	64	32	> 64	> 64	> 64	> 64	> 64	64	> 64	> 64
M4"_NiP5 (**70**)	1	ND^a^	> 64	> 64	16	> 64	> 64	16	> 64	64
M4"_NiP7 (**71**)	> 64	> 64	> 64	> 64	> 64	> 64	> 64	16	> 64	> 64
M4"_NiP16 (**72**)	2	1	> 64	> 64	32	> 64	> 64	> 64	> 64	> 64
M4"_NiH6 (**73**)	2	0.5	> 64	> 64	16	> 64	> 64	64	> 64	64
M4"_Ni_4abaR4 (**74**)	0.25	≤ 0.125	32	> 64	8	> 64	> 64	32	> 64	32
M4"_Ni3FS6 (**75**)	> 64	64	> 64	> 64	> 64	> 64	> 64	> 64	> 64	> 64
M4"_NiP6 (**76**)	≤ 0.125	≤ 0.125	16	> 64	2	> 64	> 64	8	> 64	8
M9_1_Cu (**77**)	0.5	0.25	64	> 64	8	> 64	> 64	64	> 64	32
M9_2_Cu (**78**)	0.25	≤ 0.125	32	> 64	8	> 64	> 64	32	> 64	16
M9_3_Cu (**79**)	1	0.25	64	> 64	16	> 64	> 64	64	> 64	32
M9_1_Ni (**80**)	0.5	≤ 0.125	32	> 64	8	32	32	32	> 64	32
M9_2_Ni (**81**)	0.5	≤ 0.125	32	> 64	8	64	64	16	64	> 64
M9_3_Ni (**82**)	0.5	≤ 0.125	32	> 64	16	32	32	32	> 64	32
AZI (**1**)	≤ 0.125	≤ 0.125	8	> 64	1	> 64	> 64	8	> 64	8

^a^not determined.

Macrozones derivatized at the 9a- position, except for 9_f (**8**), exhibit excellent activity against erythronmycin-sensitive streptococci (*S. pneumoniae* and *S. pyogenes*), ranging from ≤ 0.125 to 0.5 μg mL^–1^ and comparable to azithromycin. However, their potential against sensitive *S. aureus* is lower, with MICs in 2 to 8 μg mL^–1^ concentration range. Profiles of 9a-aminopropyl- macrozones against efflux-resistant *S. pneumoniae* and *S. aureus* are comparable to azithromycin or slightly lower. Derivatives 3_a (**55**) to 3_e (**59**), although with clearly improved efficiency against efflux-resistant *S. pneumoniae* (≤ 0.125 to 2 μg mL^–1^) are less active against sensitive strains than their 9a- counterparts. Compared to azithromycin, the activity of 4"-aminopropyl- macrozones shows the greatest improvements not only against *S. pneumoniae* M, but efflux-resistant *S. aureus* as well, with MICs ranging from 4 to 16 μg mL^–1^. Very good activity of 4"- derivatives against *E. faecalis* was also observed. Compounds with the best activity profiles overall are 4"_b (**35**), 4"_c (**36**) and 4"_e (**38**).

Although complexes of 9a-aminopropyl- macrozones maintain excellent activity on erythromycin-sensitive streptococci, macrozone complexes are, in general, less potent, and none of them had good activity against macrolide-resistant strains.

### Structure-activity relationship analysis

**Figure 4.**
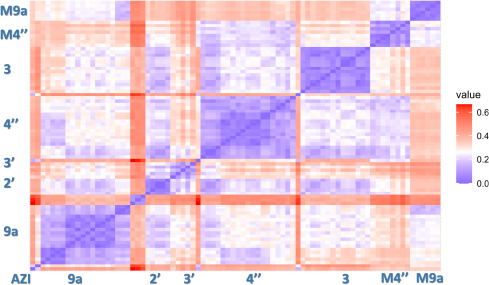
Heat map representation of structural diversity of synthesized macrozones ordered as in Table S1. The similarity measure is Jaccard index (Jaccard = 1-Tanimoto). A deeper blue color indicates a higher similarity between the compound pair

The SAR analysis was initialized by the characterization of the chemical space of 78 synthesized azithromycin conjugates with thiosemicarbazones and four cores: AZI (**1**), 9a-aminopropyl-AZI (**2**), 4"-aminopropyl-AZI (**34**) and 3-aminopropyl-AZI (**54**) in terms of their structures and molecular properties (Table S1). The diversity of macrozone structures is estimated from their representation in ECFP4 fingerprints, using the Tanimoto coefficient as the similarity measure. As illustrated by the heat map in Figure 4, the novel macrozone conjugates mainly differ by coupling position, while thiosemicarbazone side-chains are shared among 9a, 3, 2', 3' and 4" sub-sets.

In addition, the variability of non-coordinated macrozones (Table S1, compounds **1** to **68**) was described using a PCA approach, employing 374 and around 100 molecular features from the Descriptor and Physicochemical subsets, respectively, calculated with the ADMET Predictor software [31]. From the PCA analysis, 138 features with the highest contributions to the first three principal components (PCs) were selected for subsequent SAR analyses on bacterial strains with both active and inactive macrozones.

The 68 macrozones mainly differ from each other in several molecular features, including molecular size represented by MW, N_Bond, N_Electr, the thiosemicarbazone side-chain/arm length (SHCH_321 - atom-type hydrogen E-state index for -CH_3_, -CH_2_- and >CH- groups (saturated aliphatic carbon)), topological shape-related indices T_Grav3 and T_HydroR, molecular polarizability PolarizM, the electrophilicity index Elephity and equalized molecular hardness EqualEta. T_Grav3 denotes the 3rd-order gravitational index of heavy atoms in a molecular graph, which quantifies the distribution of atomic masses within the molecular structure. It decreases in the order 4" > 9a > 3 substituted macrozones and higher values of T_Grav3 usually correspond to more complex (substituted) or branched structures. T_HydroR refers to the distribution of hydrophobic regions across the molecule, considering its topology. Elephity is an electrophilicity index measuring a molecule’s ability to accept electrons, *e.g*. through noncovalent interactions like hydrogen bonding, nucleophilic attack, or polar interactions. PolarizM and EqualEta estimate the resistance of a molecule’s electronic structure towards external electric field and charge transfer, respectively. Such a set of synthesized and experimentally tested macrozones, diverse enough in features influencing intermolecular interactions, enables us to reveal SARs.

**Figure 5.**
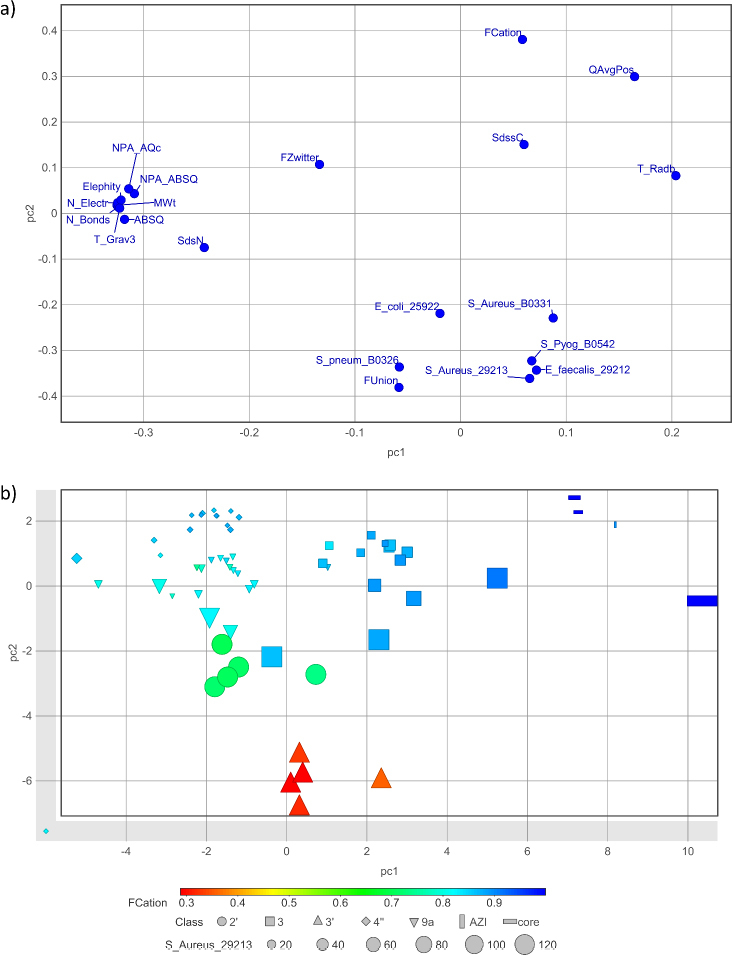
The loading (a) and score (b) plots of PCA performed in terms of MIC values and the most important molecular descriptors for the set of 60 macrozones and **AZI** and 3 additional cores. Compounds are colored according to estimated fraction of cationic species FCation at pH = 7.4. Marker shape/size is determined by the substitution place/MIC value against *S. aureus* 29213. The PC1 and PC2 describe cumulatively 69.8 % of variance

Carrying out PCA using the same set of 138 calculated molecular features and the measured MIC values for the six tested bacterial strains (5 Gram-positive strains and Gram-negative *E. coli*) revealed descriptors significant for the antibacterial activity of the synthesized macrozones (Figure 5a). In addition to molecular size, molecular features associated with the ionization state and the atomic charge are found to contribute to the antibacterial activity of macrozones. Increase in fractions of cationic (FCation) and zwitterionic (FZwitter) forms and a decrease of neutral (FUnion) species reduce MIC values, *i.e*. increase antibacterial activity. FCation and FUnion are estimated cumulative contributions of purely cationic species and all species with zero net charge, respectively, to the fraction ionized at pH 7.4. FZwitter is a portion of FUnion contributed by zwitterionic species and is independent of pH.

In accordance, the descriptor QAvgPos (calculated as the population average across all ionized species of the net positive formal charge at pH 7.4) as well as ABSQ (sum of absolute values of PEOE partial atomic charges) and NPA_ABSQ and NPA_AQc (sums of absolute values of partial atomic charges on all atoms and on C atoms only, respectively, estimated by Natural Population Analysis (NPA)) were also found to be important. Ionization, charge localization as well as electron distribution and polarization as defined by these calculated parameters, suggest that electrostatic interactions, hydrogen bonding, and charge distribution are key determinants of antibacterial behavior of macrozones and fine-tuning them can give control over their bioactivity. However, most of the explained variance is associated with different coupling positions (Figure 5b) and may be due to their distinct interactions with negatively charged regions of ribosomal RNA and with bacterial membrane components. Atom-type E-State index for =N– groups (SdsN) differentiates cores from macrozones, while atom-type E-state index for =C< groups (SdssC) describes differences at the end of the thiosemicarbazone substituent, which is an important parameter for differentiating substituents within each of the macrozone series defined by the substituting place. The topological index related to molecular radial distribution T_Radb is the largest for AZI (**1**), the cores **2** and **34** and macrozones from the subgroup 3 lacking the cladinose sugar, as illustrated by the association of variables and macrozones positioned in analogous regions of the loading and score plots in Figure 5. Increase of Elephity also strengthens antibacterial activity. Elephity estimates a molecule’s ability to accept electrons and the stabilization energy of a system when it acquires additional electron density from a nucleophile, such as mRNA atoms at the target ribosome site. The same molecular features were found to be the most important (10 % of 138 variables determined by their contributions to PC1, PC2 and PC3 according to the percentage of variance described by the corresponding PC) by performing PCA including MIC values for each bacterial strain separately.

**Figure 6.**
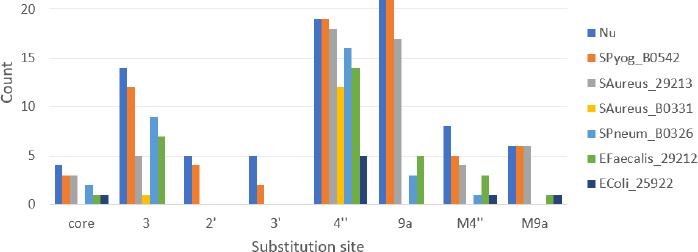
Total number (Nu) and numbers of active macrozones against six bacterial strains shown in different colors. The substitution site is the key feature determining antibacterial activity of macrozone conjugates

The shape of the molecule markers in the score plot in Figure 5b immediately highlights the importance of the substitution site for antibacterial activity. In addition to AZI and its derivatives with an aminopropyl chain at positions 4" and 9a, macrozones substituted with thiosemicarbazones at positions 4", 3, and 9a are also active against each of the tested sensitive Gram-positive strains, although to varying extents. **AZI** with the positively charged 3-aminopropyl chain and all its derivatives with thiosemicarbazone substitutions at positions 2' and 3' are generally inactive. In Figure 6, it is illustrated the importance of the substitution site on the macrolactone core for the assumed two-site bacterial ribosome targeting.

The macrozones are active against all tested Gram-positive bacteria with the number of active molecules with MIC values equal or less than 16 μg mL^-1^ decreasing in the order *S. pyogenes* B0542 > *S. aureus* 29213 > *S. pneumoniae* B0326 = *E. faecalis* 29212 > *S. aureus* B0331 (Figure 6). To find which molecular features are important for activity on each of the bacterial strains, we performed ML modeling by using a decision tree classification algorithm and 138 selected molecular features. The classification decision tree models were built for the subset of 54 3-, 4"- and 9a- substituted macrozones for the strains for which enough active and inactive molecules were determined. The PC1/PC2/PC3 defined in terms of these 138 descriptors explains 34.5 %/13.5 %/12.0 % of variance among 54 synthesized macrozones.

The aim was to find some rules governing the antibacterial activity of macrozones. The macrozones were divided into two classes. The class of actives contains macrozones with MIC values equal or less than 16 μg mL^–1^, while the class of inactive includes the rest of the compounds. The binary classification models were built by the decision tree approach using the See5 algorithm. Interestingly, no general rules relating MIC values with features of substituents applicable for all substitution places that are 3, 4" and 9a series are found. Only a few rules are observed, and they were found for subsets determined by the substitution site in spite of some similarity among substituents at different positions of the AZI core (Figure 4). The rules are also different for different bacterial strains. These findings suggest that macrozones substituted at different positions on the AZI core may engage distinct binding interactions with the bacterial ribosome and that their uptake into bacterial cells could be influenced by the site of substitution. The observed variability between bacterial strains further indicates that both target binding and bacterial permeability may be sensitive to structural modifications at specific positions.

**Figure 7.**
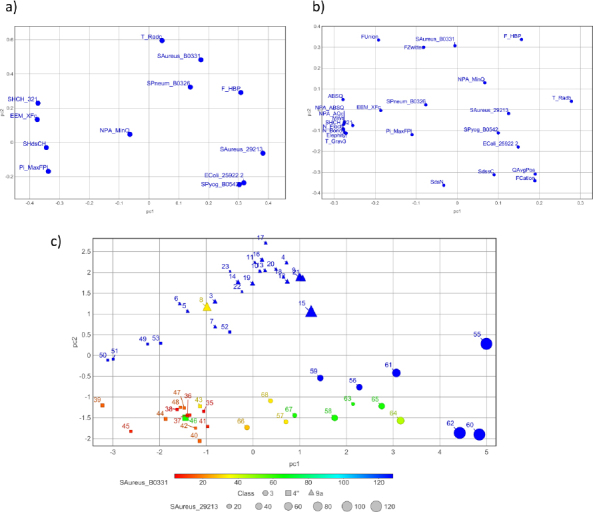
The loading PCA plots for 54 3-, 4"- and 9a substituted macrozone conjugates and 5 bacterial strains in terms of descriptors identified by (a) decision trees (b) PCA and decision trees, to be important for differentiating active (MIC ≤ 16 μg mL^–1^) and inactive molecules. The first two PCs explain 49.9 % and 66.6 % of variance, respectively. (c) The score plot associated with the loading plot (a)

For the sensitive strain *S. aureus* 29213, all 4"- macrozones (except molecule **46** with a sulfonyl group) and most of 9a-substituted macrozones are active, *i.e*. have MIC < 32 μg mL^–1^. In contrast, most of the 3-substitued conjugates lacking the cladinose sugar are inactive (Figures 6 and 7c). The reactivity descriptor EEM_XFc (Maximum sigma Fukui index on C-carbon) was the most discriminating between inactive and active compounds within series 3. The classification rule found is EEM_XFc ≤ 3.019 for inactive compounds. EEM_XFc presents sigma atomic charges calculated by the EEM method through solving equations that balance electronegativity differences between atoms. EEM is based on the principle of electronegativity equalization, which states that, in equilibrium, the electronegativity of all atoms in a molecule adjusts to balance charge distribution. For example, the presence of polar hydroxyl or ether O-atoms at the outer end of thiosemicarbazone arm increases EEM_XFc value, *i.e*. decreases MIC values, implying possible importance of *e.g*. polar / hydrogen bond interactions with ribosome of the side-chain at 3- site of the macrolactone ring.

For the efflux-resistant strain *S. aureus* B0331, 9a-substitued macrozones are also inactive (Figure 6). This finding may point to the cladinose 4"-OH group as an important interaction site for the uptake and accumulation of macrozones in *S. aureus*. The parameter F_HBP, which corresponds to a population average across all ionized species (pH = 7.4) of the number of hydrogens (protons) available for hydrogen bonding divided by the number of heavy atoms (F_HBP > 0.108: inactive), is found to be the most important for differentiating inactive from active macrozones (Figure 7). The macrozones with fewer hydrogen bond donors are more active. Additionally, for the 4"-substituted conjugate to be active, its Atom-type hydrogen E-state index for -CH_3_, -CH_2_- and >CH- groups (*i.e*. number of saturated aliphatic carbon in the substituent) SHCH_321 should be ≤ 42.107, pointing to the significance of the length of the thiosemicarbazone arm and position of its end for the activity on the strain *S. aureus* B0331.

In contrast, for the efflux-resistant strain *S. pneumoniae* B0326, 3-substituted conjugates and 9a-modified derivatives are also active, with the former being more efficient than the latter. For 3- series, no rules are found in terms of the employed descriptors. In the 9a- series, derivatives with Maximum scaled Pi Fukui+ index (nucleophilic, relates to π electron density for the lowest unoccupied molecular orbital (LUMO)) Pi_MaxFPI > 4.2, corresponding to more extended π-electron delocalization, are active molecules. The 4" derivatives are active if their topological equivalent of the smallest ellipsoidal radius of the molecule is greater T_Radc > 4.914. It is interesting to note that T_Radc values for 4"-derivatives are somewhat smaller than those for 3- and 9a- substituted macrozones. T_Radc is a topological equivalent of the smallest ellipsoidal radius of the molecule, indicating the importance of the more elongated or less branched shape at the arm end for the *S. pneumoniae* B0326 ribosome targeting.

For *E. faecalis* 29212 no simple rules were identified using decision tree approaches and 138 selected descriptors. Although, in general, macrozones with lower F_HBP values (indicating less potential for hydrogen bond activity) are more likely to exhibit activity against *E. faecalis* [41], similar to the trend observed for *S. aureus* strains (Figure 7).

In addition to AZI (**1**), the five non-coordinated 4" macrozones **49** to **53** are active on a Gram-negative strain *E. coli* 25922 with MIC < 32 μg mL^–1^ (Table S2). The highly cationic 9a- and 4"-aminopropyl-substituted cores **2** and **34** as well as 9a-coupled conjugates **6** and **17** and 4"-conjugate **38** are active on a Gram-negative strain *E. coli* 25922 with MIC = 32 μg mL^–1^. The 4"-macrozones **49** to **53** all have the feature Atom-type hydrogen E-state index for =CH– groups SHdsCH > 1.667, which is associated with the presence of *ortho-* phenoxy group. The compounds **6** (9a) and **38** (4") contain an F-atom in *meta-* position to thiosemicarbazone. All these molecules are characterized by relatively larger values of calculated molecular descriptors HBDch, Pi_MaxFPI or Pi_FMi4 as compared to the rest of the synthesized molecules. The parameter HBDch corresponds to a sum of the NPA partial atomic charges on the hydrogen-bond donor hydrogen atoms. Pi_FMi4 (Fourth component of the autocorrelation vector of scaled Pi Fukui- indices (electrophilic)) is connected with the shape of the π-electron system. For macrozones, the values of these parameters are influenced by the extension of π-electron delocalization/π-conjugated system, which is greater in macrozones with *ortho-* OH or *meta-* F phenyl groups bound to the thiosemicarbazone moiety. The phenoxy group may also enable deprotonation of the conjugated thiosemicarbazone N-atom and formation of zwitterionic species at pH 7.4. However, 9a-derivatives with a phenoxy group and the potential to exist in a zwitterionic state are not active against *E. coli*. This finding may indicate different binding sites for the thiosemicarbazone arm coupled at different positions at the AZI core, assuming the same binding site for the AZI core in all macrozones.

## Conclusions

It has been demonstrated that the antibacterial activity of macrozones, novel azithromycin-thiosemicarbazone conjugates, against various bacterial strains is primarily governed by the coupling position of the thiosemicarbazone side chain. The 4"-substituted macrozones have exhibited the highest activity against a range of sensitive and efflux-resistant Gram-positive bacteria, as well as against Gram-negative *E. coli* strains. Macrozones conjugated at positions 9a and 3 have also shown antibacterial effect against the tested Gram-positive strains, although with varying degrees of potency, while the 2'- and 3'-substituted conjugates have generally been inactive.

The (Q)SAR analysis has indicated that the thiosemicarbazone side chain may interact with distinct binding sites on the bacterial ribosome depending on the coupling position and pointed out differences in molecular properties. Capacity for hydrogen bond formation and the extension of π-electron delocalization involving the thiosemicarbazone moiety have been found to be important for their antibacterial activity. Ionization, charge localization, electron distribution, and polarization, as estimated by the employed 2D descriptors, provide valuable guidance for further derivatization of the most promising 4"-macrozone series. Fine-tuning electrostatic interactions, hydrogen bonding capacity and electron distribution in the thiosemicarbazone side chain can be crucial for optimizing their two-site binding to bacterial ribosomes and also enhancing bacterial cell uptake.

## Supplementary material

Additional data are available at https://pub.iapchem.org/ojs/index.php/admet/article/view/3139, or from the corresponding author on request.


