# Supplementary material for: Anti-infective macrozones: design, biological evaluation and structure-activity relationships
Source: ADMET DMPK. 2026 Jan 14;14:3139. doi: 10.5599/admet.3139 (PMC12994594; doi:10.5599/admet.3139)
Supplement: Supplementary file 1 [file ADMET-14-3139-S1.pdf]

ADMET & DMPK **14** *(2026) S3139*

Open Access : ISSN : 1848-7718

https://www.pub.iapchem.org/ojs/index.php/admet

Supplementary material to

Anti-infective macrozones: design, biological evaluation and structure-activity relationships

Tomislav Jednačak[1], Višnja Stepanić[2,*], Iva Habinovec[1], Ivana Mikulandra[1], Kristina Smokrović[1], Hana Čipčić Paljetak[3], Mirjana Bukvić[4], Jelena Parlov Vuković[5], Ivan Grgičević[6], Leda Divjak[1], Klaus Zangger[7] and Predrag Novak[1,*]

[1]*Department of Chemistry, Faculty of Science, University of Zagreb, Horvatovac 102a, HR-10000 Zagreb, Croatia*
[2]*Ruđer Bošković Institute, Bijenička cesta 54, HR-10000 Zagreb, Croatia*
[3]*Center for Translational and Clinical Research, School of Medicine, University of Zagreb, Šalata 3, HR-10000 Zagreb, Croatia*
[4]*Selvita, Prilaz baruna Filipovića 29, HR-10000 Zagreb, Croatia*
[5]*NMR Centre, Ruđer Bošković Institute, Bijenička cesta 54, HR-10000 Zagreb, Croatia*
[6]*Labtim Adria d.o.o., Jaruščica 7A, HR-10020 Zagreb, Croatia*
[7]*Organic and Bioorganic Chemistry, Institute of Chemistry, University of Graz, Heinrichstraße 28 A-8010 Graz, Austria*

ADMET & DMPK **00(0)** (2025) 3128; https://doi.org/10.5599/admet.3139

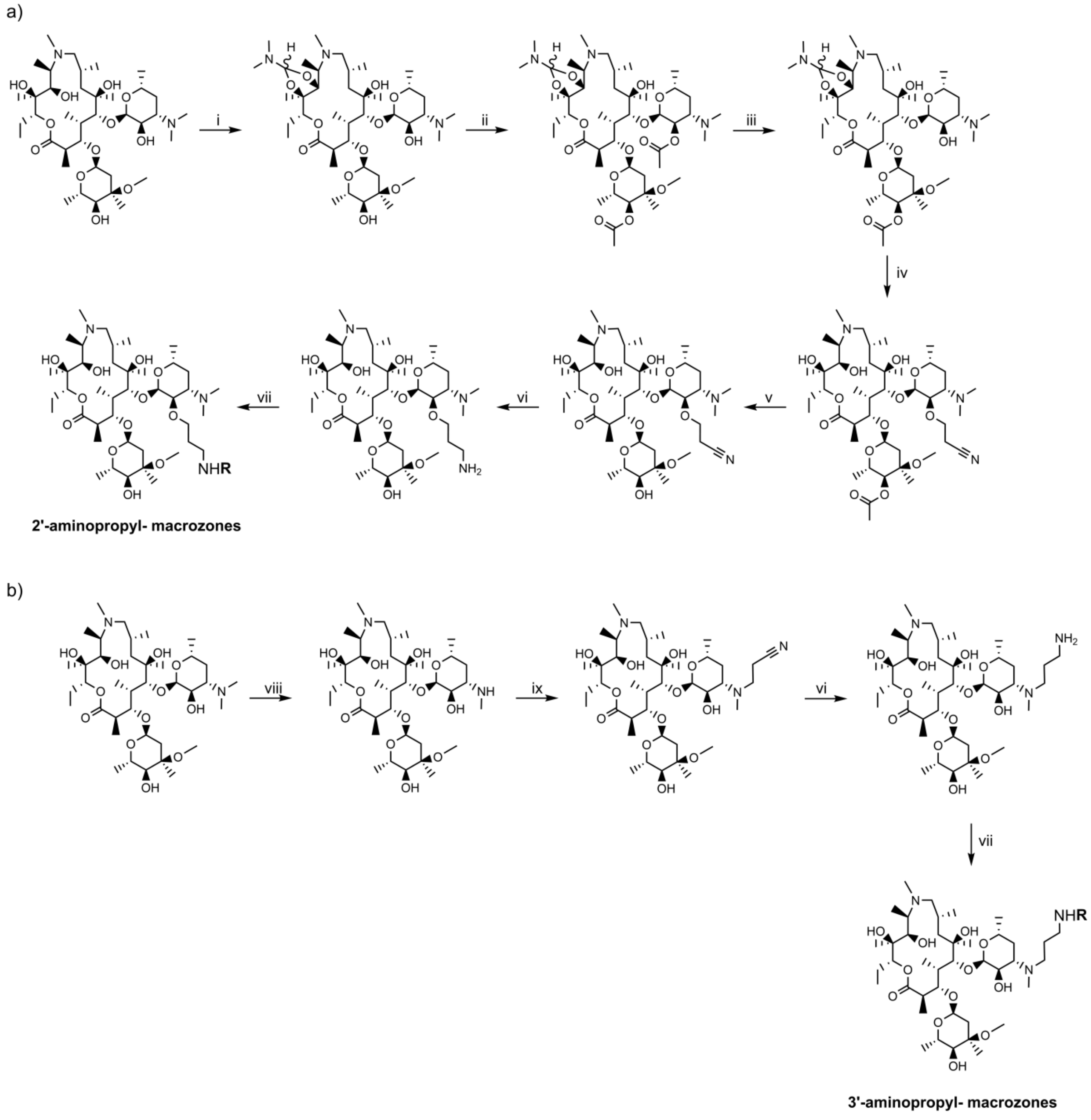

Figure S1. Synthetic route for the preparation of: a) 2'-aminopropyl- and b) 3'-aminopropyl- macrozones. (i) *N*,*N*-dimethylformamide dimethyl acetal (DMF/DMA) (8 eq), toluene, 60 °C, 24 h; (ii) Ac_2O, EtOAc, rt, 5 h; (iii) MeOH, 45 °C, 24 h; (iv) t-BuOH/THF 1/1, acrylonitrile (6 eq), NaH (1.1 eq), –10 °C to r.t., 2 h; (v) 6M HCl, 0 °C, 4 h; (vi) PtO_2/C, H_2, 3.5 bar, glacial AcOH, r.t., 24 h; (vii) thiosemicarbazone, HATU (1.1 eq), DIPEA (3 eq), DCM, r.t., 24 h; (viii) DEAD (1 eq), acetone, r.t., 24 h; (ix) acrylonitrile in excess, 60 °C, 24 h. The thiosemicarbazone substituents are labeled with R and their structures are shown in Figure 1

A

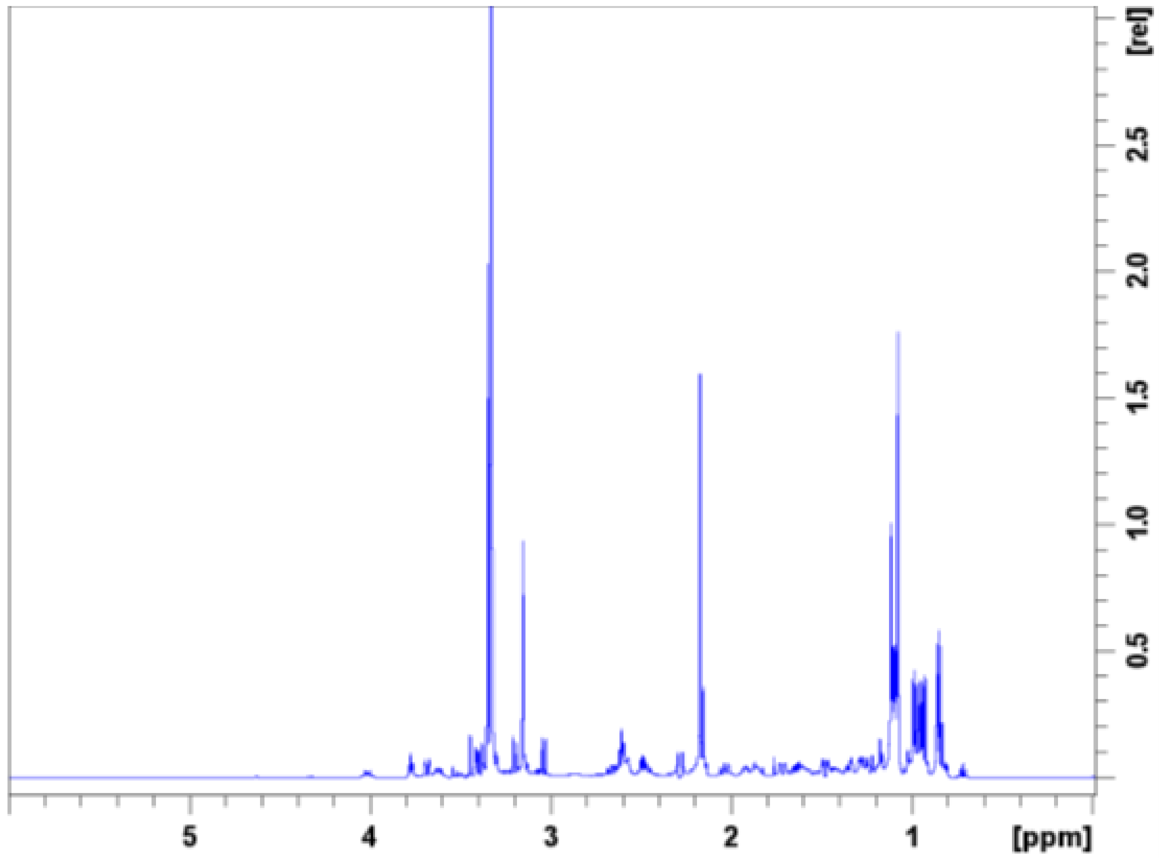

B

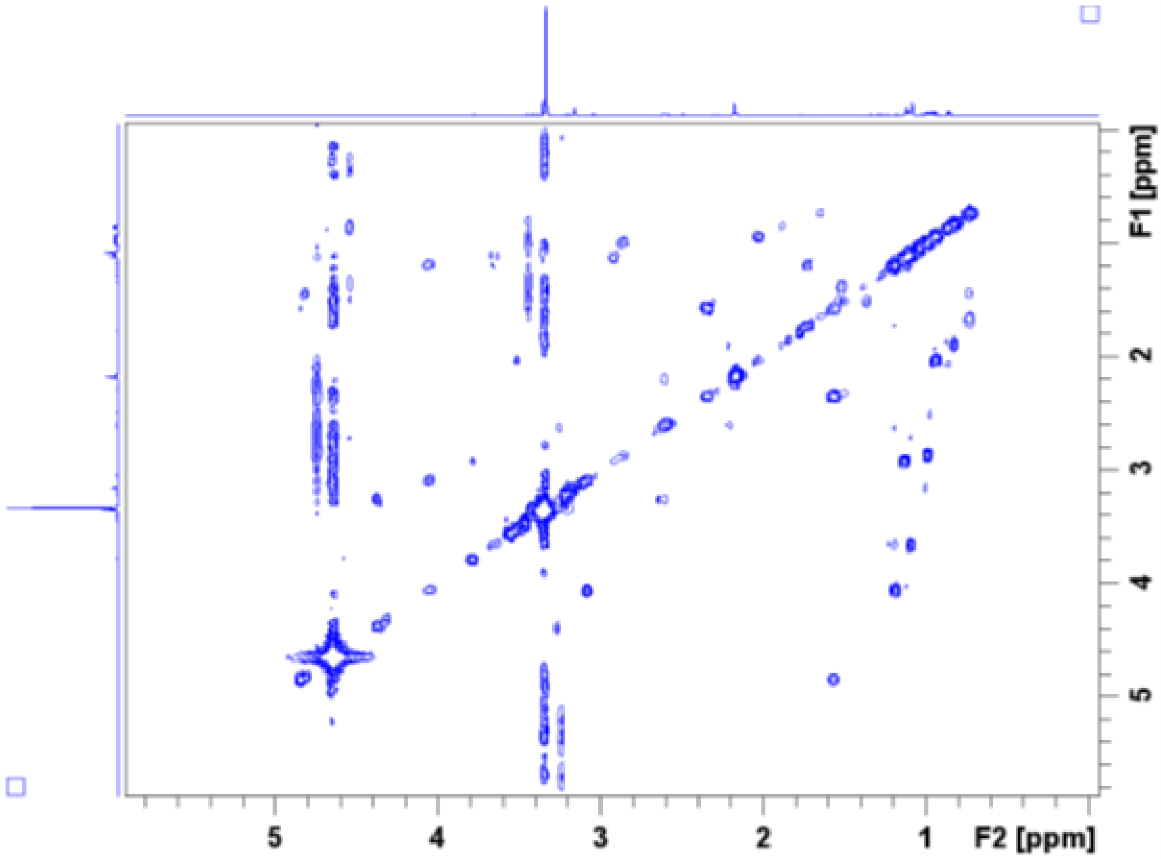

Figure S2. A) ^{1}H and B) COSY NMRspectra of 9a-aminopropyl-AZI (**2**) recorded in tris-d_{11} buffer (c = 1 mol dm^{-3}, pH 7.4) at 600 MHz and 298 K

A

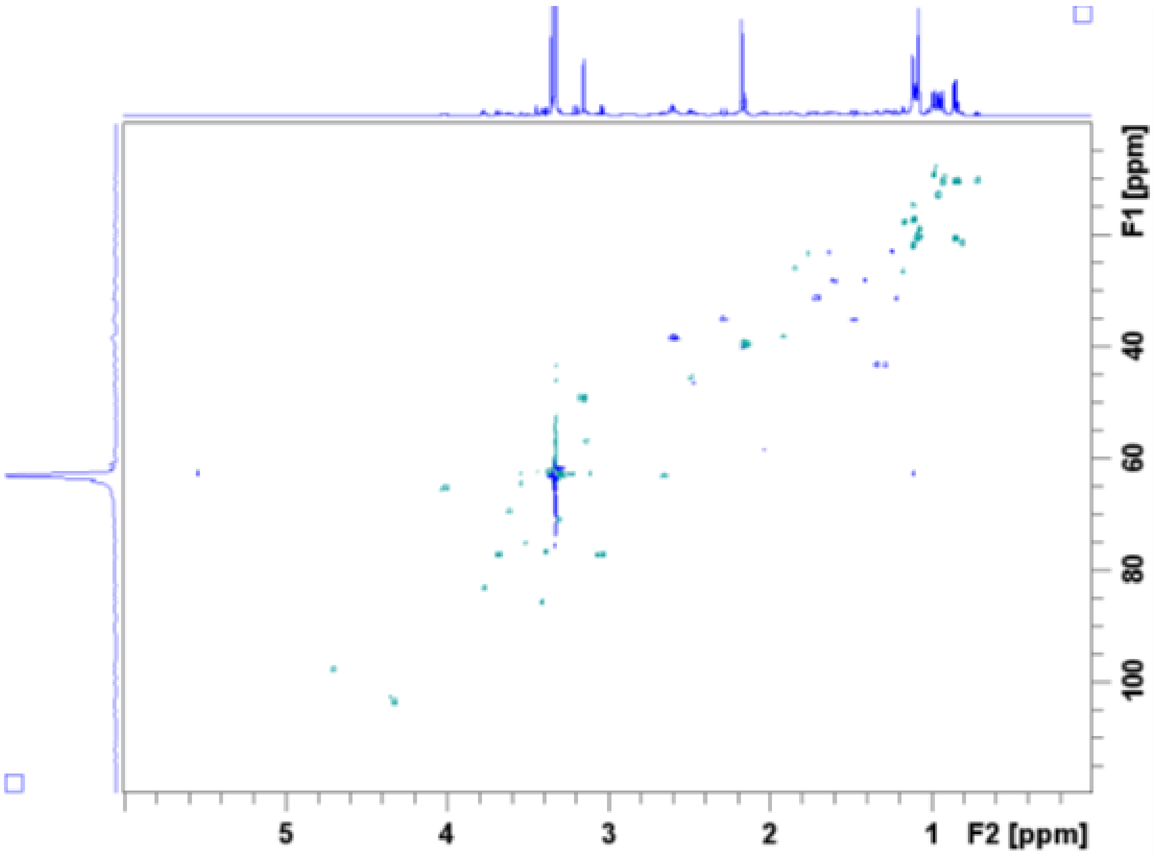

B

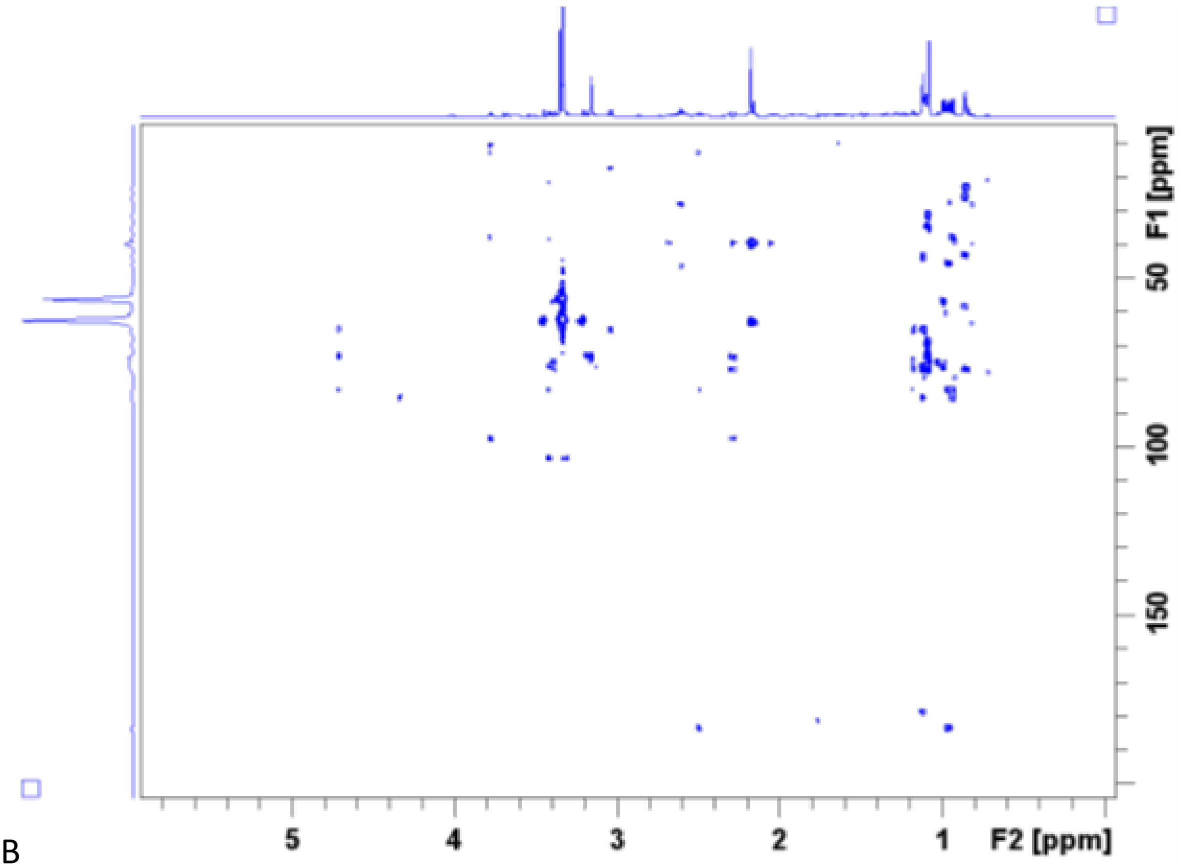

B

Figure S3. A) HSQC and B) HMBC NMR spectra of 9a-aminopropyl-AZI (**2**) recorded in tris-d_{11} buffer (c = 1 mol dm^{-3}, pH 7.4) at 600 MHz and 298 K

CC BY

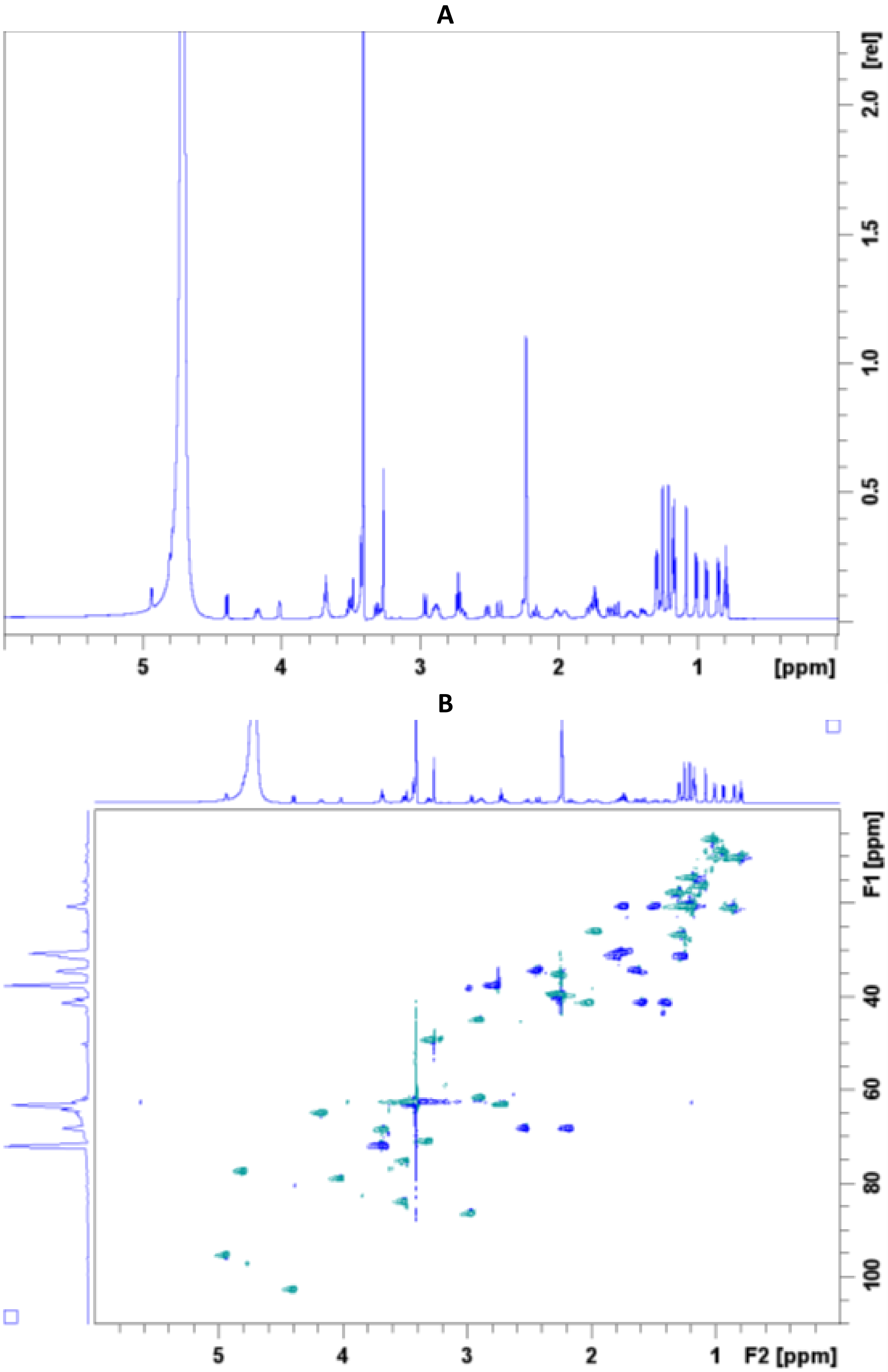

Figure S4. A) ^{1}H and B) HSQC NMR spectra of 4''-aminopropyl-AZI (**34**) recorded in tris-d_{11} buffer (c = 1 mol dm^{-3}, pH 7.4) at 600 MHz and 298 K

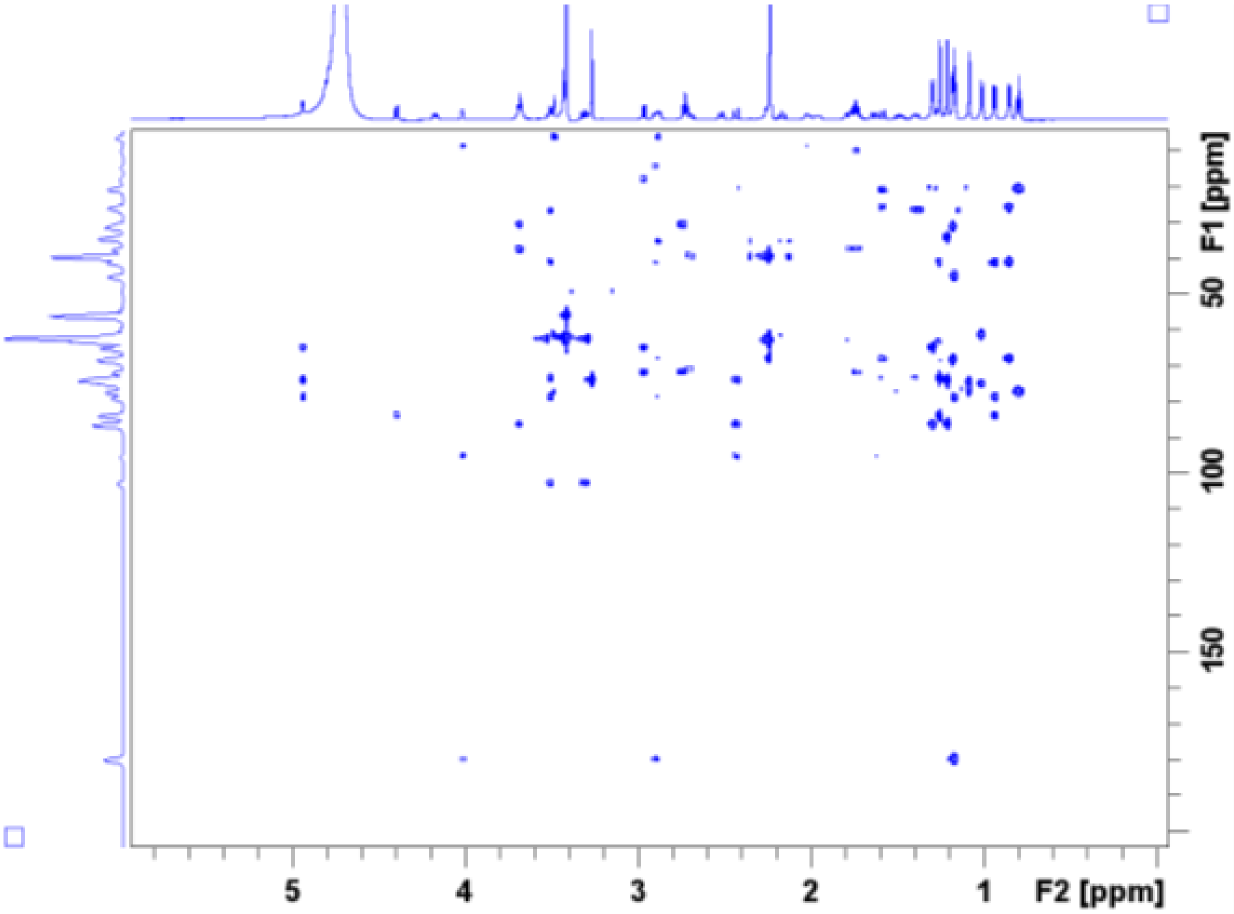

Figure S5. HMBC NMR spectra of 4''-aminopropyl-AZI (**34**) recorded in tris-d_{11} buffer (c = 1 mol dm^{-3}, pH 7.4) at 600 MHz and 298 K

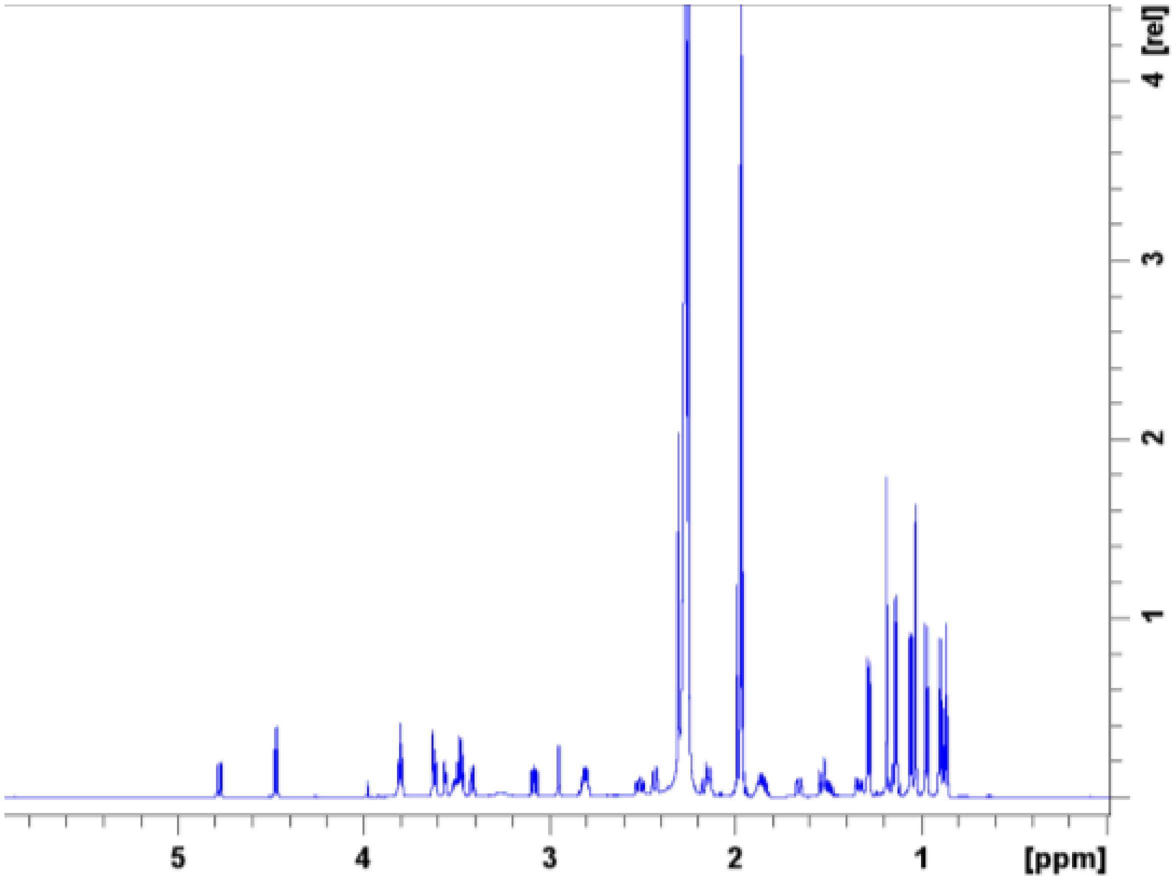

Figure S6. ^{1}H NMR spectra of 3-aminopropyl-AZI (**54**) recorded in acetonitrile-d_3 at 600 MHz and 298 K

(cc) BY

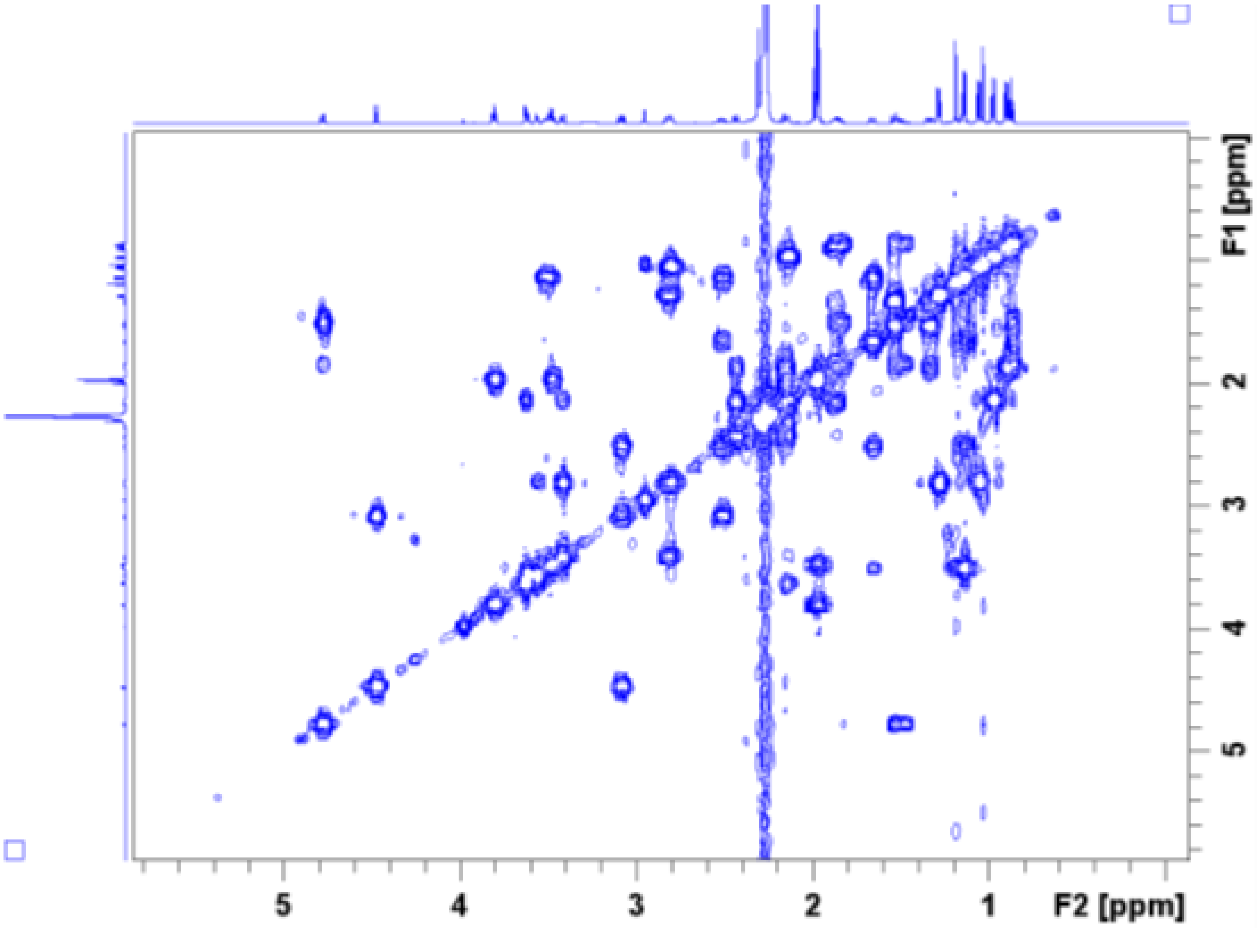

Figure S7. COSY NMR spectra of 3-aminopropyl-AZI (**54**) recorded in acetonitrile-d$_3$ at 600 MHz and 298 K

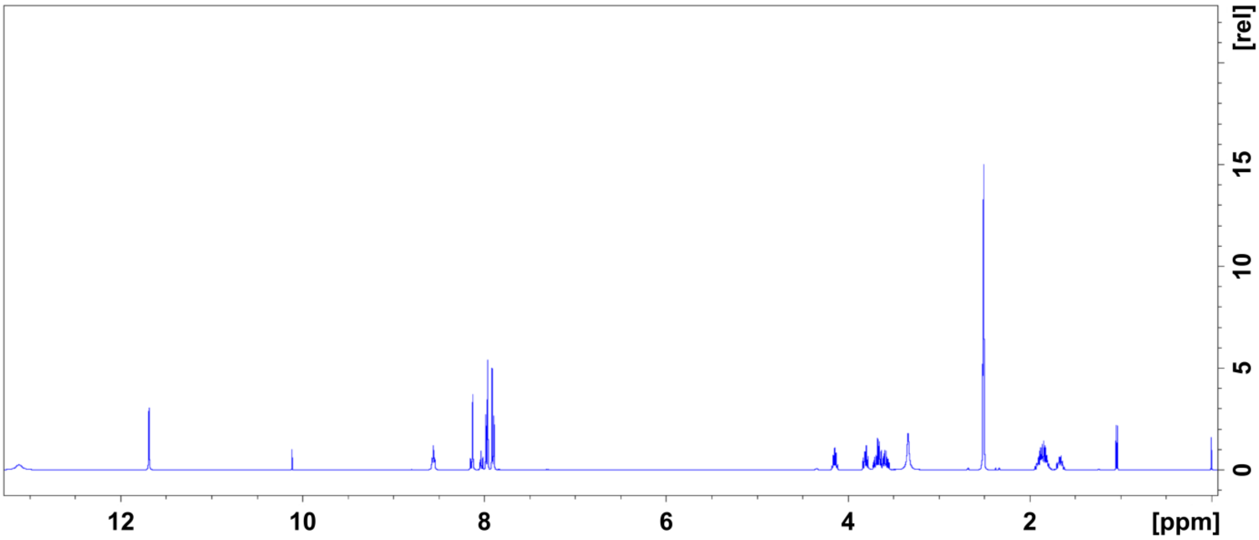

Figure S8. ^{1}H NMR spectra of 3-((2-((tetrahydrofurfuryl)carbamothioyl)hydrazineylidene)methyl)benzoic acid recorded in DMSO-d$_6$ at 600 MHz and 298 K

A

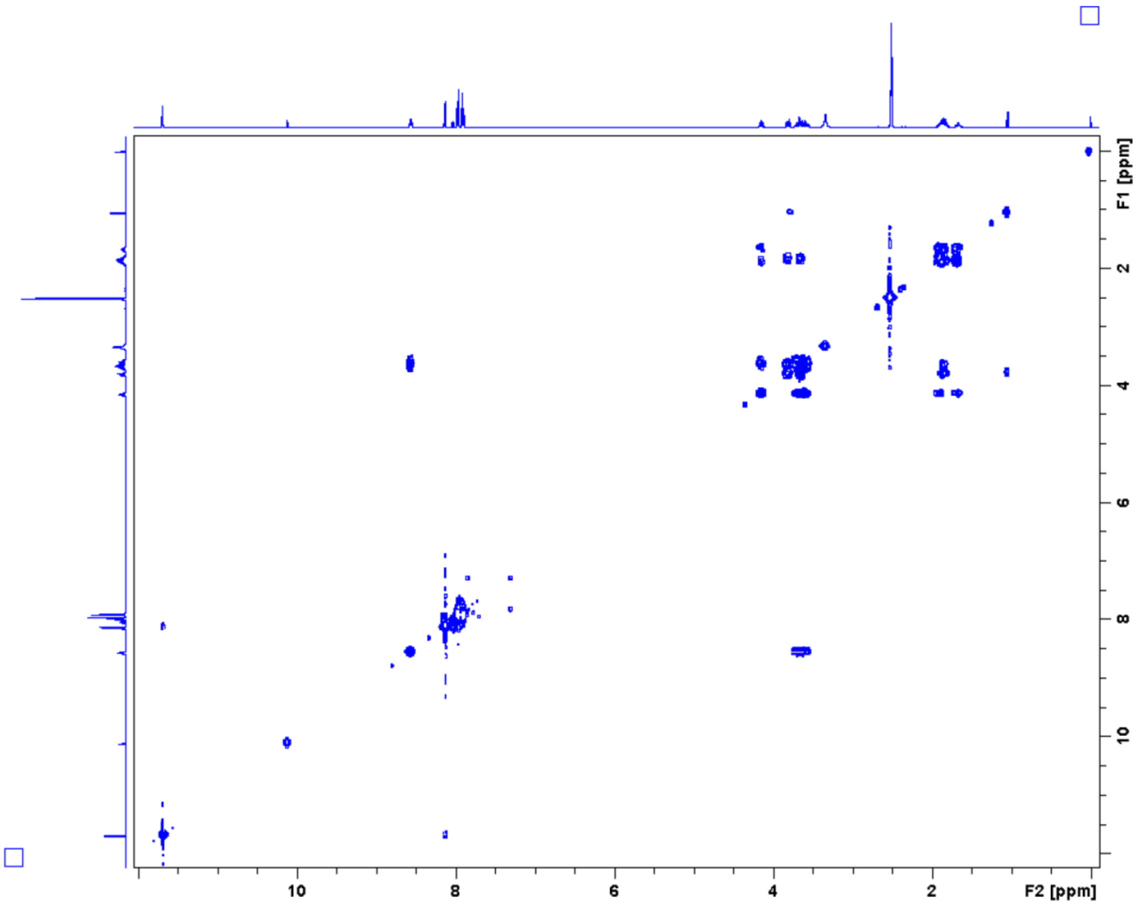

B

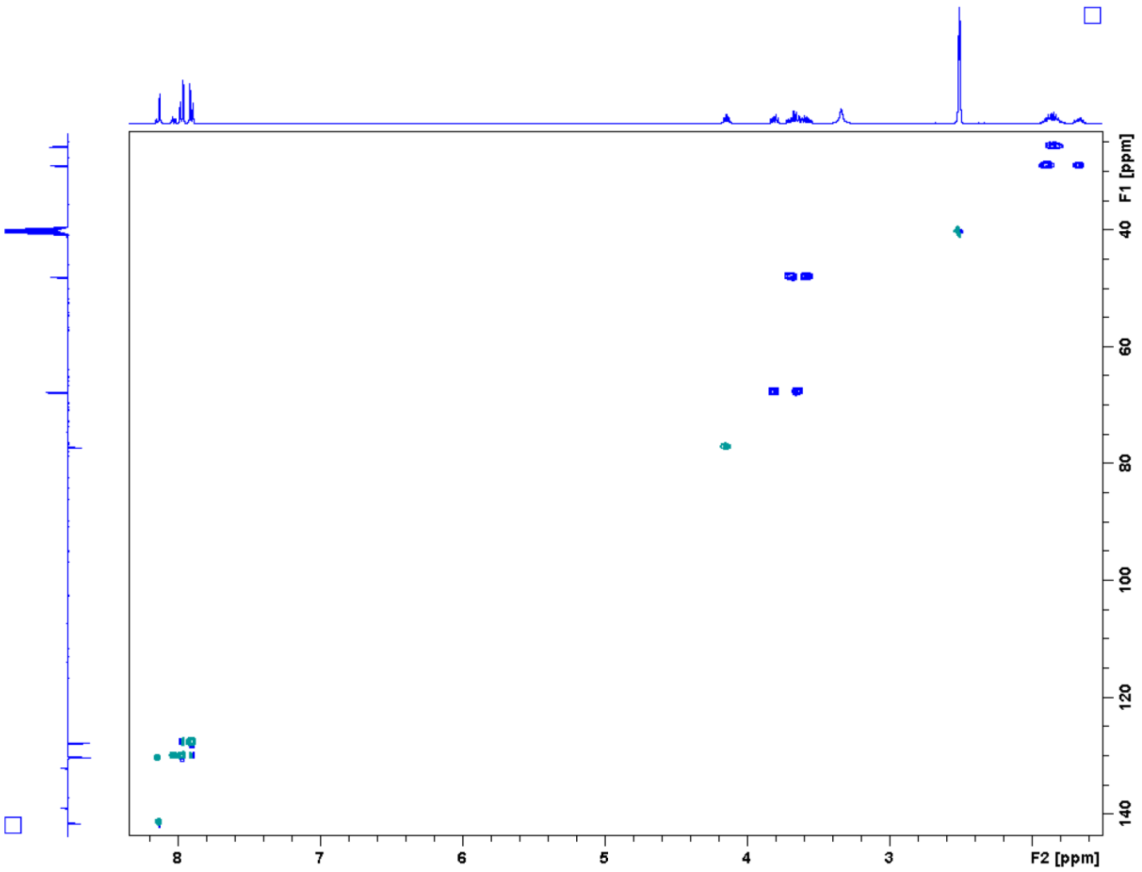

Figure S9. A) COSY and B) HSQC NMR spectra of 3-((2-((tetrahydrofurfuryl)carbamothioyl)hydrazineylidene)methyl)benzoic acid recorded in DMSO-d$_6$ at 600 MHz and 298 K

CC BY

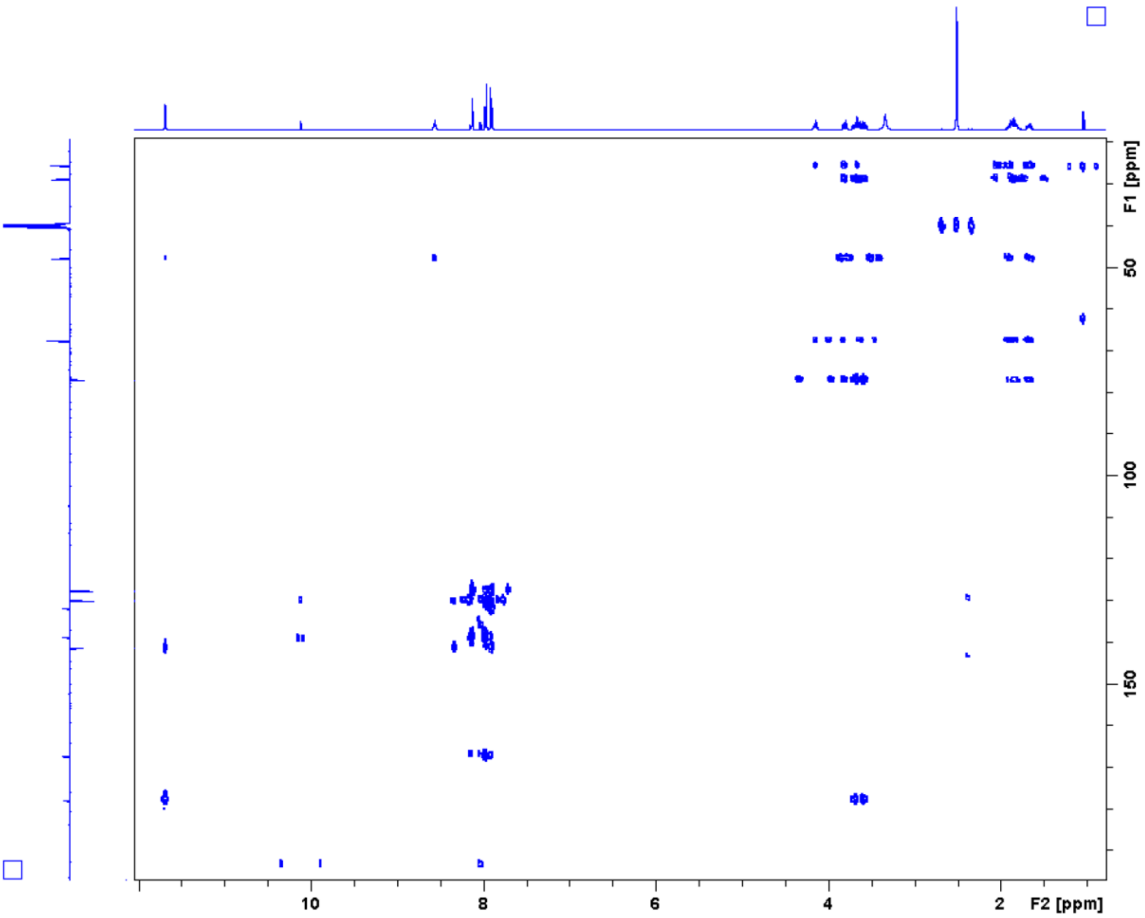

Figure S10. HMBC NMR spectra of 3-((2-((tetrahydrofurfuryl)carbamothioyl)hydrazineylidene)methyl)benzoic acid recorded in DMSO-d_6 at 600 MHz and 298 K

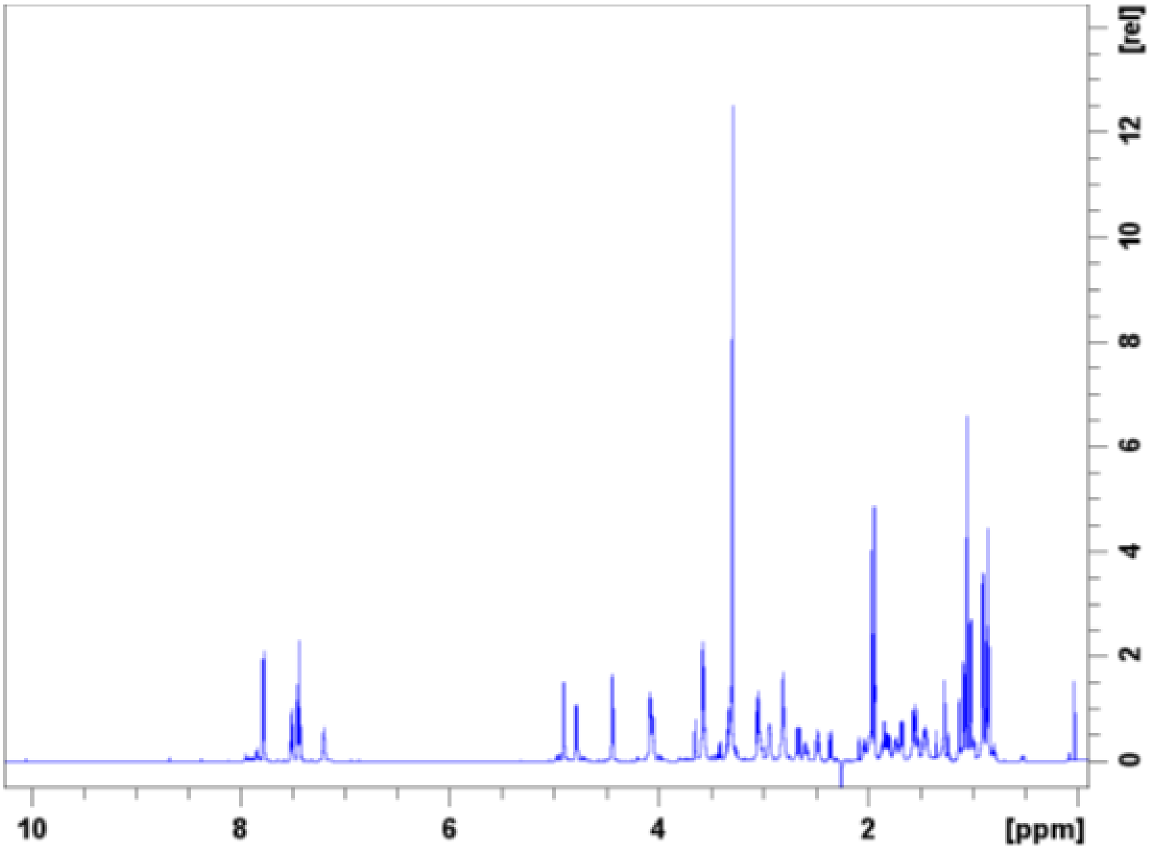

Figure S11. Representative ^{1}H NMR spectra of the compound 9a-4 (**22**) recorded in acetonitrile-d_3 at 600 MHz and 298 K

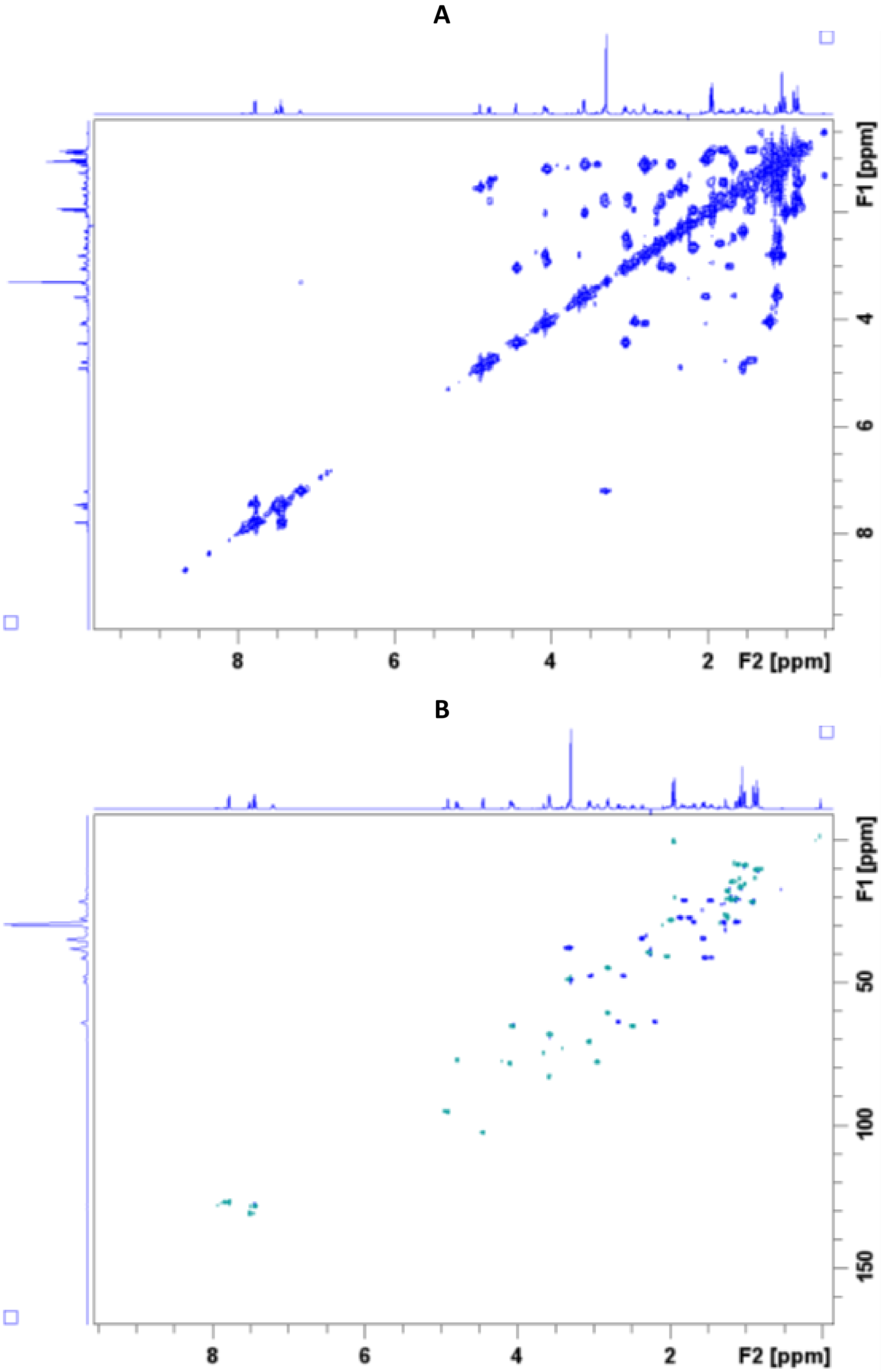

Figure S12. Representative A) COSY and B) HSQC NMR spectra of the compound 9a-4 (**22**) recorded in acetonitrile-d_3 at 600 MHz and 298 K

(cc) BY

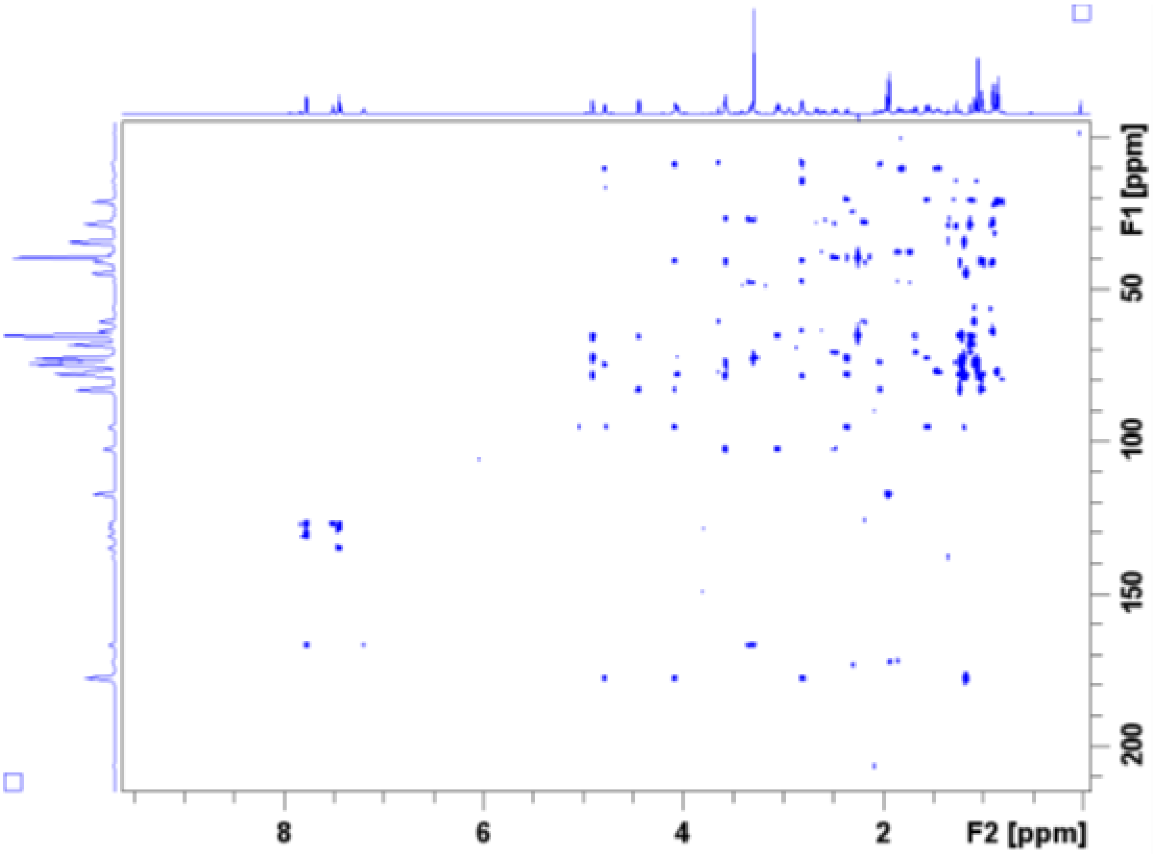

Figure S13. Representative HMBC NMR spectra of the compound 9a-4 (**22**) recorded in acetonitrile-d_3 at 600 MHz and 298 K

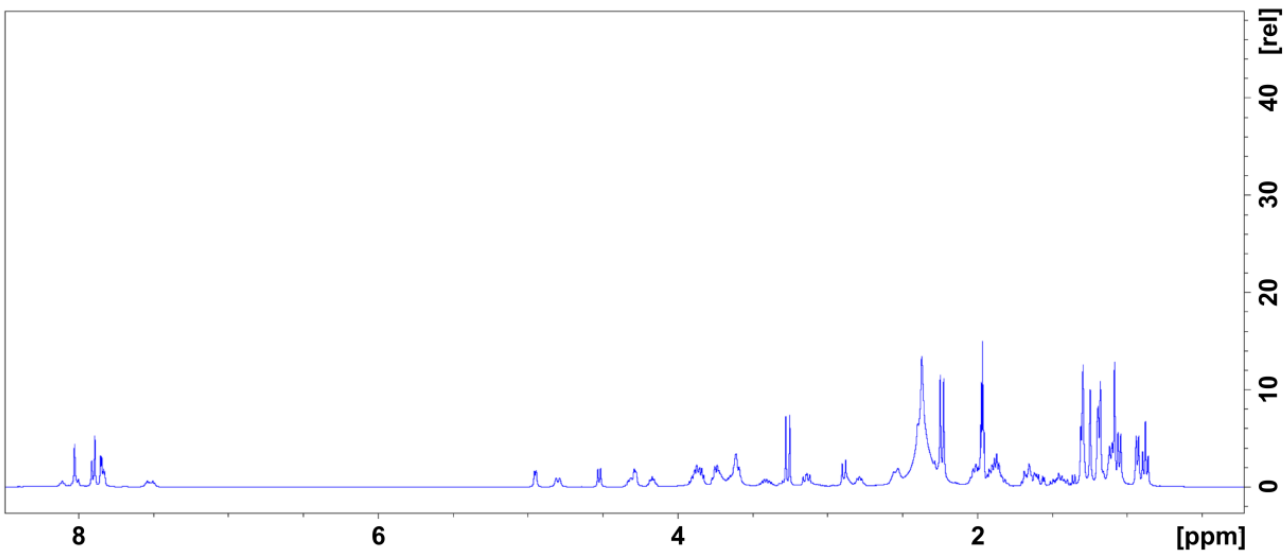

Figure S14. Representative ^{1}H NMR spectra of the compound 4''_j (**43**) recorded in acetonitrile-d_3 at 600 MHz and 298 K

A

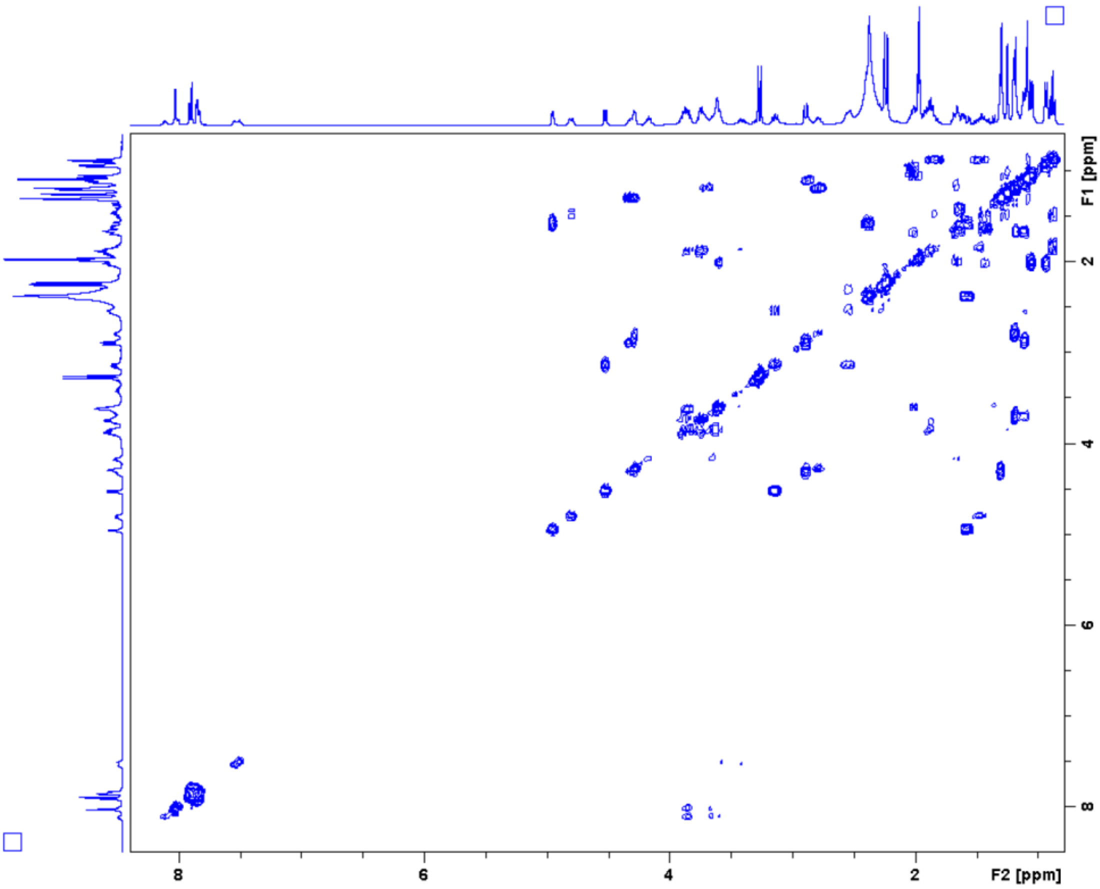

B

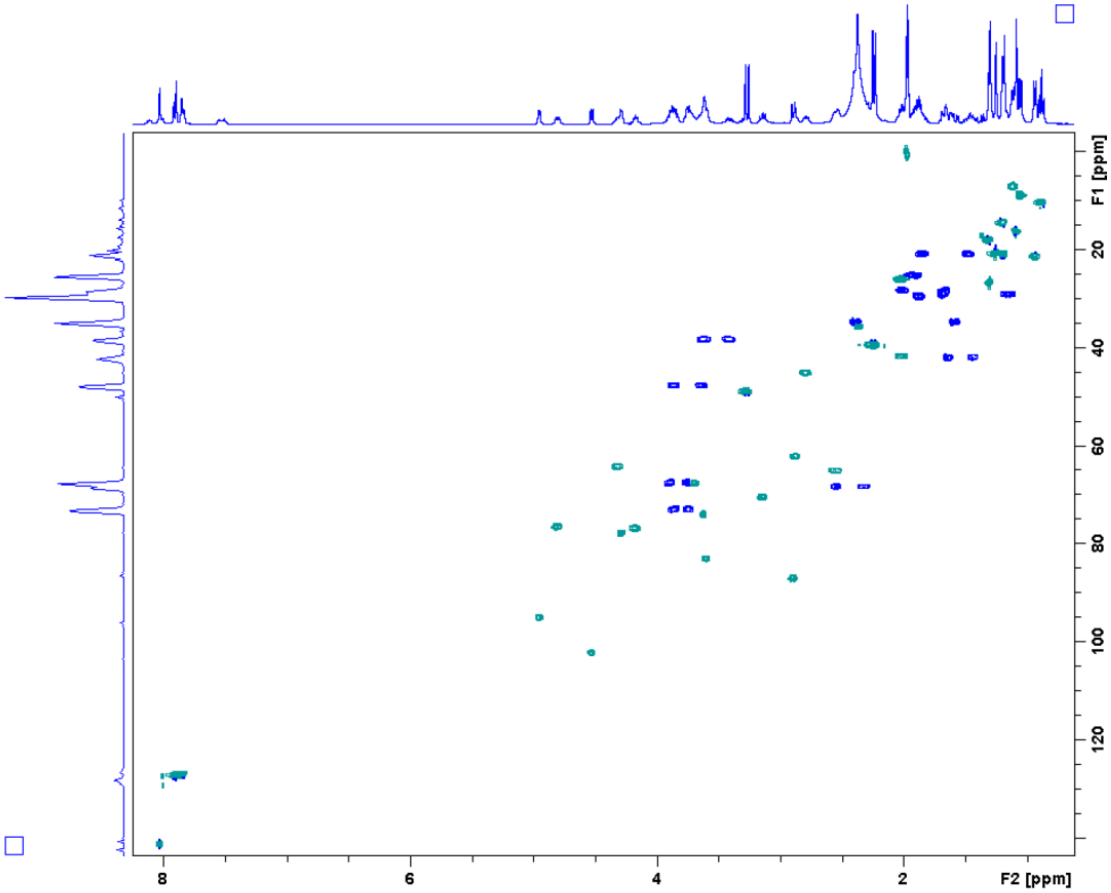

Figure S15. Representative A) COSY and B) HSQC NMR spectra of the compound 4''_j (**43**) recorded in acetonitrile-d_3 at 600 MHz and 298 K

CC BY

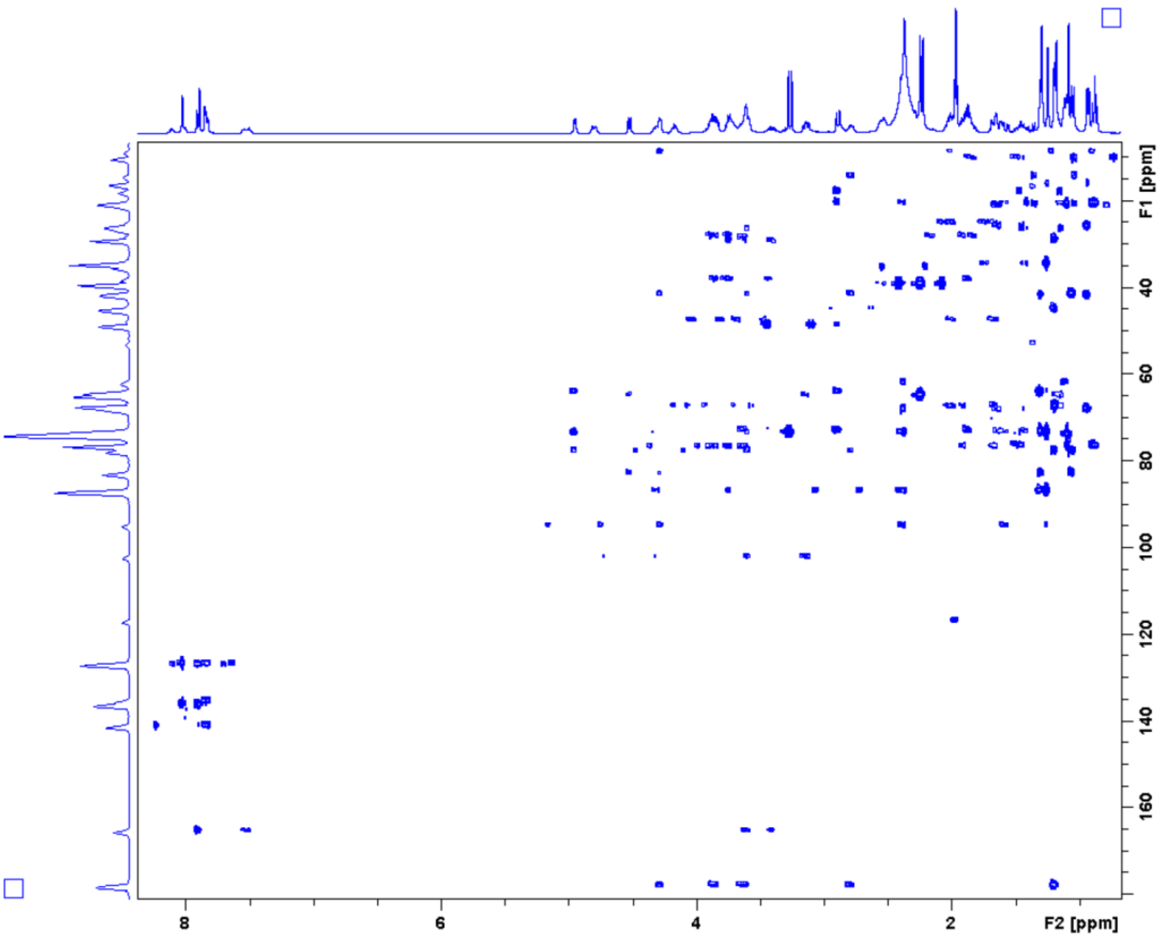

Figure S16. Representative HMBC NMR spectra of the compound 4''_j (**43**) recorded in acetonitrile-d3 at 600 MHz and 298 K

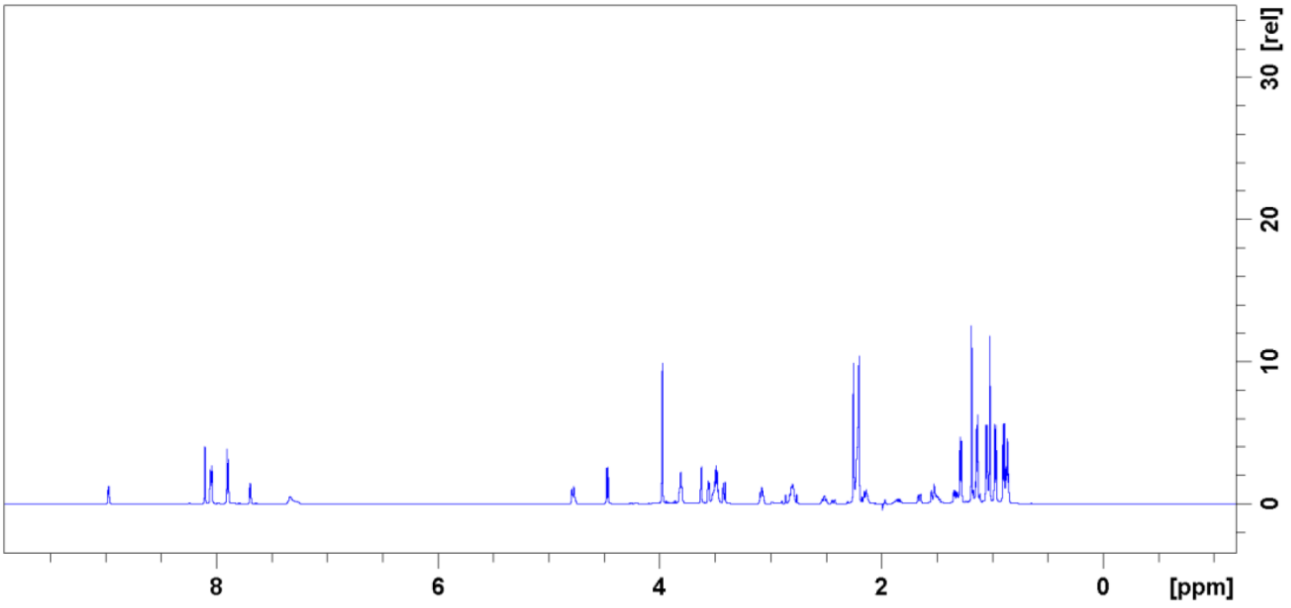

Figure S17. Representative ^{1}H NMR spectra of the compound 3_7 (**68**) recorded in acetonitrile-d3 at 600 MHz and 298 K

A

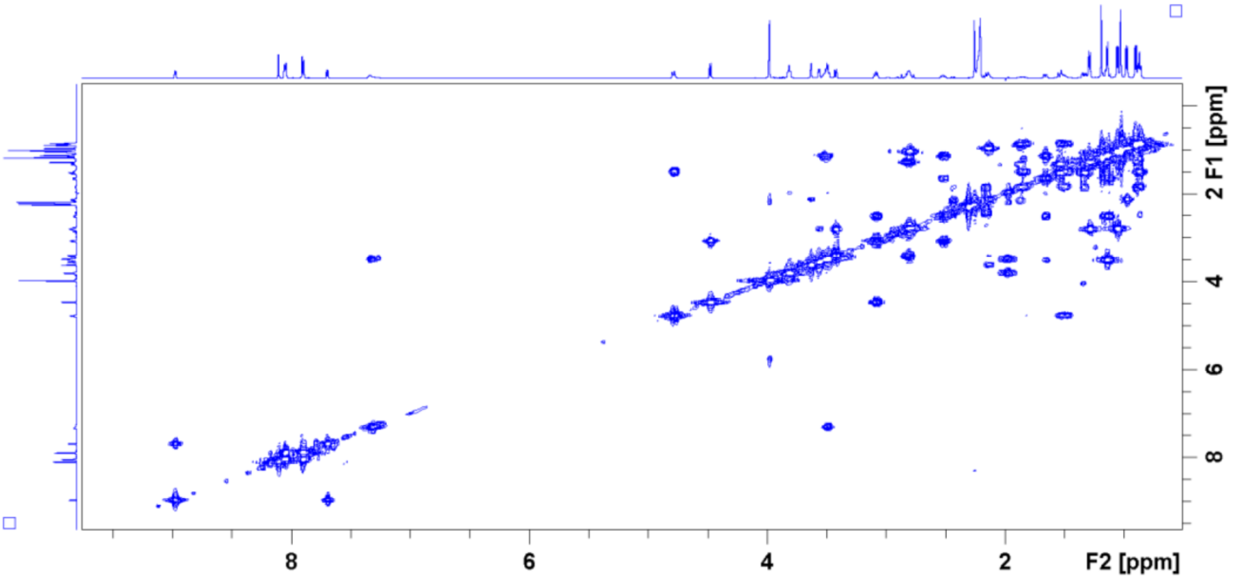

B

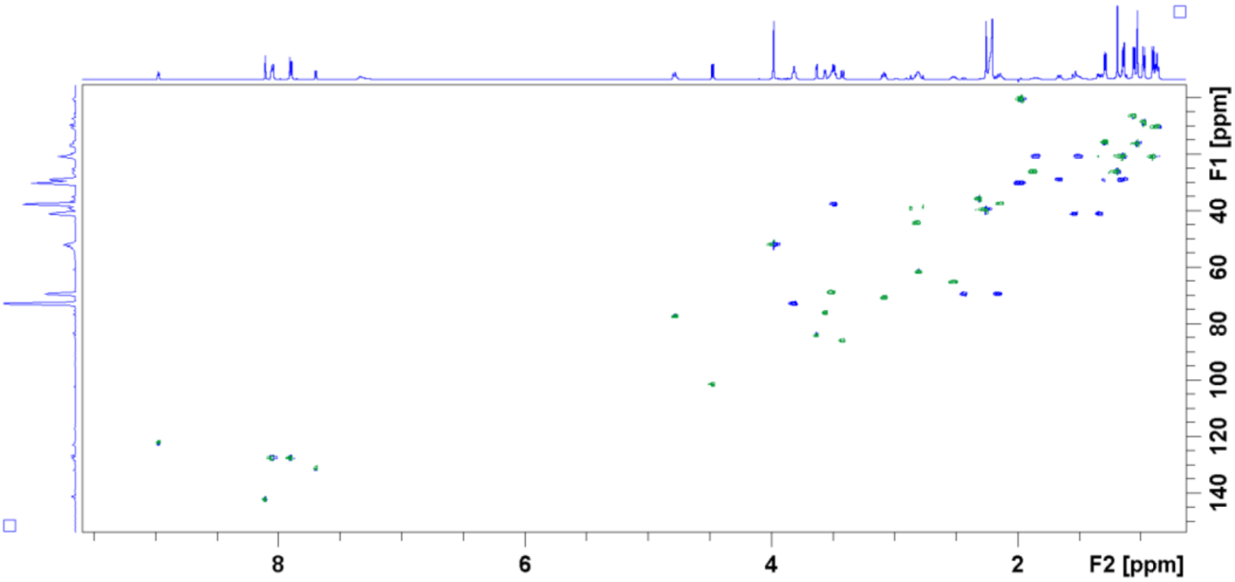

C

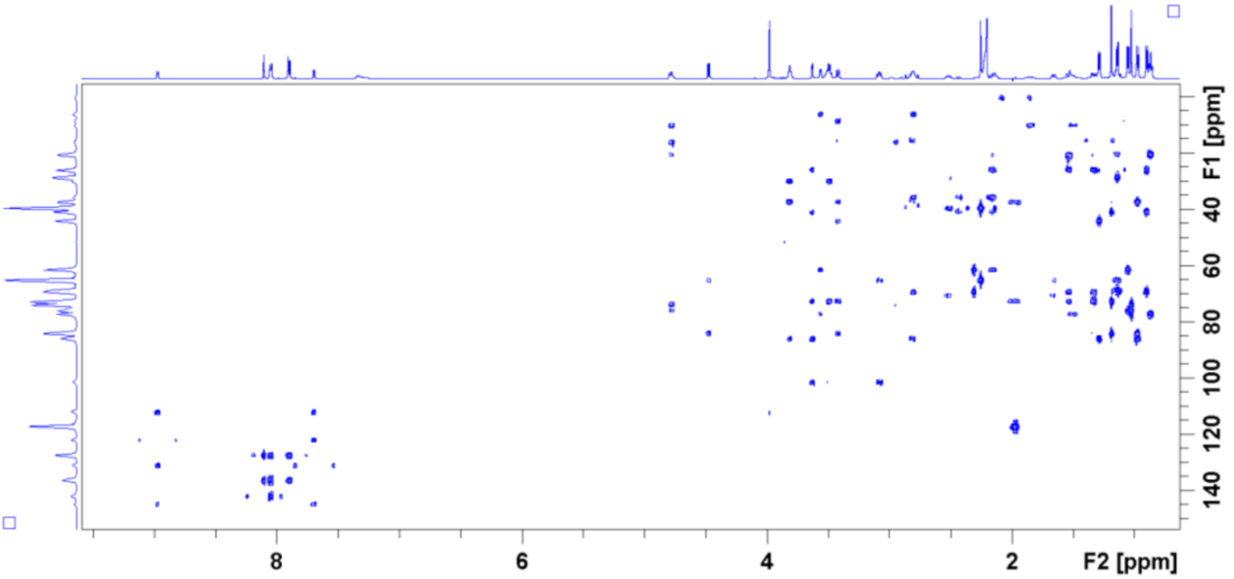

Figure S18. Representative A) COSY, B) HSQC and C) HMBC NMR spectra of the compound 3_7 (**68**) recorded in acetonitrile-d_3 at 600 MHz and 298 K

CC BY

A

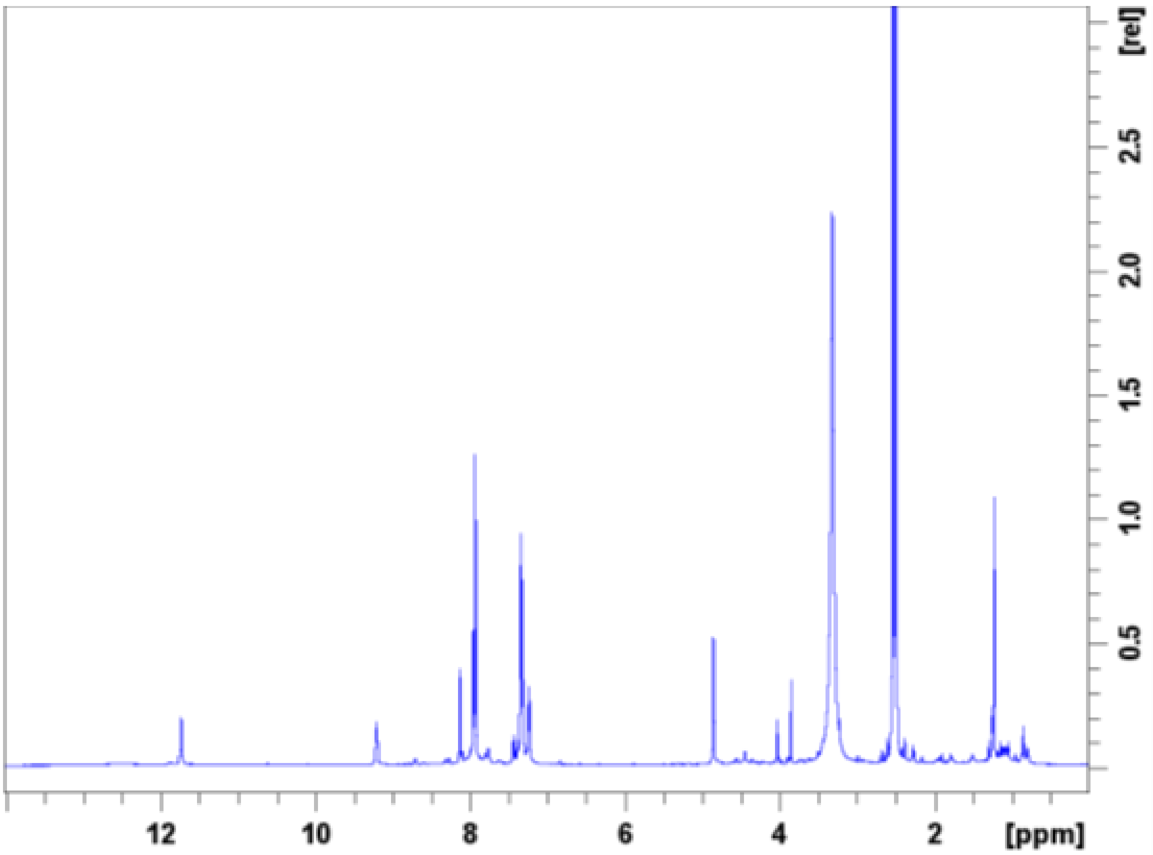

B

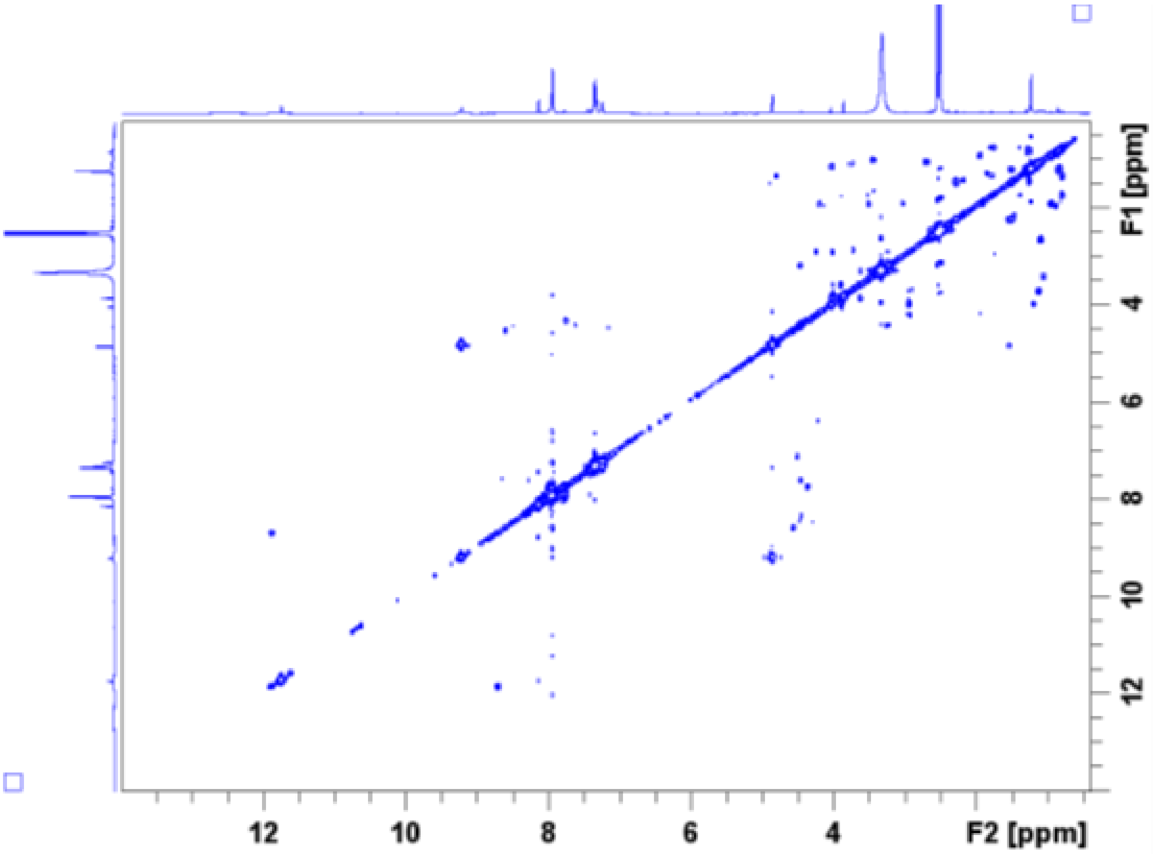

Figure S19. Representative A) ^{1}H and B) COSY NMR spectra of the compound M4''_NiP6 (**76**) recorded in DMSO-d_6
at 600 MHz and 298 K

A

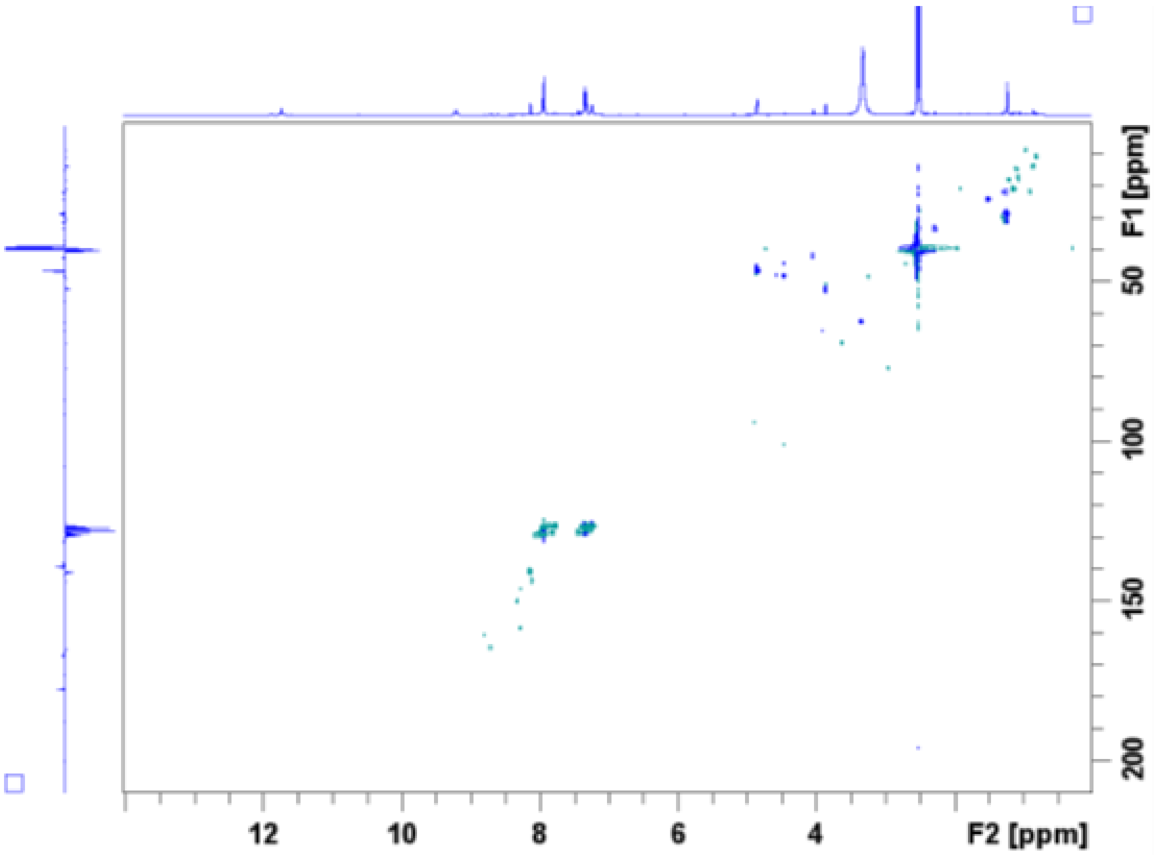

B

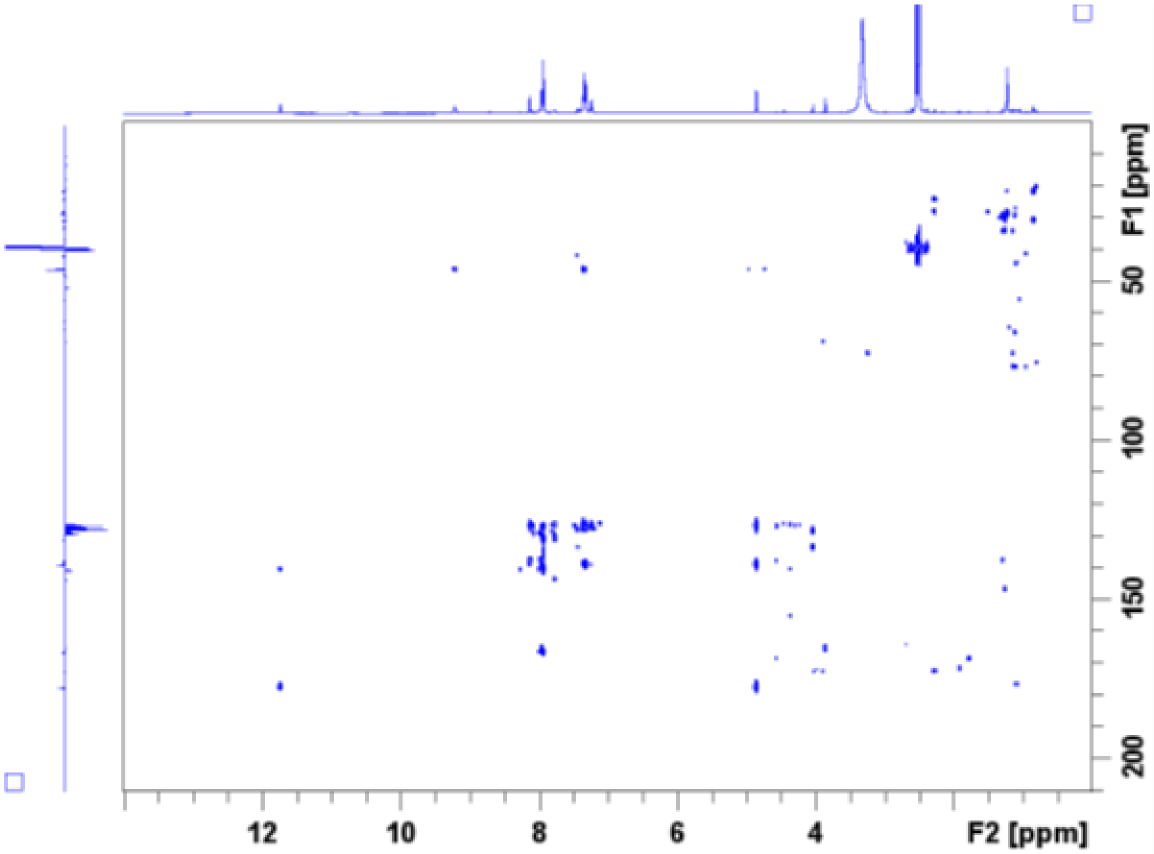

Figure S20. Representative A) HSQC and B) HMBC sNMR pectra of the compound M4''_NiP6 (**76**) recorded in DMSO-d6 at 600 MHz and 298 K

(cc) BY

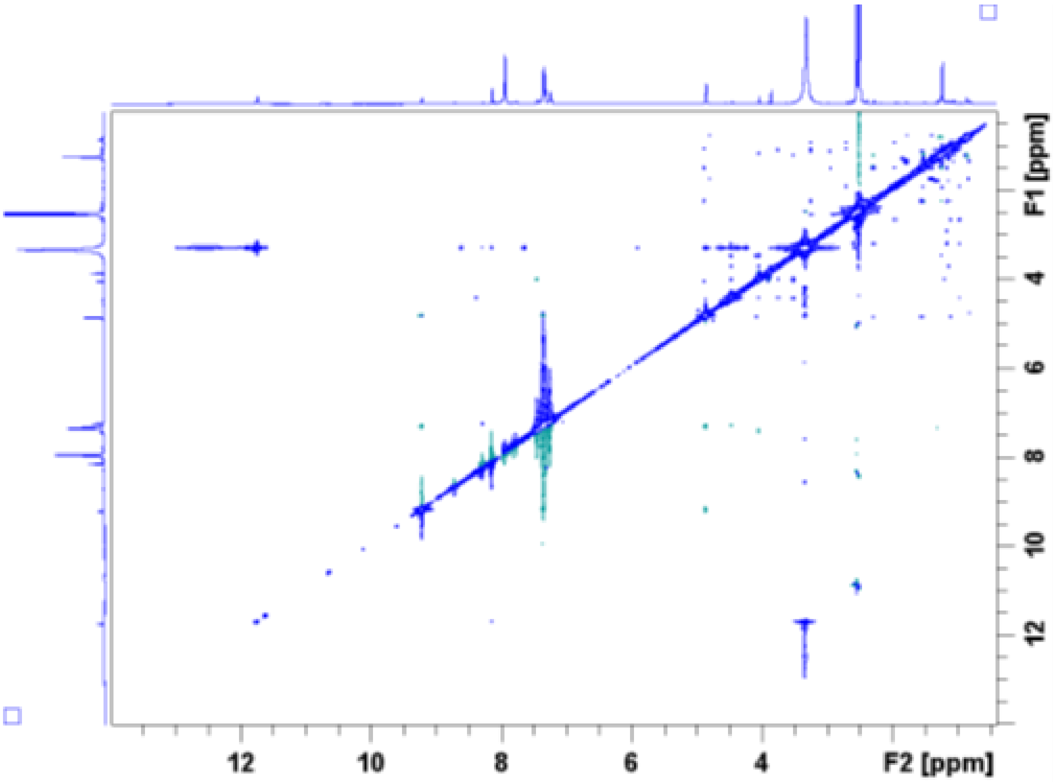

Figure S21. Representative NOESY NMR spectra of the compound M4''_NiP6 (**76**) recorded in DMSO-d_6 at 600 MHz and 298 K

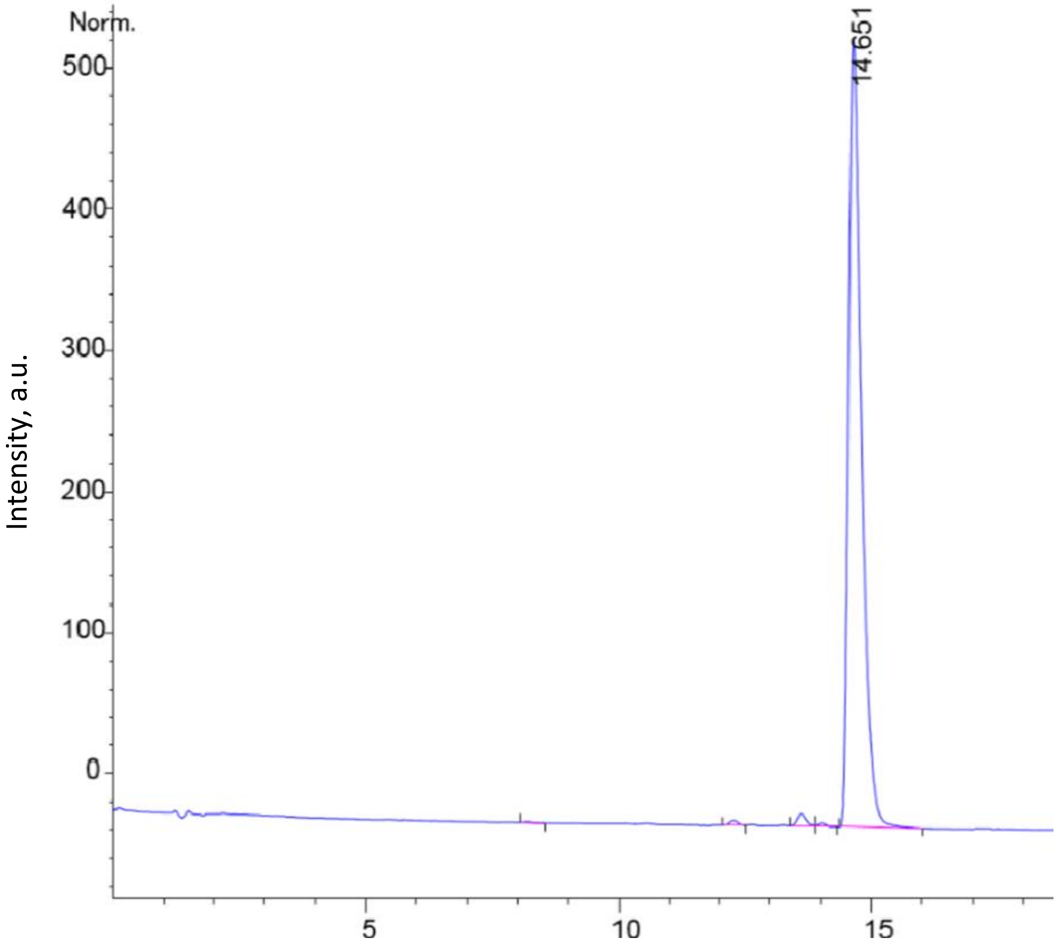

Figure S22. Representative HPLC chromatogram of the compound 9a_m (**12**)

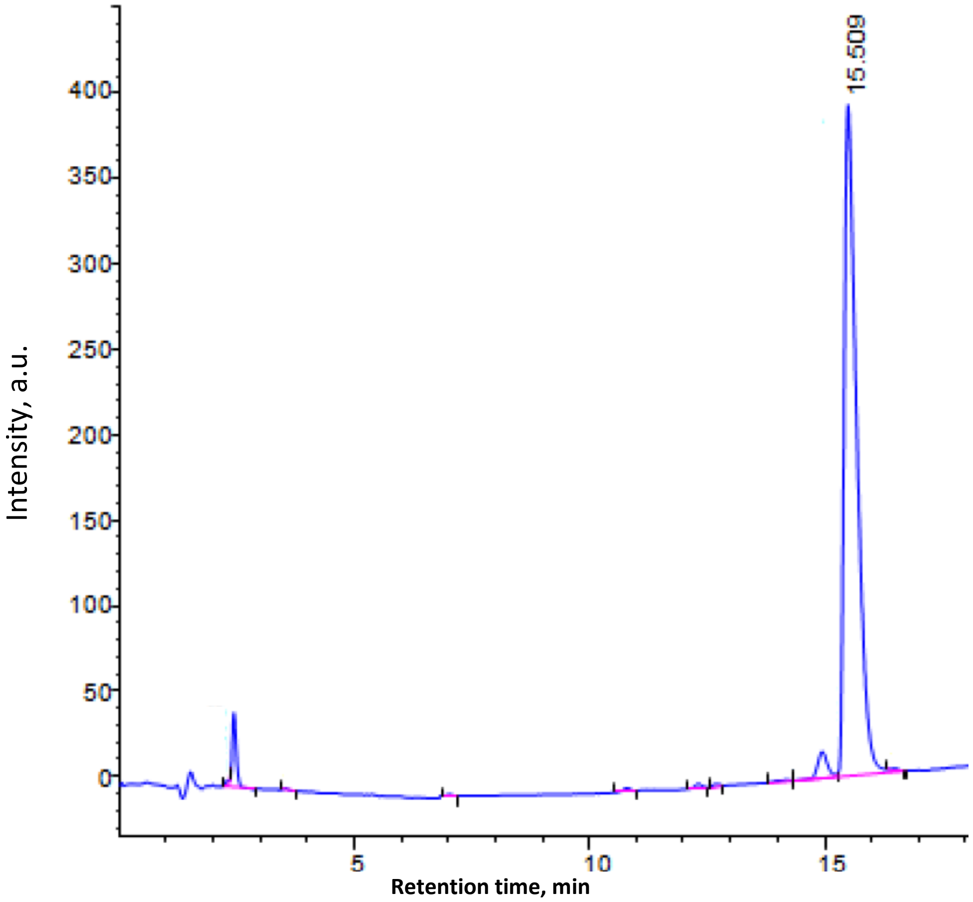

Figure S23. Representative HPLC chromatogram of the compound 4''_l (**45**)

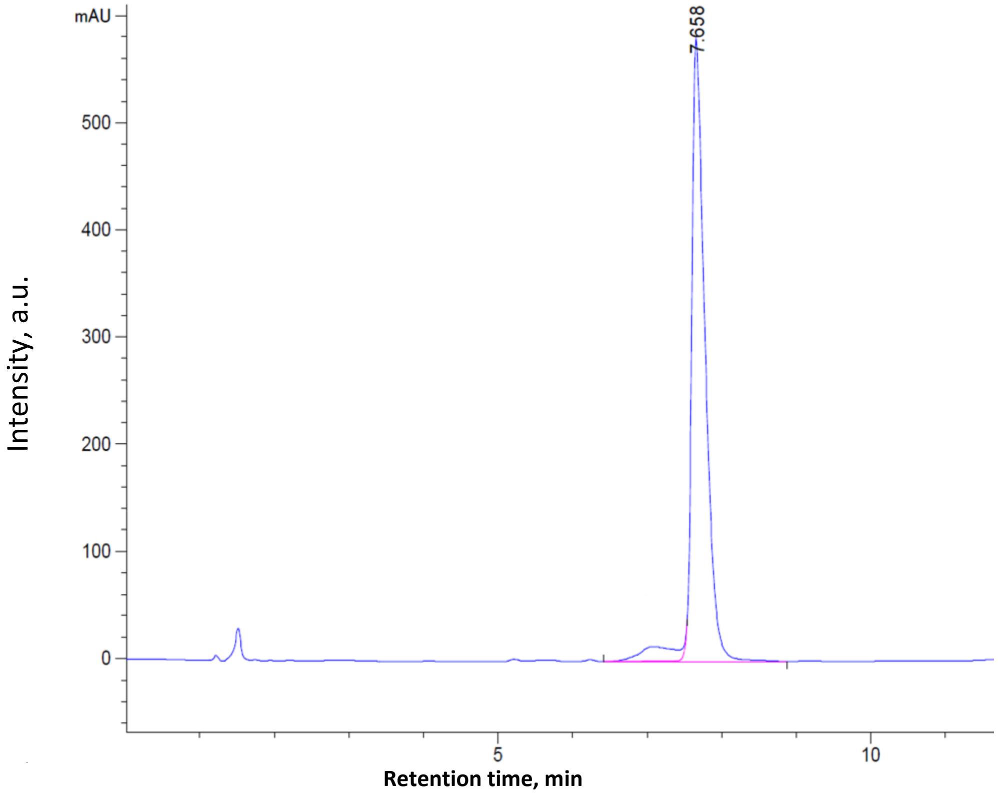

Figure S24. Representative HPLC chromatogram of the compound 3_16 (**61**)

CC BY

Table S1. The 82 compounds used in molecular diversity and SAR analyses - their labels and SMILES

ID_1	ID_2	SMILES
AZI	**1**	CC1C(O)C(O)(C)C(CC)OC(=O)C(C(C(C(C(CC(CN1C)C)(O)C)OC1OC(C)CC(N(C)C)C1O)C)OC1OCC(O)C(OC)(C)C1)C
9a-amino-propyl-AZI	**2**	O=C1OC(CC)C(O)(C)C(O)C(C)N(CCCN)CC(C)CC(O)(C)C(OC2OC(C)CC(N(C)C)C2O)C(C)C(OC2OC(C)C(O)C(C)(OC)C2)C1C
9a_a	**3**	O=C1OC(CC)C(O)(C)C(O)C(C)N(CCCNC(=O)c2cc(C=NNC(=S)Nc3ccccc3)ccc2)CC(C)CC(O)(C)C(OC2OC(C)CC(N(C)C)C2O)C(C)C(OC2OC(C)C(O)C(C)(OC)C2)C1C
9a_b	**4**	O=C1OC(CC)C(O)(C)C(O)C(C)N(CCCNC(=O)c2cc(C=NNC(=S)N)ccc2)CC(C)CC(O)(C)C(OC2OC(C)CC(N(C)C)C2O)C(C)C(OC2OC(C)C(O)C(C)(OC)C2)C1C
9a_c	**5**	O=C1OC(CC)C(O)(C)C(O)C(C)N(CCCNC(=O)c2cc(C=NNC(=S)Nc3ccc(OC)cc3)ccc2)CC(C)CC(O)(C)C(OC2OC(C)CC(N(C)C)C2O)C(C)C(OC2OC(C)C(O)C(C)(OC)C2)C1C
9a_d	**6**	O=C1OC(CC)C(O)(C)C(O)C(C)N(CCCNC(=O)c2cc(C=NNC(=S)Nc3cc(F)ccc3)ccc2)CC(C)CC(O)(C)C(OC2OC(C)CC(N(C)C)C2O)C(C)C(OC2OC(C)C(O)C(C)(OC)C2)C1C
9a_e	**7**	O=C1OC(CC)C(O)(C)C(O)C(C)N(CCCNC(=O)c2cc(C=NNC(=S)Nc3ccc(F)cc3)ccc2)CC(C)CC(O)(C)C(OC2OC(C)CC(N(C)C)C2O)C(C)C(OC2OC(C)C(O)C(C)(OC)C2)C1C
9a_f	**8**	CC1CC(O)(C)C(OC2C(O)C(N(C)C)CC(C)O2)C(C)C(OC2OC(C)C(O)C(C)(OC)C2)C(C)C(=O)OC(C(C(C(N(C1)CCCNC(=O)c1ccc(C=NNC(=S)Nc2ccc(OC(F)(F)F)cc2)cc1)C)O)(C)O)CC
9a_g	**9**	CC1CC(O)(C)C(OC2C(O)C(N(C)C)CC(C)O2)C(C)C(OC2OC(C)C(O)C(C)(OC)C2)C(C)C(=O)OC(C(C(C(N(C1)CCCNC(=O)c1ccc(C=NNC(=S)Nc2c(C)onc2C)cc1)C)O)(C)O)CC
9a_h	**10**	CC1CC(O)(C)C(OC2C(O)C(N(C)C)CC(C)O2)C(C)C(OC2OC(C)C(O)C(C)(OC)C2)C(C)C(=O)OC(C(C(C(N(C1)CCCNC(=O)c1ccc(C=NNC(=S)NCCCc2ccccc2)cc1)C)O)(C)O)CC
9a_i	**11**	CC1CC(O)(C)C(OC2C(O)C(N(C)C)CC(C)O2)C(C)C(OC2OC(C)C(O)C(C)(OC)C2)C(C)C(=O)OC(C(C(C(N(C1)CCCNC(=O)c1ccc(C=NNC(=S)Nc2c(C(=O)OC)scc2)cc1)C)O)(C)O)CC
9a_m	**12**	CC1CC(O)(C)C(OC2C(O)C(N(C)C)CC(C)O2)C(C)C(OC2OC(C)C(O)C(C)(OC)C2)C(C)C(=O)OC(C(C(C(N(C1)CCCNC(=O)c1ccc(C=NNC(=S)Nc2cc3c(cccc3)cc2)cc1)C)O)(C)O)CC
9a_n	**13**	CC1CC(O)(C)C(OC2C(O)C(N(C)C)CC(C)O2)C(C)C(OC2OC(C)C(O)C(C)(OC)C2)C(C)C(=O)OC(C(C(C(N(C1)CCCNC(=O)c1ccc(C=NNC(=S)NCCc2ccccc2)cc1)C)O)(C)O)CC
9a_o	**14**	CC1CC(O)(C)C(OC2C(O)C(N(C)C)CC(C)O2)C(C)C(OC2OC(C)C(O)C(C)(OC)C2)C(C)C(=O)OC(C(C(C(N(C1)CCCNC(=O)c1ccc(C=NNC(=S)Nc2ccc(S(=O)(=O)N3CCCCC3)cc2)cc1)C)O)(C)O)CC
9a_p	**15**	CC1CC(O)(C)C(OC2C(O)C(N(C)C)CC(C)O2)C(C)C(OC2OC(C)C(O)C(C)(OC)C2)C(C)C(=O)OC(C(C(C(N(C1)CCCNC(=O)c1ccc(C=NNC(=S)Nc2ccc3OCOc3c2)cc1)C)O)(C)O)CC
9a_s	**16**	CC1CC(O)(C)C(OC2C(O)C(N(C)C)CC(C)O2)C(C)C(OC2OC(C)C(O)C(C)(OC)C2)C(C)C(=O)OC(C(C(C(N(C1)CCCNC(=O)c1ccc(C=NNC(=S)NCC2CCOC2)cc1)C)O)(C)O)CC
9a_6	**17**	CC1CC(O)(C)C(OC2C(O)C(N(C)C)CC(C)O2)C(C)C(OC2OC(C)C(O)C(C)(OC)C2)C(C)C(=O)OC(C(C(C(N(C1)CCCNC(=O)c1ccc(C=NNC(=S)NCc2ccccc2)cc1)C)O)(C)O)CC
S4_1	**18**	Oc1ccccc1C=NNC(=S)Nc1ccc(cc1)C(=O)NCCCN1C(C)C(O)C(O)(C)C(CC)OC(=O)C(C)C(OC2OCC(O)C(OC)(C)C2)C(C)C(OC2OC(C)CC(N(C)C)C2O)C(O)(C)CC(C)C1
S4_2	**19**	Oc1c(OC)cccc1C=NNC(=S)Nc1ccc(cc1)C(=O)NCCCN1C(C)C(O)C(O)(C)C(CC)OC(=O)C(C)C(OC2OCC(O)C(OC)(C)C2)C(C)C(OC2OC(C)CC(N(C)C)C2O)C(O)(C)CC(C)C1
S4_3	**20**	Oc1cc(O)ccc1C=NNC(=S)Nc1ccc(cc1)C(=O)NCCCN1C(C)C(O)C(O)(C)C(CC)OC(=O)C(C)C(OC2OCC(O)C(OC)(C)C2)C(C)C(OC2OC(C)CC(N(C)C)C2O)C(O)(C)CC(C)C1
9a-2	**21**	C[C@@H]1[C@@H](O)[C@@](O)(C)[C@@H](CC)OC([C@H](C)[C@@H](O[C@H]2C[C@](OC)(C)[C@@H](O)[C@H](C)O2)[C@H](C)[C@@H](O[C@@H]3O[C@H](C)C[C@H](N(C)C)[C@H]3O)[C@@](O)(C)C[C@H](CN1CCCNC(C4=CC=C(/C=N/NC(NC5=CC=CN=C5)=S)C=C4)=O)C)=O
9a-4	**22**	C[C@@H]1[C@@H](O)[C@@](O)(C)[C@@H](CC)OC([C@H](C)[C@@H](O[C@H]2C[C@](OC)(C)[C@@H](O)[C@H](C)O2)[C@H](C)[C@@H](O[C@@H]3O[C@H](C)C[C@H](N(C)C)[C@H]3O)[C@@](O)(C)C[C@H](CN1CCCNC(C4=CC=C(/C=N/NC(NC(C5=CC=CC=C5)=O)=S)C=C4)=O)C)=O
9a-13	**23**	C[C@@H]1[C@@H](O)[C@@](O)(C)[C@@H](CC)OC([C@H](C)[C@@H](O[C@H]2C[C@](OC)(C)[C@@H](O)[C@H](C)O2)[C@H](C)[C@@H](O[C@@H]3O[C@H](C)C[C@H](N(C)C)[C@H]3O)[C@@](O)(C)C[C@H](CN1CCCNC(C4=CC=C(/C=N/NC(NC(OCC)=O)=S)C=C4)=O)C)=O
2'_a	**24**	CCC1C(C)(C(C(N(CC(CC(O)(C(C(C(C(C)C(=O)O1)OC1OC(C(C(OC)(C1)C)O)C)C)OC1C(C(CC(O1)C)N(C)C)OCCCNC(=O)c1ccc(C=NNC(=S)N)cc1)C)C)C)C)O)O
2'_b	**25**	CCC1C(C)(C(C(N(CC(CC(O)(C(C(C(C(C)C(=O)O1)OC1OC(C(C(OC)(C1)C)O)C)C)OC1C(C(CC(O1)C)N(C)C)OCCCNC(=O)c1ccc(C=NNC(=S)Nc2ccccc2)cc1)C)C)C)C)O)O
2'_c	**26**	CCC1C(C)(C(C(N(CC(CC(O)(C(C(C(C(C)C(=O)O1)OC1OC(C(C(OC)(C1)C)O)C)C)OC1C(C(CC(O1)C)N(C)C)OCCCNC(=O)c1ccc(C=NNC(=S)Nc2ccc(OC)cc2)cc1)C)C)C)C)O)O
2'_d	**27**	CCC1C(C)(C(C(N(CC(CC(O)(C(C(C(C(C)C(=O)O1)OC1OC(C(C(OC)(C1)C)O)C)C)OC1C(C(CC(O1)C)N(C)C)OCCCNC(=O)c1ccc(C=NNC(=S)Nc2ccc(F)cc2)cc1)C)C)C)C)O)O
2'_e	**28**	CCC1C(C)(C(C(N(CC(CC(O)(C(C(C(C(C)C(=O)O1)OC1OC(C(C(OC)(C1)C)O)C)C)OC1C(C(CC(O1)C)N(C)C)OCCCNC(=O)c1ccc(C=NNC(=S)Nc2cc(F)ccc2)cc1)C)C)C)C)O)O
3'_a	**29**	CCC1C(C)(C(C(N(CC(CC(O)(C(C(C(C(C)C(=O)O1)OC1OC(C(C(OC)(C1)C)O)C)C)OC1C(C(CC(O1)C)NC(=O)c1ccc(C=NNC(=S)N)cc1)O)C)C)C)C)O)O
3'_b	**30**	CCC1C(C)(C(C(N(CC(CC(O)(C(C(C(C(C)C(=O)O1)OC1OC(C(C(OC)(C1)C)O)C)C)OC1C(C(CC(O1)C)NC(=O)c1ccc(C=NNC(=S)Nc2ccccc2)cc1)O)C)C)C)C)O)O

ID_1	ID_2	SMILES
3'_c	**31**	CCC1C(C(C(N(CC(CC(O)(C(C(C(C(C)C(=O)O1)OC1OC(C(C(OC)(C1)C)O)C)C)OC1C(C(CC(O1)C)NC(=O)c1ccc(C=NNC(=S)Nc2ccc(OC)cc2)cc1)O)C)C)C)C)O)O
3'_d	**32**	CCC1C(C)(C(C(N(CC(CC(O)(C(C(C(C(C)C(=O)O1)OC1OC(C(C(OC)(C1)C)O)C)C)OC1C(C(CC(O1)C)NC(=O)c1ccc(C=NNC(=S)Nc2ccc(F)cc2)cc1)O)C)C)C)C)O)O
3'_e	**33**	CCC1C(C(C(N(CC(CC(O)(C(C(C(C(C)C(=O)O1)OC1OC(C(C(OC)(C1)C)O)C)C)OC1C(C(CC(O1)C)NC(=O)c1ccc(C=NNC(=S)Nc2cccc(F)c2)cc1)O)C)C)C)C)O)O
4''-amino-propyl-AZI	**34**	O=C1OC(CC)C(O)(C)C(O)C(C)N(C)CC(C)CC(O)(C)C(OC2OC(C)CC(N(C)C)C2O)C(C)C(OC2OC(C)C(OCCCN)C(C)(OC)C2)C1C
4''_b	**35**	O=C1OC(CC)C(O)(C)C(O)C(C)N(C)CC(C)CC(O)(C)C(OC2OC(C)CC(N(C)C)C2O)C(C)C(OC2OC(C)C(OCCCNC(=O)c3ccc(C=NNC(=S)Nc4ccccc4)cc3)C(C)(OC)C2)C1C
4''_c	**36**	O=C1OC(CC)C(O)(C)C(O)C(C)N(C)CC(C)CC(O)(C)C(OC2OC(C)CC(N(C)C)C2O)C(C)C(OC2OC(C)C(OCCCNC(=O)c3ccc(C=NNC(=S)Nc4ccc(OC)cc4)cc3)C(C)(OC)C2)C1C
4''_d	**37**	O=C1OC(CC)C(O)(C)C(O)C(C)N(C)CC(C)CC(O)(C)C(OC2OC(C)CC(N(C)C)C2O)C(C)C(OC2OC(C)C(OCCCNC(=O)c3ccc(C=NNC(=S)Nc4ccc(F)cc4)cc3)C(C)(OC)C2)C1C
4''_e	**38**	O=C1OC(CC)C(O)(C)C(O)C(C)N(C)CC(C)CC(O)(C)C(OC2OC(C)CC(N(C)C)C2O)C(C)C(OC2OC(C)C(OCCCNC(=O)c3ccc(C=NNC(=S)Nc4cccc(F)c4)cc3)C(C)(OC)C2)C1C
4''_f	**39**	O=C1OC(CC)C(O)(C)C(O)C(C)N(C)CC(C)CC(O)(C)C(OC2OC(C)CC(N(C)C)C2O)C(C)C(OC2OC(C)C(OCCCNC(=O)c3cc(C=NNC(=S)Nc4ccc(OC(F)(F)F)cc4)ccc3)C(C)(OC)C2)C1C
4''_g	**40**	O=C1OC(CC)C(O)(C)C(O)C(C)N(C)CC(C)CC(O)(C)C(OC2OC(C)CC(N(C)C)C2O)C(C)C(OC2OC(C)C(OCCCNC(=O)c3cc(C=NNC(=S)Nc4cc5ccccc5cc4)ccc3)C(C)(OC)C2)C1C
4''_h	**41**	O=C1OC(CC)C(O)(C)C(O)C(C)N(C)CC(C)CC(O)(C)C(OC2OC(C)CC(N(C)C)C2O)C(C)C(OC2OC(C)C(OCCCNC(=O)c3cc(C=NNC(=S)NCc4ccccc4)ccc3)C(C)(OC)C2)C1C
4''_i	**42**	O=C1OC(CC)C(O)(C)C(O)C(C)N(C)CC(C)CC(O)(C)C(OC2OC(C)CC(N(C)C)C2O)C(C)C(OC2OC(C)C(OCCCNC(=O)c3cc(C=NNC(=S)Nc4c(C(=O)OC)scc4)ccc3)C(C)(OC)C2)C1C
4''_j	**43**	O=C1OC(CC)C(O)(C)C(O)C(C)N(C)CC(C)CC(O)(C)C(OC2OC(C)CC(N(C)C)C2O)C(C)C(OC2OC(C)C(OCCCNC(=O)c3cc(C=NNC(=S)NCC4CCCO4)ccc3)C(C)(OC)C2)C1C
4''_k	**44**	O=C1OC(CC)C(O)(C)C(O)C(C)N(C)CC(C)CC(O)(C)C(OC2OC(C)CC(N(C)C)C2O)C(C)C(OC2OC(C)C(OCCCNC(=O)c3cc(C=NNC(=S)Nc4cccnc4)ccc3)C(C)(OC)C2)C1C
4''_l	**45**	O=C1OC(CC)C(O)(C)C(O)C(C)N(C)CC(C)CC(O)(C)C(OC2OC(C)CC(N(C)C)C2O)C(C)C(OC2OC(C)C(OCCCNC(=O)c3cc(C=NNC(=S)Nc4cc5OCOc5cc4)ccc3)C(C)(OC)C2)C1C
4''_o	**46**	O=C1OC(CC)C(O)(C)C(O)C(C)N(C)CC(C)CC(O)(C)C(OC2OC(C)CC(N(C)C)C2O)C(C)C(OC2OC(C)C(OCCCNC(=O)c3cc(C=NNC(=S)Nc4ccc(CS(=O)(=O)N5CCCCC5)cc4)ccc3)C(C)(OC)C2)C1C
4''_1	**47**	O=C1OC(CC)C(O)(C)C(O)C(C)N(C)CC(C)CC(O)(C)C(OC2OC(C)CC(N(C)C)C2O)C(C)C(OC2OC(C)C(OCCCNC(=O)c3cc(C=NNC(=S)NCCc4ccccc4)ccc3)C(C)(OC)C2)C1C
4''_9	**48**	O=C1OC(CC)C(O)(C)C(O)C(C)N(C)CC(C)CC(O)(C)C(OC2OC(C)CC(N(C)C)C2O)C(C)C(OC2OC(C)C(OCCCNC(=O)c3cc(C=NNC(=S)NCCCc4ccccc4)ccc3)C(C)(OC)C2)C1C
4''_H6	**49**	CC1C(O)C(O)(C)C(CC)OC(=O)C(C)C(OC2CC(OC)(C)C(OCCCNC(=O)c3ccc(O)c(C=NNC(=S)NCc4ccccc4)c3)C(C)O2)C(C)C(OC2OC(C)CC(N(C)C)C2O)C(O)(C)CC(CN1C)C
4''_H12	**50**	CC1C(O)C(O)(C)C(CC)OC(=O)C(C)C(OC2CC(OC)(C)C(OCCCNC(=O)c3ccc(O)c(C=NNC(=S)Nc4ccc5OCOc5c4)c3)C(C)O2)C(C)C(OC2OC(C)CC(N(C)C)C2O)C(O)(C)CC(CN1C)C
4''_H7	**51**	CC1C(O)C(O)(C)C(CC)OC(=O)C(C)C(OC2CC(OC)(C)C(OCCCNC(=O)c3ccc(O)c(C=NNC(=S)Nc4c(C(=O)OC)scc4)c3)C(C)O2)C(C)C(OC2OC(C)CC(N(C)C)C2O)C(O)(C)CC(CN1C)C
4''_4abaR4	**52**	CC1C(O)C(O)(C)C(CC)OC(=O)C(C)C(OC2CC(OC)(C)C(OCCCNC(=O)c3ccc(NC(=S)NN=Cc4ccccc4O)cc3)C(C)O2)C(C)C(OC2OC(C)CC(N(C)C)C2O)C(O)(C)CC(CN1C)C
4''_3FS6	**53**	CC1C(O)C(O)(C)C(CC)OC(=O)C(C)C(OC2CC(OC)(C)C(OCCCNC(=O)c3cccc(C=NNC(=S)NCc4ccccc4)c3O)C(C)O2)C(C)C(OC2OC(C)CC(N(C)C)C2O)C(O)(C)CC(CN1C)C
3-amino-propyl-AZI	**54**	O=C1OC(CC)C(O)(C)C(O)C(C)N(C)CC(C)CC(O)(C)C(OC2OC(C)CC(N(C)C)C2O)C(C)C(OCCCN)C1C
3_a	**55**	O=C1OC(CC)C(O)(C)C(O)C(C)N(C)CC(C)CC(O)(C)C(OC2OC(C)CC(N(C)C)C2O)C(C)C(OCCCNC(=O)c2ccc(C=NNC(=S)N)cc2)C1C
3_b	**56**	O=C1OC(CC)C(O)(C)C(O)C(C)N(C)CC(C)CC(O)(C)C(OC2OC(C)CC(N(C)C)C2O)C(C)C(OCCCNC(=O)c2ccc(C=NNC(=S)Nc3ccccc3)cc2)C1C
3_c	**57**	O=C1OC(CC)C(O)(C)C(O)C(C)N(C)CC(C)CC(O)(C)C(OC2OC(C)CC(N(C)C)C2O)C(C)C(OCCCNC(=O)c2ccc(C=NNC(=S)Nc3ccc(OC)cc3)cc2)C1C
3_d	**58**	O=C1OC(CC)C(O)(C)C(O)C(C)N(C)CC(C)CC(O)(C)C(OC2OC(C)CC(N(C)C)C2O)C(C)C(OCCCNC(=O)c2ccc(C=NNC(=S)Nc3ccc(F)cc3)cc2)C1C
3_e	**59**	O=C1OC(CC)C(O)(C)C(O)C(C)N(C)CC(C)CC(O)(C)C(OC2OC(C)CC(N(C)C)C2O)C(C)C(OCCCNC(=O)c2ccc(C=NNC(=S)Nc3cc(F)ccc3)cc2)C1C
3_5	**60**	O=C1OC(CC)C(O)(C)C(O)C(C)N(C)CC(C)CC(O)(C)C(OC2OC(C)CC(N(C)C)C2O)C(C)C(OCCCNC(=O)c2ccc(C=NNC(=S)Nc3cc4ccccc4cc3)cc2)C1C
3_16	**61**	O=C1OC(CC)C(O)(C)C(O)C(C)N(C)CC(C)CC(O)(C)C(OC2OC(C)CC(N(C)C)C2O)C(C)C(OCCCNC(=O)c2ccc(C=NNC(=S)NCC3CCCO3)cc2)C1C
3_10	**62**	O=C1OC(CC)C(O)(C)C(O)C(C)N(C)CC(C)CC(O)(C)C(OC2OC(C)CC(N(C)C)C2O)C(C)C(OCCCNC(=O)c2ccc(C=NNC(=S)Nc3ccc(S(=O)(=O)N4CCCCC4)cc3)cc2)C1C

CC BY

ID_1	ID_2	SMILES
3_1	**63**	O=C1OC(CC)C(O)(C)C(O)C(C)N(C)CC(C)CC(O)(C)C(OC2OC(C)CC(N(C)C)C2O)C(C)C(OCCCNC(=O)c2ccc(C=NNC(=S)N CCc3ccccc3)cc2)C1C
3_9	**64**	O=C1OC(CC)C(O)(C)C(O)C(C)N(C)CC(C)CC(O)(C)C(OC2OC(C)CC(N(C)C)C2O)C(C)C(OCCCNC(=O)c2ccc(C=NNC(=S)N CCCc3ccccc3)cc2)C1C
3_6	**65**	O=C1OC(CC)C(O)(C)C(O)C(C)N(C)CC(C)CC(O)(C)C(OC2OC(C)CC(N(C)C)C2O)C(C)C(OCCCNC(=O)c2ccc(C=NNC(=S)N Cc3ccccc3)cc2)C1C
3_8	**66**	O=C1OC(CC)C(O)(C)C(O)C(C)N(C)CC(C)CC(O)(C)C(OC2OC(C)CC(N(C)C)C2O)C(C)C(OCCCNC(=O)c2ccc(C=NNC(=S)N c3ccc(OC(F)(F)F)cc3)cc2)C1C
3_12	**67**	O=C1OC(CC)C(O)(C)C(O)C(C)N(C)CC(C)CC(O)(C)C(OC2OC(C)CC(N(C)C)C2O)C(C)C(OCCCNC(=O)c2ccc(C=NNC(=S)N c3cc4OCOc4cc3)cc2)C1C
3_7	**68**	O=C1OC(CC)C(O)(C)C(O)C(C)N(C)CC(C)CC(O)(C)C(OC2OC(C)CC(N(C)C)C2O)C(C)C(OCCCNC(=O)c2ccc(C=NNC(=S)N c3c(C(=O)OC)scc3)cc2)C1C
M4''_NiP12	**69**	O=C(O)c1ccc(C=N2N=C(Nc3cc4OCOc4cc3)[S-][Ni+2]22[S-]C(=NN2=Cc2ccc(cc2)C(=O)NCCCOC2C(C)OC(OC3C(C)C(=O)OC(CC)C(O)(C)C(O)C(C)N(C)CC(C)CC(C)(O)C(OC4OC (C)CC(N(C)C)C4O)C3C)CC2(C)OC)Nc2cc3OCOc3cc2)cc1
M4''_NiPS	**70**	CC1(OC)CC(OC2C(C)C(=O)OC(CC)C(O)(C)C(O)C(C)N(C)CC(C)CC(C)(O)C(OC3OC(C)CC(N(C)C)C3O)C2C)OC(C)C1OCC CNC(=O)c1ccc(C=N2N=C([S-][Ni+2]22[S-]C(=NN2=Cc2ccc(cc2)C(O)=O)Nc2cccc3ccccc23)Nc2cccc3ccccc23)cc1
M4''_NiP7	**71**	OC(=O)c1ccc(C=N2N=C(Nc3ccsc3C(=O)OC)[S-][Ni+2]22[S-]C(=NN2=Cc2ccc(cc2)C(=O)NCCCOC2C(C)OC(OC3C(C)C(=O)OC(CC)C(O)(C)C(O)C(C)N(C)CC(C)CC(C)(O)C(OC4OC(C) CC(N(C)C)C4O)C3C)CC2(C)OC)Nc2ccsc2C(=O)OC)cc1
M4''_NiP16	**72**	OC(=O)c1ccc(C=N2N=C([S-][Ni+2]22[S-]C(=NN2=Cc2ccc(cc2)C(=O)NCCCOC2C(C)OC(OC3C(C)C(=O)OC(CC)C(O)(C)C(O)C(C)N(C)CC(C)CC(C)(O)C(OC4OC(C) CC(N(C)C)C4O)C3C)CC2(C)OC)NCC2COCC2)NCC2COCC2)cc1
M4''_NiH6	**73**	CC1(OC)CC(OC2C(C)C(=O)OC(CC)C(O)(C)C(O)C(C)N(C)CC(C)CC(C)(O)C(OC3OC(C)CC(N(C)C)C3O)C2C)OC(C)C1OCC CNC(=O)c1cc2C=N3N=C([S-][Ni+2]3[O-]c2cc1)NCc1ccccc1
M4''_Ni_4ab aR4	**74**	CC1(OC)CC(OC2C(C)C(=O)OC(CC)C(O)(C)C(O)C(C)N(C)CC(C)CC(C)(O)C(OC3OC(C)CC(N(C)C)C3O)C2C)OC(C)C1OCC CNC(=O)c1ccc(NC=2[S-][Ni+2]3[O-]c4ccccc4C=N3N=2)cc1
M4''_Ni3FS6	**75**	CC1(OC)CC(OC2C(C)C(=O)OC(CC)C(O)(C)C(O)C(C)N(C)CC(C)CC(C)(O)C(OC3OC(C)CC(N(C)C)C3O)C2C)OC(C)C1OCC CNC(=O)c1cccc2C=N3N=C([S-][Ni+2]3[O-]c12)NCc1ccccc1
M4''_NiP6	**76**	OC(=O)c1ccc(C=N2N=C([S-][Ni+2]22[S-]C(=NN2=Cc2ccc(cc2)C(=O)NCCCOC2C(C)OC(OC3C(C)C(=O)OC(CC)C(O)(C)C(O)C(C)N(C)CC(C)CC(C)(O)C(OC4OC(C) CC(N(C)C)C4O)C3C)CC2(C)OC)NCc2ccccc2)NCc2ccccc2)cc1
M9a_1_Cu	**77**	CC1(O)C(O)C(C)N(CC(C)CC(C)(O)C(OC2OC(C)CC(N(C)C)C2O)C(C)C(OC2OCC(O)C(C)(OC)C2)C(C)C(=O)OC1CC)CCCN C(=O)c1ccc(NC=2[S-][Cu+2]3[O-]c4ccccc4C=N3N=2)cc1
M9a_2_Cu	**78**	CC1(O)C(O)C(C)N(CC(C)CC(C)(O)C(OC2OC(C)CC(N(C)C)C2O)C(C)C(OC2OCC(O)C(C)(OC)C2)C(C)C(=O)OC1CC)CCCN C(=O)c1ccc(NC=2[S-][Cu+2]3[O-]c4c(O)cccc4C=N3N=2)cc1
M9a_3_Cu	**79**	CC1(O)C(O)C(C)N(CC(C)CC(C)(O)C(OC2OC(C)CC(N(C)C)C2O)C(C)C(OC2OCC(O)C(C)(OC)C2)C(C)C(=O)OC1CC)CCCN C(=O)c1ccc(NC=2[S-][Cu+2]3[O-]c4cc(O)ccc4C=N3N=2)cc1
M9a_1_Ni	**80**	CC1(O)C(O)C(C)N(CC(C)CC(C)(O)C(OC2OC(C)CC(N(C)C)C2O)C(C)C(OC2OCC(O)C(C)(OC)C2)C(C)C(=O)OC1CC)CCCN C(=O)c1ccc(NC=2[S-][Ni+2]3[O-]c4ccccc4C=N3N=2)cc1
M9a_2_Ni	**81**	CC1(O)C(O)C(C)N(CC(C)CC(C)(O)C(OC2OC(C)CC(N(C)C)C2O)C(C)C(OC2OCC(O)C(C)(OC)C2)C(C)C(=O)OC1CC)CCCN C(=O)c1ccc(NC=2[S-][Ni+2]3[O-]c4c(O)cccc4C=N3N=2)cc1
M9a_3_Ni	**82**	CC1(O)C(O)C(C)N(CC(C)CC(C)(O)C(OC2OC(C)CC(N(C)C)C2O)C(C)C(OC2OCC(O)C(C)(OC)C2)C(C)C(=O)OC1CC)CCCN C(=O)c1ccc(NC=2[S-][Ni+2]3[O-]c4cc(O)ccc4C=N3N=2)cc1

Table S2. The 82 compounds used in molecular diversity and SAR analyses - their labels and antibacterial activities

			S_pyog_B0542	S_aureus_29213	S_aureus_B0331	S_aureus_B0330	S_pneum_B0326	S_pneum_B0633	E_faecalis_29212	S_cerevisiae_7752	E_coli_25922
ID_1	ID_2	Class	MIC, µg mL^{-1}								
AZI	**1**	AZI	0.125	1	128	128	8	128	8	128	8
9a-aminopropyl-AZI	**2**	core	0.5	8	128	128	64	128	64	128	32
9a_a	**3**	9a	0.125	16	128	128	32	128	128	128	64
9a_b	**4**	9a	0.125	8	128	128	32	128	32	128	64
9a_c	**5**	9a	0.125	8	128	128	16	128	32	128	64
9a_d	**6**	9a	0.125	8	128	128	16	128	32	128	32
9a_e	**7**	9a	0.125	8	128	128	16	128	16	128	128
9a_f	**8**	9a	4	64	32	32	128	64	8	128	64
9a_g	**9**	9a	0.5	64	128	128	128	128	128	128	128
9a_h	**10**	9a	0.5	8	128	128	64	64	16	128	64
9a_i	**11**	9a	2	4	128	128	128	128	16	128	64
9a_m	**12**	9a	0.5	16	128	128	128	128	32	128	128
9a_n	**13**	9a	0.5	8	128	128	64	64	16	128	64
9a_o	**14**	9a	1	16	128	128	128	128	32	128	64
9a_p	**15**	9a	0.25	128	128	128	64	128	64	128	128
9a_s	**16**	9a	0.125	16	128	128	64	128	128	128	64
9a_6	**17**	9a	0.125	8	128	128	128	128	32	128	32
S4_1	**18**	9a	1	8	128	128	32	128	32	128	64
S4_2	**19**	9a	1	16	128	128	32	128	32	128	64
S4_3	**20**	9a	0.5	8	128	128	32	128	32	32	64
9a-2	**21**	9a	2	32	128	128	128	128	128	128	128
9a-4	**22**	9a	0.5	4	128	128	32	128	32	128	64
9a-13	**23**	9a	0.5	2	128	128	32	128	32	128	32
2'_a	**24**	2'	8	128	128	128	128	128	128	128	128
2'_b	**25**	2'	8	128	128	128	128	128	128	128	128
2'_c	**26**	2'	32	128	128	128	128	128	128	128	128
2'_d	**27**	2'	16	128	128	128	128	128	128	128	128
2'_e	**28**	2'	8	128	128	128	64	128	64	128	128
3'_a	**29**	3'	32	128	128	128	128	128	128	128	128
3'_b	**30**	3'	8	128	128	128	128	128	128	128	128
3'_c	**31**	3'	32	128	128	128	128	128	128	128	128
3'_d	**32**	3'	64	128	128	128	128	128	128	128	128
3'_e	**33**	3'	16	128	128	128	128	128	128	128	128
4''-aminopropyl-AZI	**34**	core	2	16	128	128	16	128	64	128	32
4''_b	**35**	4''	0.25	4	4	128	0.125	128	1	128	64
4''_c	**36**	4''	0.125	8	8	128	0.125	128	1	128	64
4''_d	**37**	4''	0.25	4	8	128	0.25	128	1	128	64
4''_e	**38**	4''	0.5	4	4	128	0.25	128	1	128	32
4''_f	**39**	4''	4	8	16	16	64	128	4	128	64
4''_g	**40**	4''	4	8	16	64	8	64	2	128	128
4''_h	**41**	4''	1	4	8	128	2	32	2	128	128
4''_i	**42**	4''	4	4	16	32	32	32	4	128	128
4''_j	**43**	4''	0.5	8	32	128	2	128	2	128	128
4''_k	**44**	4''	0.5	8	16	128	1	128	4	128	64
4''_l	**45**	4''	0.5	4	8	128	2	64	1	128	64
4''_o	**46**	4''	2	32	64	128	64	128	16	128	128
4''_1	**47**	4''	2	4	16	128	8	64	2	128	64
4''_9	**48**	4''	2	4	16	64	8	32	2	64	64
4''_H6	**49**	4''	0.125	2	128	128	8	128			4
4''_H12	**50**	4''	0.125	2	128	128	8	128			8
4''_H7	**51**	4''	0.125	2	128	128	8	128			8
4''_4abaR4	**52**	4''	0.25	4	128	128	16	128			16
4''_3FS6	**53**	4''	0.25	4	128	128	16	128			16
3-aminopropyl-AZI	**54**	core	32	128	128	128	64	128	128	128	64

(cc) BY

ID_1	ID_2	Class	S_pyog_B0542	S_aureus_29213	S_aureus_B0331	S_aureus_B0330	S_pneum_B0326	S_pneum_B0633	E_faecalis_29212	S_cerevisiae_7752	E_coli_25922
			MIC, µg mL^{-1}								
3_a	**55**	3	0.25	128	128	128	0.125	128	0.125	2	128
3_b	**56**	3	1	32	128	128	2	128	8	128	128
3_c	**57**	3	1	16	32	128	1	128	8	128	128
3_d	**58**	3	1	32	64	128	2	128	8	128	128
3_e	**59**	3	1	32	128	128	1	128	8	128	64
3_5	**60**	3	32	128	128	128	64	128	128	128	128
3_16	**61**	3	8	64	128	128	32	128	128	128	128
3_10	**62**	3	32	128	128	128	128	128	128	128	128
3_1	**63**	3	4	8	64	128	16	128	16	128	128
3_9	**64**	3	16.9	46.2	43.6	53.3	67.6	106.7	83.6	64	128
3_6	**65**	3	8	32	64	128	32	128	32	128	128
3_8	**66**	3	9.9	20.2	27.1	42.4	28.8	83.0	41.3	120.9	128
3_12	**67**	3	4	16	64	128	16	128	32	128	128
3_7	**68**	3	1	16	32	32	8	64	16	64	64
M4''_NiP12	**69**	M4''	64	128	128	128	128	128	64	128	128
M4''_NiPS	**70**	M4''	1	16	128	128	128	128	16	128	64
M4''_NiP7	**71**	M4''	128	128	128	128	128	128	16	128	128
M4''_NiP16	**72**	M4''	2	32	128	128	128	128	128	128	128
M4''_NiH6	**73**	M4''	2	16	128	128	128	128			64
M4''_Ni_4abaR4	**74**	M4''	0.25	8	128	128	32	128			32
M4''_Ni3FS6	**75**	M4''	128	128	128	128	128	128			128
M4''_NiP6	**76**	M4''	0.125	2	128	128	16	128	8	128	8
M9a_1_Cu	**77**	M9a	0.5	8	128	128	64	128	64	128	32
M9a_2_Cu	**78**	M9a	0.25	8	128	128	32	128	32	128	16
M9a_3_Cu	**79**	M9a	1	16	128	128	64	128	64	128	32
M9a_1_Ni	**80**	M9a	0.5	8	32	32	32	128	32	128	32
M9a_2_Ni	**81**	M9a	0.5	8	64	64	32	128	16	64	128
M9a_3_Ni	**82**	M9a	0.5	16	32	32	32	128	32	128	32

Table S3. ^{1}H NMR chemical shift assignments of the studied compounds

Comp.	Structure	^{1}H NMR [δ/ppm]
9a-aminopropyl- AZI **(2)**	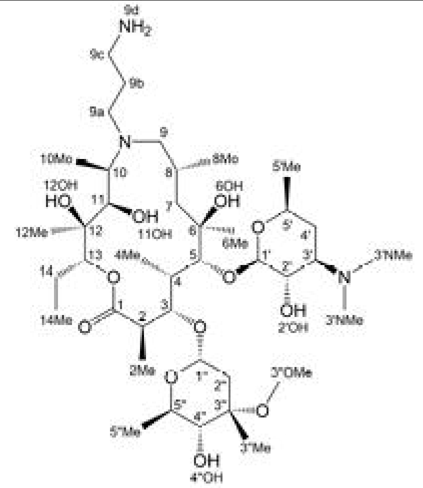	2.98 (1H, H-2), 1.19 (3H, H-2Me), 3.85 (1H, H-3), 2.10 (1H, H-4), 1.00 (3H, H-4Me), 3.50 (1H, H-5), 1.19 (3H, H-6Me), 1.58; 1.43 (2H, H-7), 1.93 (1H, H-8), 0.89 (3H, H-8Me), 2.12; 2.65 (2H, H-9), 2.55; 2.28 (2H, H-9a), 1.67; 1.49 (2H, H-9b), 2.68 (2H, H-9c), 2.93 (1H, H-10), 1.07 (3H, H-10Me), 3.59 (1H, H-11), 1.10 (3H, H-12Me), 4.88 (1H, H13), 1.73; 1.49 (2H, H-14), 0.80 (3H, H-14Me), 4.43 (1H, H-1'), 3.34 (1H, H-2'), 2.68 (1H, H-3'), 2.23 (6H, H-3'NMe$_2$), 1.80; 1.26 (2H, H-4'), 3.69 (1H, H-5'), 1.16 (3H, H-5'Me), 4.92 (1H, H-1''), 2.41; 1.56 (2H, H-2''), 1.19 (3H, H-3''Me), 3.25 (3H, H-3''OMe), 3.15 (1H, H-4''), 4.12 (1H, H-5''), 1.25 (3H, H-5''Me); purity: 96.5 %
9a_f **(8)**	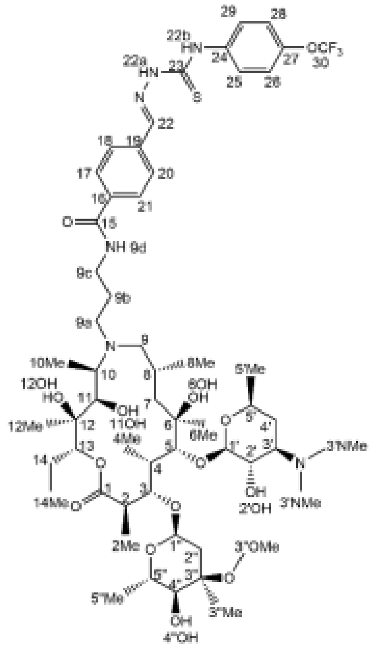	2.84 (1H, H-2), 1.18 (3H, H-2Me), 4.09 (1H, H-3), 2.05 (1H, H-4), 1.02 (3H, H-4Me), 3.58 (1H, H-5), 1.26 (3H, H-6Me), 1.58; 1.45 (2H, H-7), 1.99 (1H, H-8), 0.92 (3H, H-8Me), 2.20; 2.73 (2H, H-9), 3.05; 2.65 (2H, H-9a), 1.76; 1.89 (2H, H-9b), 3.35 (2H, H-9c), 7.61 (1H, H-9d), 2.84 (1H, H-10), 1.11 (3H, H-10Me), 3.65 (1H, H-11), 1.07 (3H, H-12Me), 4.80 (1H, H13), 1.83; 1.46 (2H, H-14), 0.86 (3H, H-14Me), 7.91 (2H, H-17,21), 7.87 (2H, H-18,20), 8.09 (1H, H-22), 7.44 (2H, H-25, 29), 7.33 (2H, H-26,28), 4.45 (1H, H-1'), 3.10 (1H, H-2'), 2.54(1H, H-3'), 2.27 (6H, H-3'NMe$_2$), 1.71; 1.14 (2H, H-4'), 3.59 (1H, H-5'), 1.14 (3H, H-5'Me), 4.96 (1H, H-1''), 2.38; 1.59 (2H, H-2''), 1.21 (3H, H-3''Me), 3.31 (3H, H-3''OMe), 2.98 (1H, H-4''), 4.09 (1H, H-5''), 1.24 (3H, H-5''Me); purity: 99.9 %
9a_g **(9)**	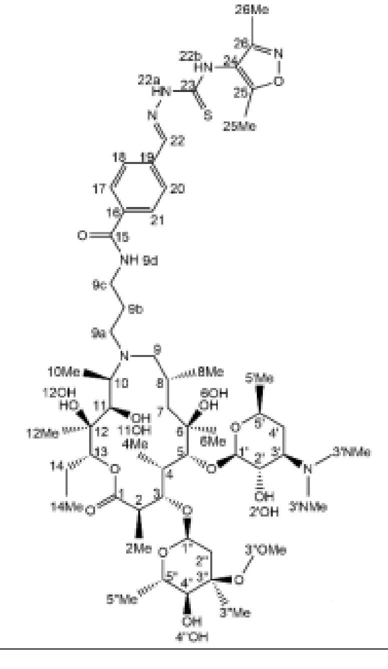	2.82 (1H, H-2), 1.19 (3H, H-2Me), 4.11 (1H, H-3), 2.04 (1H, H-4), 1.04 (3H, H-4Me), 3.59 (1H, H-5), 1.24 (3H, H-6Me), 1.54; 1.47 (2H, H-7), 1.99 (1H, H-8), 0.92 (3H, H-8Me), 2.21; 2.69 (2H, H-9), 3.05; 2.64 (2H, H-9a), 1.88; 1.75 (2H, H-9b), 3.36 (2H, H-9c), 7.36 (1H, 9d), 2.84 (1H, H-10), 1.11 (3H, H-10Me), 3.67 (1H, H-11), 1.08 (3H, H-12Me), 4.82 (1H, H-13), 1.82; 1.48 (2H, H-14), 0.87 (3H, H-14Me), 7.90 (2H, H-17,21), 7.86 (2H, H-18,20), 8.09 (1H, H-22), 2.31 (3H, H-25Me), 2.19 (3H, H-26Me), 4.46 (1H, H-1'), 3.09 (1H, H-2'), 2.51 (1H, H3'), 2.27 (6H, H-3'NMe$_2$), 1.70; 1.13 (2H, H-4'), 3.59 (1H, H-5'), 1.14 (3H, H-5'Me), 4.92 (1H, H-1''), 2.38; 1.58 (2H, H-2''), 1.21 (3H, H-3''Me), 3.31 (3H, H-3''OMe), 2.96 (1H, H-4''), 4.07(1H, H-5''), 1.23 (3H, H-5''Me); purity: 95.8 %

CC BY

Comp.	Structure	^{1}H NMR [*δ*/ppm]
9a_h (**10**)	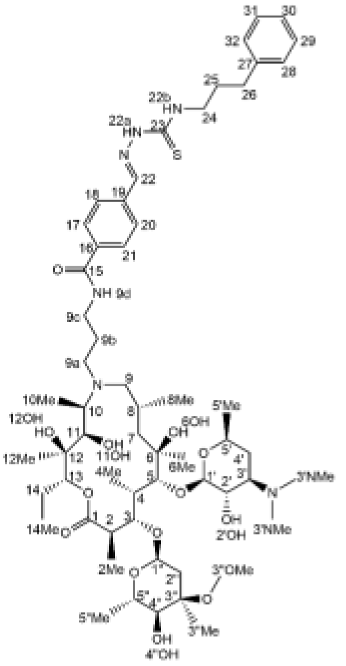	2.79 (1H, H-2), 1.15 (3H, H-2Me), 4.06 (1H, H-3), 2.01 (1H, H-4), 1.00 (3H, H-4 Me), 3.55 (1H, H-5), 1.22 (3H, H-6Me), 1.52; 1.43 (2H, H-7), 1.97 (1H, H-8), 0.89 (3H, H-8Me), 2.21; 2.70 (2H, H-9), 3.03; 2.62 (2H, H-9a), 1.87;1.93 (2H, H-9b), 3.32 (2H, H-9c), 7.52 (1H, H-9d), 2.83 (1H, H-10), 1.09 (3H, H-10Me), 3.64 (1H, H-11), 1.04 (3H, H-12Me), 4.78 (1H, H13), 1.79; 1.43 (2H, H-14), 0.83 (3H, H-14Me), 7.82 (2H, H-17,21), 7.78 (2H, H-18,20), 7.95 (1H, H-22), 8.65 (1H, H-22b), 3.66 (2H, H-24), 1.95 (2H, H-25), 2.67 (2H, H-26), 7.22 (2H, H-28,32), 7.27 (2H, H-29,31), 7.16 (1H, H-30), 4.43 (1H, H-1'), 3.06 (1H, H-2'), 2.50 (1H, H3'), 2.26 (6H, H-3'NMe_2), 1.67; 1.10 (2H, H-4'), 3.56 (1H, H-5'), 1.11 (3H, H-5'Me), 4.92 (1H, H-1"), 2.34; 1.54 (2H, H-2"), 1.18 (3H, H-3"Me), 3.27 (3H, H-3"OMe), 2.94 (1H, H-4"), 4.05 (1H, H-5"), 1.21 (3H, H-5"Me); purity: 95.4 %
9a_i (**11**)	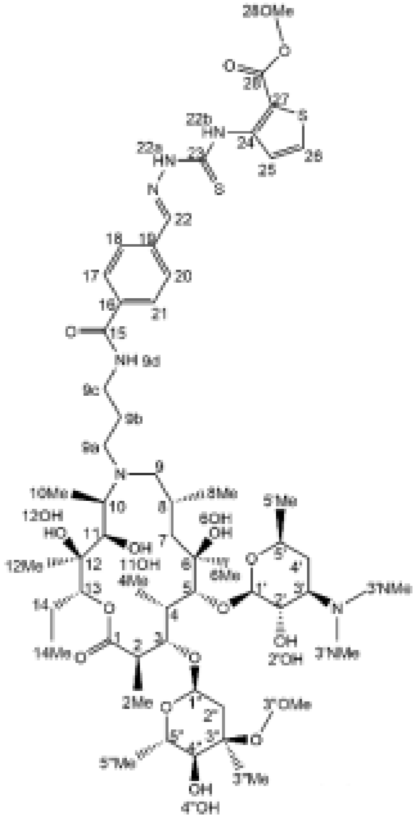	2.83 (1H, H-2), 1.19 (3H, H-2Me), 4.11 (1H, H-3), 2.05 (1H, H-4), 1.03 (3H, H-4Me), 3.59 (1H, H-5), 1.24 (3H, H-6Me), 1.55; 1.48 (2H, H-7), 2.01 (1H, H-8), 0.92 (3H, H-8Me), 2.23; 2.74 (2H, H-9), 3.07; 2.68 (2H, H-9a), 1.91; 1.78 (2H, H-9b), 3.37 (2H, H-9c), 2.86 (1H, H10), 1.12 (3H, H-10Me), 3.67 (1H, H-11), 1.07 (3H, H-12Me), 4.81 (1H, H-13), 1.83; 1.47(2H, H-14), 0.86 (3H, H-14Me), 7.90 (2H, H-17,21), 8.03 (2H, H-18,20), 8.10 (1H, H-22), 8.96 (1H, H-25), 7.69 (1H, H-26), 3.97 (3H, H-28OMe), 4.46 (1H, H-1'), 3.09 (1H, H-2'), 2.53 (1H, H-3'), 2.28 (6H, H-3'NMe_2), 1.69; 1.10 (2H, H-4'), 3.58 (1H, H-5'), 1.14 (3H, H-5'Me), 4.95 (1H, H-1"), 2.39; 1.58 (2H, H-2"), 1.20 (3H, H-3"Me), 3.30 (3H, H-3"OMe), 2.97 (1H, H-4"), 4.09 (1H, H-5"), 1.24 (3H, H-5"Me); purity: 95.7 % δ

Comp.	Structure	^{1}H NMR [δ/ppm]
9a_m (**12**)	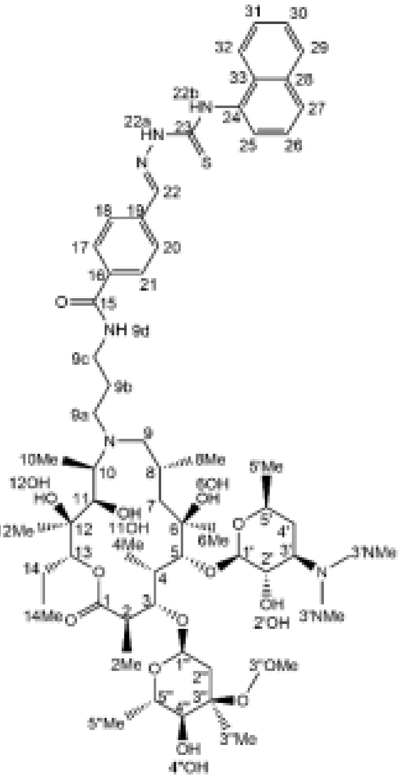	2.80 (1H, H-2), 1.19 (3H, H-2Me), 4.10 (1H, H-3), 2.02 (1H, H-4), 1.04 (3H, H-4Me), 3.58 (1H, H-5), 1.24 (3H, H-6Me), 1.53; 1.46 (2H, H-7), 1.97 (1H, H-8), 0.90 (3H, H-8Me), 2.18; 2.65 (2H, H-9), 3.03; 2.61 (2H, H-9a), 1.85;1.73 (2H, H-9b), 3.35 (2H, H-9c), 7.46 (1H, 9d), 2.80 (1H, H-10), 1.10 (3H, H-10Me), 3.66 (1H, H-11), 1.07 (3H, H-12Me), 4.82 (1H, H-13), 1.81; 1.47 (2H, H-14), 0.87 (3H, H-14Me), 7.64 (2H, H-17,21), 7.99 (2H, H-18,20), 8.15 (1H, H-22), 8.38 (1H, H-22b), 7.56 (4H, H-25, 26, 30, 31), 7.95 (3H, H-27,29,32), 4.45 (1H, H-1'), 3.09 (1H, H-2'), 2.50 (1H, H-3'), 2.28 (6H, H-3'NMe_2), 1.68; 1.10 (2H, H-4'), 3.58 (1H, H-5'), 1.12 (3H, H-5'Me), 4.92 (1H, H-1"), 2.34; 1.54 (2H, H-2"), 1.20 (3H, H-3"Me), 3.30 (3H, H-3"OMe), 2.95 (1H, H-4"), 4.07 (1H, H-5"), 1.22 (3H, H-5"Me); purity: 97.8 %
9a_n (**13**)	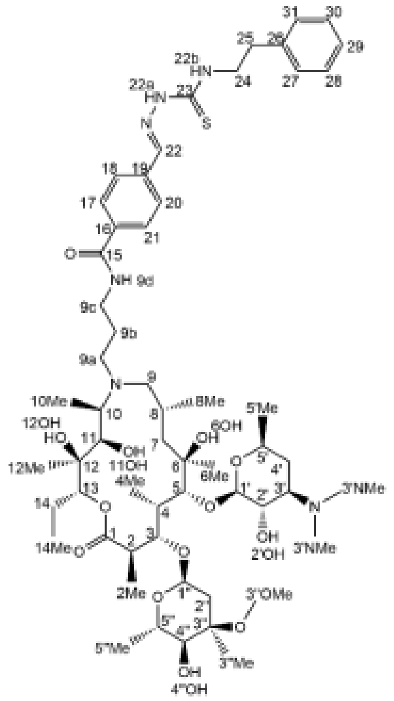	2.84 (1H, H-2), 1.19 (3H, H-2Me), 4.10 (1H, H-3), 2.05 (1H, H-4), 1.03 (3H, H-4Me), 3.59 (1H, H-5), 1.25 (3H, H-6Me), 1,58; 1.47 (2H, H-7), 1.99 (1H, H-8), 0.92 (3H, H-8Me), 2.22; 2.73 (2H, H-9), 3.05; 2.65 (2H, H-9a), 1.90;1.77 (2H, H-9b), 3.36 (2H, H-9c), 7.50 (1H, 9d), 2.85 (1H, H-10), 1.11 (3H, H-10Me), 3.67 (1H, H-11), 1.07 (3H, H-12Me), 4.80 (1H, H-13), 1.84; 1.47 (2H, H-14), 0.87 (3H, H-14Me), 7.85 (2H, H-17,21), 7.74 (2H, H-18,20), 7.96 (1H, H-22), 3.89 (2H, H-24), 2.99 (2H, H-25), 7.36 (2H, H-27,31), 7.35 (2H, H-28,30), 7.28 (1H, H-29), 4.46 (1H, H-1'), 3.07 (1H, H-2'), 2.52 (1H, H-3'), 2.27 (6H, H-3'NMe_2), 1.70; 1.11 (2H, H-4'), 3.59 (1H, H-5'), 1.14 (3H, H-5'Me), 4.95 (1H, H-1"), 2.38; 1.59 (2H, H-2"), 1.21 (3H, H-3"Me), 3.31 (3H, H-3"OMe), 2.98 (1H, H-4"), 4.09 (1H, H-5"), 1.25 (3H, H-5"Me); purity: 99.8 %

cc BY

Comp.	Structure	^{1}H NMR [δ/ppm]
9a_o (**14**)	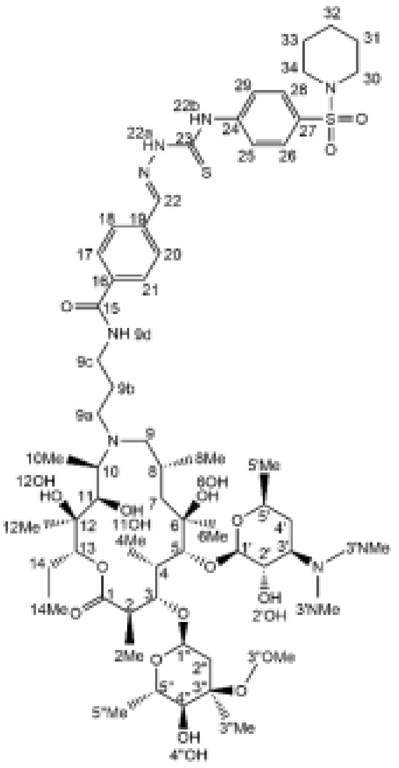	2.80 (1H, H-2), 1.16 (3H, H-2Me), 4.05 (1H, H-3), 2.01 (1H, H-4), 1.01 (3H, H-4Me), 3.56 (1H, H-5), 1.23 (3H, H-6Me), 1.54; 1.46 (2H, H-7), 2.00 (1H, H-8), 0.91 (3H, H-8Me), 2.25; 2.71 (2H, H-9), 1.88; 1.74 (2H, H-9b), 3.34 (2H, H-9c), 7.37 (1H, H-9d), 2.90 (1H, H-10), 1.12 (3H, H-10Me), 3.65 (1H, H-11), 1.06 (3H, H-12Me), 4.79 (1H, H-13), 1.79; 1.44 (2H, H-14), 0.84 (3H, H-14Me), 7.85 (2H, H-17,21), 7.90 (2H, H-18,20), 8.08 (1H, H-22), 8.01 (2H, H-25,29), 7.73 (2H, H-26,28), 2.96 (2H, H-30,34), 1.60 (2H, H-31,33), 1.40 (2H, H-32), 4.44 (1H, H-1'), 3.09 (1H, H-2'), 2.57 (1H, H-3'), 2.30 (6H, H-3'NMe_2), 1.70; 1,13 (2H, H-4'), 3.57 (1H, H-5'), 1.13 (3H, H-5'Me), 4.89 (1H, H-1"), 2.35; 1.55 (2H, H-2"), 1.18 (3H, H-3"Me), 3.28 (3H, H-3"OMe), 2.94 (1H, H-4"), 4.04 (1H, H-5"), 1.21 (3H, H-5"Me); purity: 96.4 %
9a_p (**15**)	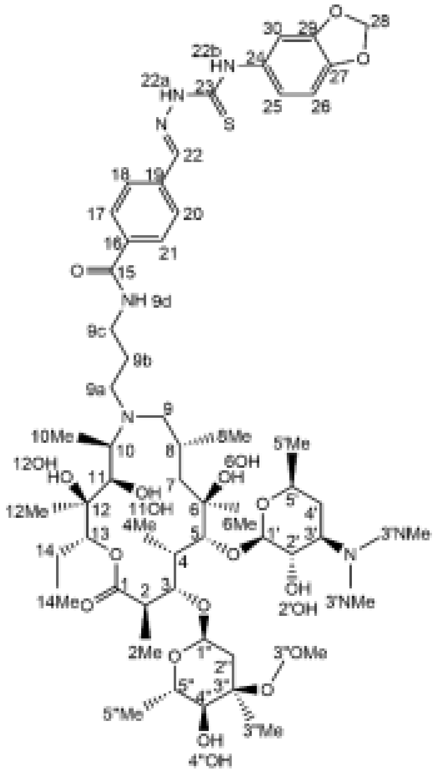	2.80 (1H, H-2), 1.17 (3H, H-2Me), 4.06 (1H, H-3), 2.01 (1H, H-4), 1.01 (3H, H-4Me), 3.55 (1H, H-5), 1.23 (3H, H-6Me), 1.53; 1.45 (2H, H-7), 1.98 (1H, H-8), 0.91 (3H, H-8Me), 2.22; 2.71 (2H, H-9), 3.06; 2.64 (2H, H-9a),1.87; 1.74 (2H, H-9b), 3.34 (2H, H-9c), 7.36 (1H, H-9d), 2.86 (1H, H-10), 1.10 (3H, H-10Me), 3.64 (1H, H-11), 1.05 (3H, H-12 Me), 4.78 (1H, H13), 1.79; 1.43 (2H, H-14), 0.84 (3H, H-14Me), 7.82 (2H, H-17,21), 7.86 (2H, H-18,20), 8.03(1H, H-22), 9.35 (1H, H-22b), 6.92 (1H, H-25), 6.83 (2H, H-26), 5.99 (1H, H-28), 7.15 (2H, H-30), 4.43 (1H, H-1'), 3.07 (1H, H-2'), 2.54 (1H, H-3'), 2.28 (6H, H-3'NMe_2), 1.69; 1.11 (2H, H-4'), 3.57 (1H, H-5'), 1.12 (3H, H-5'Me), 4.89 (1H, H-1"), 2.34; 1.54 (2H, H-2"), 1.18 (3H, H-3"Me), 3.28 (3H, H-3"OMe), 2.94 (1H, H-4"), 4.04 (1H, H-5"), 1.21 (3H, H-5"Me); purity: 95.2 %

Comp.	Structure	^{1}H NMR [*δ*/ppm]
9a_s **(16)**	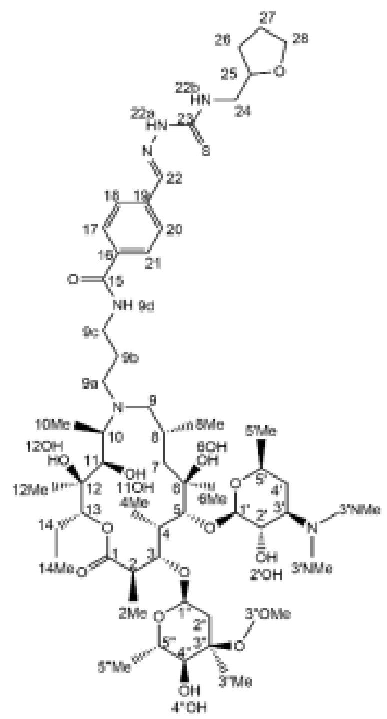	2.79 (1H, H-2), 1.16 (3H, H-2Me), 4.01 (1H, H-3), 2.01 (1H, H-4), 1.02 (3H, H-4Me), 3.54 (1H, H-5), 1.28 (3H, H-6Me), 2.11 (1H, H-8), 0.96 (3H, H-8Me), 2.49; 2.90 (2H, H-9), 2.87 (2H, H-9a), 2.03 (2H, H-9b), 3.40 (2H, H-9c), 7.96 (1H, H-9 d), 2.94 (1H, H-10), 1.24 (3H, H-10Me), 3.73 (1H, H-11), 1.08 (3H, H-12Me), 4.89 (1H, H-13), 1.83; 1.45 (2H, H-14), 0.81(3H, H-14Me), 7.86 (2H, H-17,21), 7.72 (2H, H-18,20), 8.02 (1H, H-22), 3.82; 3.66 (2H, H24), 4.13 (1H, H-25), 1.85; 1.73 (2H, H-26), 1.89 (2H, H-27), 3.67; 3.60 (2H, H-28), 4.49 (1H, H-1'), 3.29 (1H, H-2'), 2.92 (1H, H-3'), 2.52 (6H, H-3'NMe_2), 1.82; 1.24 (2H, H-4'), 3.57 (1H, H-5'), 1.13 (3H, H-5'Me), 4.88 (1H, H-1''), 2.35; 1.57 (2H, H-2''), 1.20 (3H, H-3''Me), 3.29(3H, H-3''OMe), 2.98 (1H, H-4''), 4.05 (1H, H-5''), 1.22 (3H, H-5''Me); purity: 97.9 %
9a_6 **(17)**	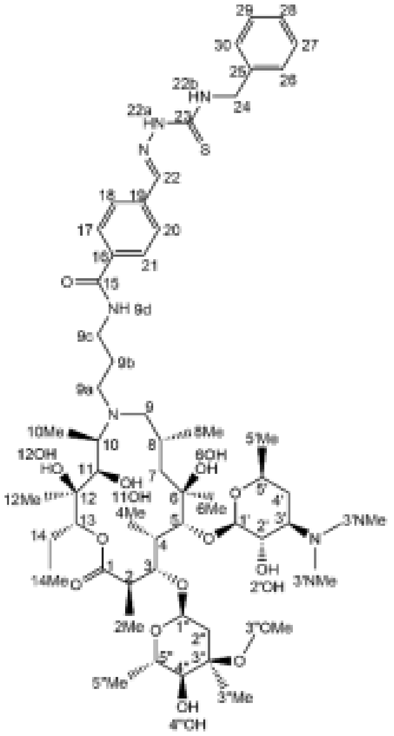	2.81 (1H, H-2), 1.19 (3H, H-2Me), 4.10 (1H, H-3), 2.03 (1H, H-4), 1.04 (3H, H-4Me), 3.58 (1H, H-5), 1.24 (3H, H-6Me), 1.53; 1.46 (2H, H-7), 1.97 (1H, H-8), 0.90 (3H, H-8Me), 2.16; 2.64 (2H, H-9), 3.03; 2.60 (2H, H-9a), 1.85; 1.73 (2H, H-9b), 3.34 (2H, H-9c), 7.36 (1H, 9d), 2.81 (1H, H-10), 1.09 (3H, H-10Me), 3.66 (1H, H-11), 1.07 (3H, H-12Me), 4.83 (1H, H-13), 1.81; 1.47 (2H, H-14), 0.87 (3H, H-14Me), 7.83 (2H, H-17,21), 7.81 (2H, H-18,20), 8.01 (1H, H-22), 8.44 (1H, H-22b), 4.92 (1H, H-24), 7.39 (2H, H-26,30), 7.35 (2H, H-27,29), 7.26 (1H, H-28), 4.46 (1H, H-1'), 3.09 (1H, H-2'), 2.51 (1H, H-3'), 2.27 (6H, H-3'NMe_2), 1.70; 1.13 (2H, H-4'), 3.59 (1H, H-5'), 1.14 (3H, H-5'Me), 4.92 (1H, H-1''), 2.38; 1.58 (2H, H-2''), 1.21 (3H, H-3''Me), 3.31 (3H, H-3''OMe), 2.96 (1H, H-4''), 4.07 (1H, H-5''), 1.23 (3H, H-5''Me); purity: 95.1 %

(cc) BY

Comp.	Structure	^{1}H NMR [*δ*/ppm]
S4_1 (**18**)	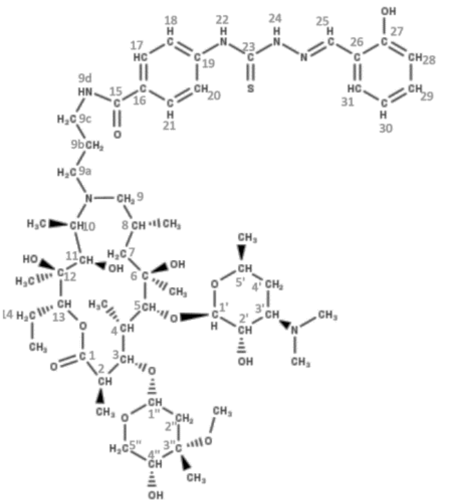	2.98 (1H, H-2), 0.96 (3H, H-2Me), 4.32 (1H, H-3), 1.37 (1H, H-4), 1.02 (3H, H-4Me), 4.49 (1H, H-5), 1.17 (3H, H-6Me), 2.98 (2H, H-7), 1.97 (1H, H-8), 0.87 (3H, H-8Me), 2.94 (2H, H-9), 2.61; 1.85 (2H, H-9a), 1.79 (2H, H-9b), 1.84 (2H, H-9c), 6.80 (1H, H-9d), 3.59 (1H, H-10), 1.21 (3H, H-10Me), 4.08 (1H, H-11), 1.04 (3H, H-12Me), 4.93 (1H, H-13), 1.53-2.30 (2H, H-14), 1.26 (3H, H-14Me), 7.90 (1H, H-17; H-21), 7.61 (1H, H-18; H-20), 8.17 (1H, H-29), 7.34 (1H, H-30), 7.13 (1H, H-31), 4.53 (1H, H-1'), 3.08 (1H, H-2'), 2.40 (1H, H-3'), 2.88 (6H, H-3'NMe_2), 1.85 (2H, H-40), 3.58 (1H, H-5'), 1.09 (3H, H-5'Me), 1.80 (2H, H-2''), 1.47 (3H, H-3''Me), 3.12 (1H, H-3''OMe), 3.00 (1H, H-4''), 4.06 (1H, H-5''), 1.14 (3H, H-5''Me); purity: 96.5 %
S4_2 (**19**)	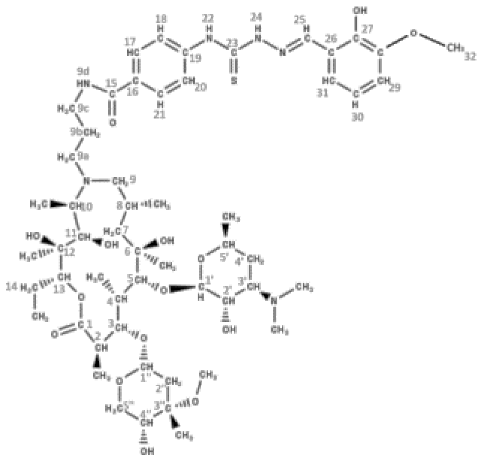	2.95 (1H, H-2), 0.93 (3H, H-2Me), 4.33 (1H, H-3), 1.37 (1H, H-4), 1.02 (3H, H-4Me), 4.49 (1H, H-5), 1.17 (3H, H-6Me), 2.98 (2H, H-7), 1.94 (1H, H-8), 0.87 (3H, H-8Me), 2.92 (2H, H-9), 1.85 (2H, H-9a), 1.79 (2H, H-9b), 1.84 (2H, H-9c), 6.80 (1H, H-9d), 3.59 (1H, H-10), 1.21 (3H, H-10Me), 4.10 (1H, H-11), 1.04 (3H, H-12Me), 4.93 (1H, H-13), 1.53–2.30 (2H, H-14), 1.26 (3H, H-14Me), 7.90 (1H, H-17; H-21), 7.61 (1H, H-18; H-20), 8.17 (1H, H-29), 7.34 (1H, H-30), 7.13 (1H, H-31), 3.32 (3H, H-32), 4.53 (1H, H-1'), 3.08 (1H, H-2'), 2.40 (1H, H-3'), 2.88 (6H, H-3'NMe_2), 1.85 (2H, H-40), 3.58 (1H, H-5'), 1.09 (3H, H-5'Me), 1.80 (2H, H-2''), 1.47 (3H, H-3''Me), 3.12 (1H, H-3''OMe), 3.00 (1H, H-4''), 4.06 (1H, H-5''), 1.14 (3H, H-5''Me); purity: 97.2 %
S4_3 (**20**)	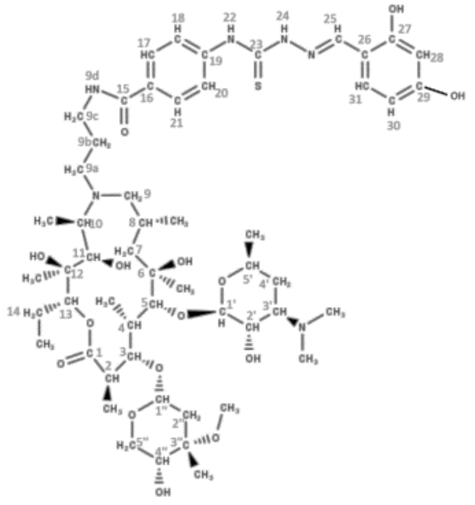	2.99 (1H, H-2), 0.96 (3H, H-2Me), 4.31 (1H, H-3), 1.35 (1H, H-4), 1.02 (3H, H-4Me), 4.49 (1H, H-5), 1.17 (3H, H-6Me), 2.98 (2H, H-7), 1.97 (1H, H-8), 0.87 (3H, H-8Me), 2.94 (2H, H-9), 1.88 (2H, H-9a), 1.79 (2H, H-9b), 1.84 (2H, H-9c), 6.80 (1H, H-9d), 3.59 (1H, H-10), 1.21 (3H, H-10Me), 4.08 (1H, H-11), 1.04 (3H, H-12Me), 4.93 (1H, H-13), 1.53–2.30 (2H, H-14), 1.26 (3H, H-14Me), 7.90 (1H, H-17; H-21), 7.61 (1H, H-18; H-20), 8.17 (1H, H-29), 2.75 (1H, H-29O), 7.34 (1H, H-30), 7.13 (1H, H-31), 4.53 (1H, H-1'), 3.08 (1H, H-2'), 2.40 (1H, H-3'), 2.88 (6H, H-3'NMe_2), 1.85 (2H, H-4'), 3.58 (1H, H-5'), 1.09 (3H, H-5'Me), 1.80 (2H, H-2''), 1.47 (3H, H-3''Me), 3.1 (1H, H-3''OMe), 3.01 (1H, H-4''), 4.06 (1H, H-5''), 1.14 (3H, H-5''Me); purity: 96.3 %

Comp.	Structure	^{1}H NMR [δ / ppm]
9a-2 **(21)**	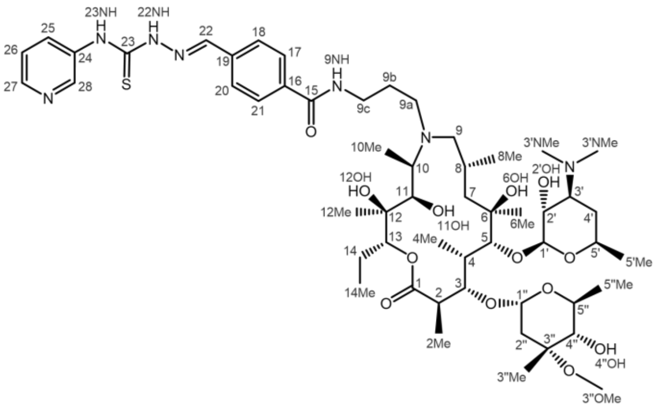	2.89 (1H, H-2), 1.18 (3H, H-2Me), 4.09 (1H, H-3), 2.05 (1H, H-4), 1.07 (3H, H-4Me), 3.61 (1H, H-5), 1.35 (3H, H-6Me), 1.58; 1.39 (2H, H-7), 1.99 (1H, H-8), 0.99 (3H, H-8Me), 2.35 (2H, H-9), 3.05; 2.65 (2H, H-9a), 1.76; 1.89 (2H, H-9b), 3.31 (2H, H-9c), 7.61 (1H, H-9d), 2.99 (1H, H-10), 1.21 (3H, H-10Me), 3.71 (1H, H-11), 1.25 (3H, H-12Me), 4.66 (1H, H13), 1.69 (2H, H-14), 0.86 (3H, H-14Me), 7.82 (2H, H-17,21), 7.69 (2H, H-18,20), 7.87 (1H, H-22), 7.72 (1H, H-25), 7.38 (1H, H-26), 8.12 (1H, H-27), 8.36 (1H, H-28), 4.42 (1H, H-1'), 3.40 (1H, H-2'), 2.54(1H, H-3'), 3.33 (6H, H-3'NMe$_2$), 1.69 (2H, H-4'), 3.58 (1H, H-5'), 1.14 (3H, H-5'Me), 5.02 (1H, H-1"), 2.33 (2H, H-2"), 1.34 (3H, H-3"Me), 3.31 (3H, H-3"OMe), 3.22 (1H, H-4"), 4.18 (1H, H-5"), 1.37 (3H, H-5"Me); purity: 98.5 %
9a-4 **(22)**	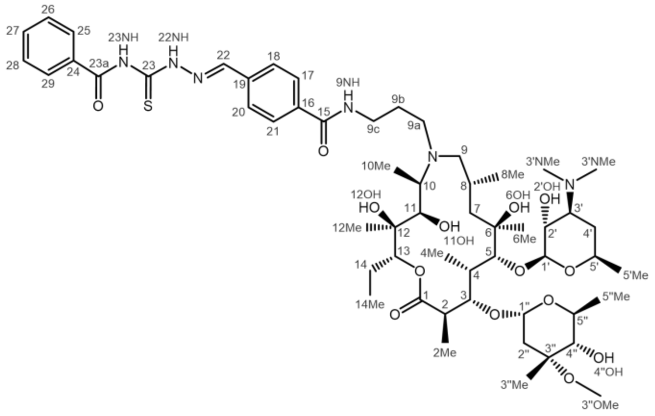	2.88 (1H, H-2), 1.25 (3H, H-2Me), 4.11 (1H, H-3), 2.05 (1H, H-4), 1.08 (3H, H-4Me), 3.62 (1H, H-5), 1.38 (3H, H-6Me), 1.58; 1.42 (2H, H-7), 1.99 (1H, H-8), 0.99 (3H, H-8Me), 2.36 (2H, H-9), 3.05; 2.65 (2H, H-9a), 1.76; 1.89 (2H, H-9b), 3.30 (2H, H-9c), 7.61 (1H, H-9d), 3.03 (1H, H-10), 1.19 (3H, H-10Me), 3.71 (1H, H-11), 1.21 (3H, H-12Me), 4.67 (1H, H13), 1.80 (2H, H-14), 0.87 (3H, H-14Me), 7.93 (2H, H-17,21), 7.84 (2H, H-18,20), 7.92 (1H, H-22), 7.99, 8.01 (2H, H-25, 29), 7.64 (1H, H-26), 7.52 (1H, H-27), 7.52 (1H, H-28), 4.45 (1H, H-1'), 3.41 (1H, H-2'), 2.54(1H, H-3'), 2.33 (6H, H-3'NMe$_2$), 1.83 (2H, H-4'), 3.60 (1H, H-5'), 1.14 (3H, H-5'Me), 5.01 (1H, H-1"), 2.33 (2H, H-2"), 1.36 (3H, H-3"Me), 3.33 (3H, H-3"OMe), 3.23 (1H, H-4"), 4.19 (1H, H-5"), 1.40 (3H, H-5"Me); purity: 98.0 %
9a-13 **(23)**	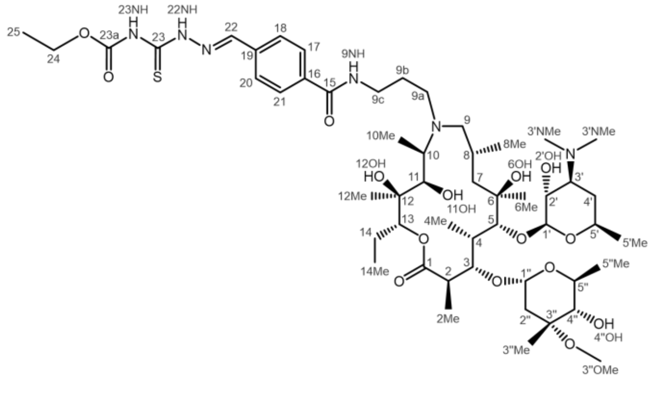	2.87 (1H, H-2), 1.28 (3H, H-2Me), 4.11 (1H, H-3), 2.05 (1H, H-4), 1.08 (3H, H-4Me), 3.64 (1H, H-5), 1.36 (3H, H-6Me), 1.44; 1.42 (2H, H-7), 1.99 (1H, H-8), 0.99 (3H, H-8Me), 2.36; 2.34 (2H, H-9), 3.05; 2.65 (2H, H-9a), 1.76; 1.89 (2H, H-9b), 3.35 (2H, H-9c), 7.61 (1H, H-9d), 3.04 (1H, H-10), 1.2 (3H, H-10Me), 3.71 (1H, H-11), 1.25 (3H, H-12Me), 4.65 (1H, H13), 1.63 (2H, H-14), 0.86 (3H, H-14Me), 7.94 (2H, H-17,21), 7.88 (2H, H-18,20), 8.66 (1H, H-22), 1.21 (3H, H-26) 4.45 (1H, H-1'), 3.41 (1H, H-2'), 2.54 (1H, H-3'), 2.3 (6H, H-3'NMe$_2$), 1.71; 1.63 (2H, H-4'), 3.61 (1H, H-5'), 1.14 (3H, H-5'Me), 5.01 (1H, H-1"), 2.34; 2.33 (2H, H-2"), 1.35 (3H, H-3"Me), 3.33 (3H, H-3"OMe), 3.22 (1H, H-4"), 4.09 (1H, H-5"), 1.39 (3H, H-5"Me); purity: 96.1 %

CC BY

Comp.	Structure	^{1}H NMR [*δ* / ppm]
4''-aminopropyl-AZI **(34)**	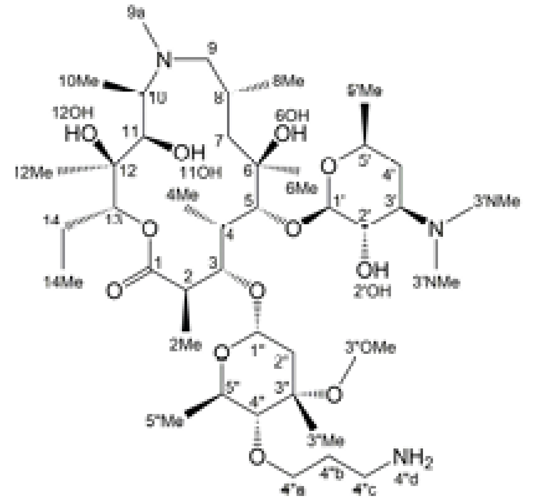	2.89 (1H, H-2), 1.16 (3H, H-2Me), 4.01 (1H, H-3), 2.0 (1H, H-4), 0.93 (3H, H-4Me), 3.50 (1H, H-5), 1.25 (3H, H-6Me), 1.57; 1.39 (2H, H-7), 1.95 (1H, H-8), 0.84 (3H, H-8Me), 2.54; 2.21 (2H, H-9), 2.28 (3H, H-9a), 2.91 (1H, H-10), 1.01 (3H, H-10Me), 3.48 (1H, H-11), 1.08 (3H, H-12Me), 4.80 (1H, H-13), 1.73; 1.48 (2H, H-14), 0.79 (3H, H-14Me), 4.38 (1H, H-1'), 3.31 (1H, H-2'), 2.70 (1H, H-3'), 2.25 (6H, H-3'NMe_2), 1.78; 1.24 (2H, H-4'), 3.66 (1H, H-5'), 1.17 (3H, H-5'Me), 4.93 (1H, H-1''), 2.44; 1.64 (2H, H-2''), 1.20 (3H, H-3''Me), 3.26 (3H, H-3''OMe), 2.97 (1H, H-4''), 3.68 (2H, H-4''a), 1.76 (2H, H-4''b), 2.77 (2H, H-4''c), 4.16 (1H, H-5''), 1.29 (3H, H-5''Me); purity: 97.3 %
4''_b **(35)**	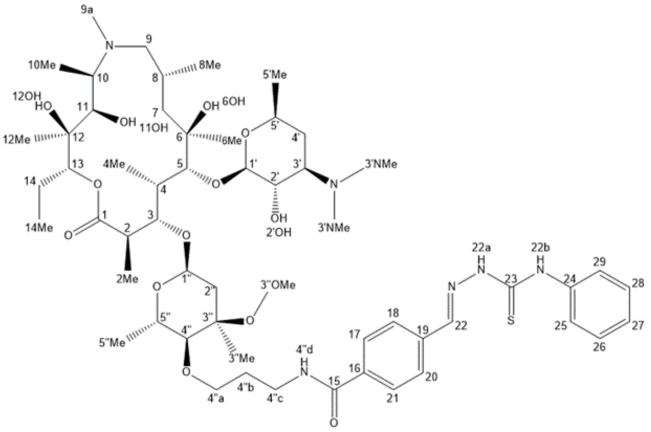	2.77 (1H, H-2), 1.18 (3H, H-2Me), 4.30 (1H, H-3), 1.95 (1H, H-4), 1.05 (3H, H-4Me), 3.61 (1H, H-5), 1.28 (3H, H-6Me), 1.65; 1.36 (2H, H-7), 1.97 (1H, H-8), 0.92 (3H, H-8Me), 2.47; 2.16 (2H, H-9), 2.29 (3H, H-9a), 2.75 (1H, H-10), 1.05 (3H, H-10Me), 3.57 (1H, H-11), 1.06 (3H, H-12Me), 4.75 (1H, H-13), 1.83; 1.47 (2H, H-14), 0.87 (3H, H-14Me), 7.90 (2H, H-17,21), 7.94 (2H, H-18,20), 8.08 (1H, H-22), 7.67 (2H, H-25,29), 7.42 (1H, H-26,28), 7.27 (1H, H-27), 4.52 (1H, H-1'), 3.05 (1H, H-2'), 2.35 (1H, H-3'), 2.11 (6H, H-3'NMe_2), 1.57; 1.07 (2H, H-4'), 3.66 (1H, H-5'), 1.18 (3H, H-5'Me), 4.94 (1H, H-1''), 2.37; 1.57 (2H, H-2''), 1.25 (3H, H-3''Me), 3.23 (3H, H-3''OMe), 2.88 (1H, H-4''), 3.85; 3.74 (2H, H-4''a), 1.87 (2H, H-4''b), 3.38; 3.64 (2H, H-4''c), 7.51 (1H, H-4''d), 4.33 (1H, H-5''), 1.31 (3H, H-5''Me); purity: 97.0 %
4''_c **(36)**	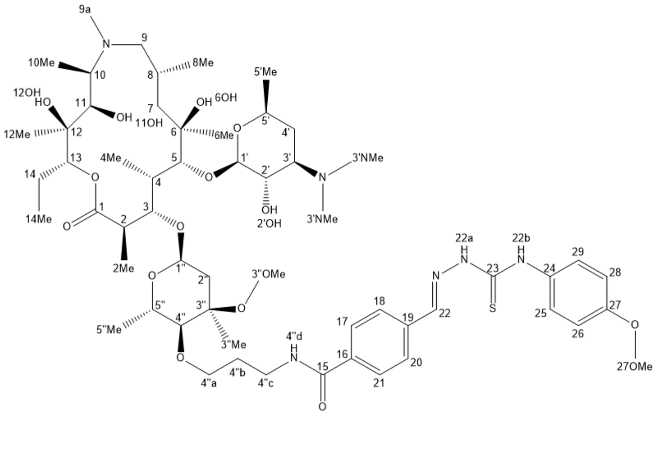	2.79 (1H, H-2), 1.20 (3H, H-2Me), 4.19 (1H, H-3), 1.99 (1H, H-4), 1.03 (3H, H-4Me), 3.59 (1H, H-5), 1.34 (3H, H-6Me), 1.66; 1.51 (2H, H-7), 2.09 (1H, H-8), 0.98 (3H, H-8Me), 2.75; 2.51 (2H, H-9), 2.54 (3H, H-9a), 2.77 (1H, H-10), 1.20 (3H, H-10Me), 3.61 (1H, H-11), 1.10 (3H, H-12Me), 4.78 (1H, H-13), 1.85; 1.48 (2H, H-14), 0.89 (3H, H-14Me), 7.89 (2H, H-17,21), 7.93 (2H, H-18,20), 8.07 (1H, H-22), 7.49 (2H, H-25,29), 6.97 (1H, H-26,28), 3.83 (3H, H-27OMe), 4.57 (1H, H-1'), 3.22 (1H, H-2'), 3.08 (1H, H-3'), 2.42 (6H, H-3'NMe_2), 1.81; 1.23 (2H, H-4'), 3.79 (1H, H-5'), 1.21 (3H, H-5'Me), 4.92 (1H, H-1''), 2.38; 1.58 (2H, H-2''), 1.25 (3H, H-3''Me), 3.30 (3H, H-3''OMe), 2.90 (1H, H-4''), 3.83; 3.74 (2H, H-4''a), 1.88 (2H, H-4''b), 3.45; 3.56 (2H, H-4''c), 7.39 (1H, H-4'' d), 4.27 (1H, H-5''), 1.32 (3H, H-5''Me); purity: 96.2 %

Comp.	Structure	^{1}H NMR [δ / ppm]
4''_d (**37**)	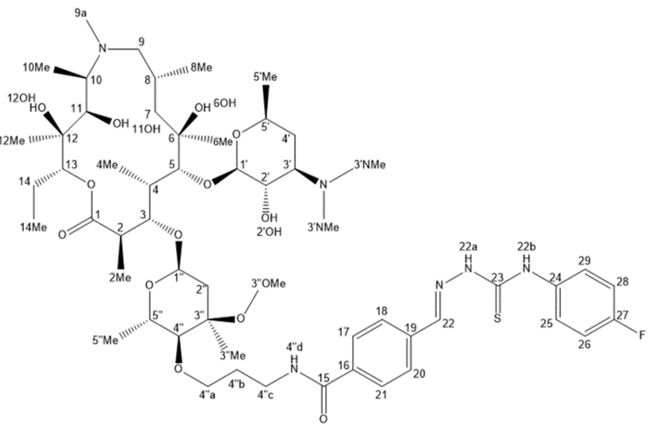	2.75 (1H, H-2), 1.17 (3H, H-2Me), 4.31 (1H, H-3), 1.93 (1H, H-4), 1.03 (3H, H-4Me), 3.60 (1H, H-5), 1.28 (3H, H-6Me), 1.65; 1.34 (2H, H-7), 1.98 (1H, H-8), 0.92 (3H, H-8Me), 2.48; 2.16 (2H, H-9), 2.29 (3H, H-9a) 2.76 (1H, H-10), 1.04 (3H, H-10Me), 3.55 (1H, H-11), 1.05 (3H, H-12Me), 4.75 (1H, H-13), 1.83; 1.47 (2H, H-14), 0.87 (3H, H-14Me), 7.91 (2H, H-17,21), 7.94 (2H, H-18,20), 8.08 (1H, H-22), 7.65 (2H, H-25, 29), 7.14 (2H, H-26, 28), 4.51 (1H, H-1'), 3.06 (1H, H-2'), 2.35 (1H, H-3'), 2.10 (6H, H-3'NMe$_2$), 1.59; 1.08 (2H, H-4'), 3.65 (1H, H-5'), 1.18 (3H, H-5'Me), 4.93 (1H, H-1''), 2.38; 1.57 (2H, H-2''), 1.24 (3H, H-3''Me), 3.21 (3H, H-3''OMe), 2.89 (1H, H-4''), 3.87; 3.74 (2H, H-4''a), 1.87 (2H, H-4''b), 3.37; 3.66 (2H, H-4''c), 7.54 (1H, H-4''d), 4.32 (1H, H-5''), 1.30 (3H, H-5''Me); purity: 98.1 %
4''_e (**38**)	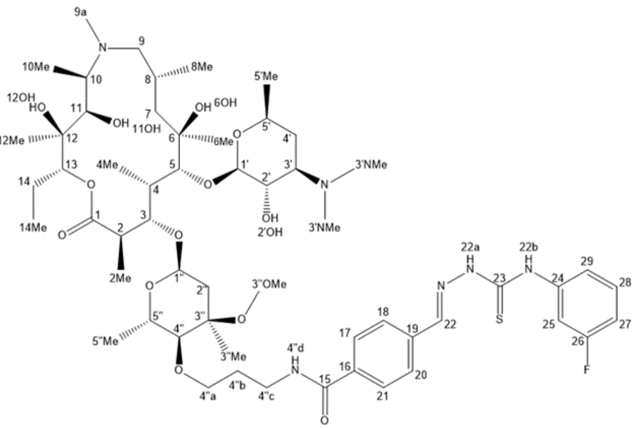	2.76 (1H, H-2), 1.17 (3H, H-2Me), 4.28 (1H, H-3), 1.97 (1H, H-4), 1.04 (3H, H-4Me), 3.61 (1H, H-5), 1.30 (3H, H-6Me), 1.65; 1.39 (2H, H-7), 2.00 (1H, H-8), 0.93 (3H, H-8Me), 2.53; 2.26 (2H, H-9), 2.34 (3H, H-9a), 2.83 (1H, H-10), 1.04 (3H, H-10Me), 3.60 (1H, H-11), 1.06 (3H, H-12Me), 4.74 (1H, H-13), 1.83; 1.46 (2H, H-14), 0.87 (3H, H-14Me), 7.91 (2H, H-17,21), 7.95 (2H, H-18,20), 8.10 (1H, H-22), 7.70 (1H, H-25), 7.01 (1H, H-27), 7.40 (1H, H-28), 7.49 (1H, H-29), 4.53 (1H, H-1'), 3.12 (1H, H-2'), 2.50 (1H, H-3'), 2.20 (6H, H-3'NMe$_2$), 1.66; 1.13 (2H, H-4'), 3.68 (1H, H-5'), 1.19 (3H, H-5'Me), 4.94 (1H, H-1''), 2.38; 1.57 (2H, H-2''), 1.25 (3H, H-3''Me), 3.24 (3H, H-3''OMe), 2.89 (1H, H-4''), 3.86; 3.74 (2H, H-4''a), 1.88 (2H, H-4''b), 3.39; 3.63 (2H, H-4''c), 7.54 (1H, H-4''d), 4.31 (1H, H-5''), 1.31 (3H, H-5''Me); purity: 97.9 %
4''_f (**39**)	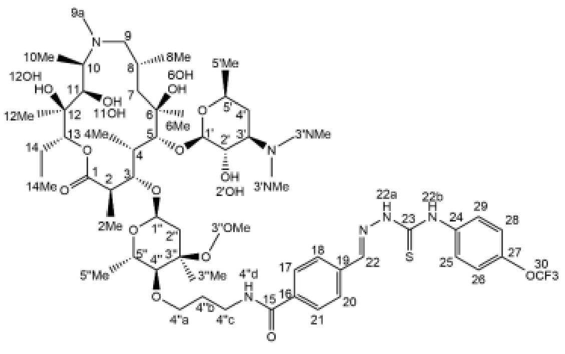	2.74 (1H, H-2), 1.18 (3H, H-2Me), 4.28 (1H, H-3), 1.97 (1H, H-4), 1.04 (3H, H-4Me), 3.61 (1H, H-5), 1.30 (3H, H-6Me), 1.67; 1.38 (2H, H-7), 1.99 (1H, H-8), 0.94 (3H, H-8Me), 2.52; 2.22 (2H, H-9), 2.33 (3H, H-9a), 2.80 (1H, H-10), 1.06 (3H, H-10Me), 3.56 (1H, H-11), 1.06 (3H, H-12Me), 4.75 (1H, H-13), 1.82; 1.47 (2H, H-14), 0.87 (3H, H-14 Me), 7.92 (2H, H17,21), 7.95 (2H, H-18,20), 8.12 (1H, H-22), 7.81 (2H, H-25, 29), 7.33 (2H, H-26, 28), 4.53 (1H, H-1'), 3.12 (1H, H-2'), 2.44 (1H, H-3'), 2.15 (6H, H-3'NMe$_2$), 1.63; 1.11 (2H, H-4'), 3.67(1H, H-5'), 1.19 (3H, H-5'Me), 4.93 (1H, H-1''), 2.37; 1.57 (2H, H-2''), 1.24 (3H, H-3''Me), 3.22 (3H, H-3''OMe), 2.89 (1H, H-4''), 3.86; 3.74 (2H, H-4''a), 1.87 (2H, H-4''b), 3.37; 3.68(2H, H-4''c), 4.31 (1H, H-5''), 1.31 (3H, H-5''Me); purity: 96.4 %

(cc) BY

Comp.	Structure	^{1}H NMR [δ / ppm]
4''_g (**40**)	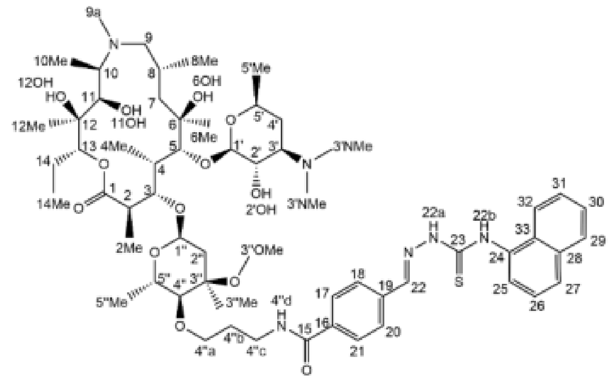	2.68 (1H, H-2), 1.15 (3H, H-2Me), 4.26 (1H, H-3), 1.87 (1H, H-4), 1.01 (3H, H-4Me), 3.55 (1H, H-5), 1.28 (3H, H-6Me), 1.29 (2H, H-7), 2.00 (1H, H-8), 0.93 (3H, H-8Me), 2.55; 2.33 (2H, H-9), 2.38 (3H, H-9a), 2.90 (1H, H-10), 1.13 (3H, H-10Me), 3.61 (1H, H-11), 1.10 (3H, H-12Me), 4.80 (1H, H-13), 1.88; 1.51 (2H, H-14), 0.90 (3H, H-14Me), 7.93 (2H, H-17,21), 7.97 (2H, H-18,20), 8.23 (1H, H-22), 7.98 (3H, H-25, 26, 27), 7.58 (4H, H-29, 30, 31, 32), 4.49 (1H, H-1'), 3.11 (1H, H-2'), 2.52 (1H, H-3'), 2.24 (6H, H-3' NMe_2), 1.67; 1.14 (2H, H-4'), 3.68 (1H, H-5'), 1.17 (3H, H-5'Me), 4.93 (1H, H-1''), 2.38; 1.56 (2H, H-2''), 1.25 (3H, H-3''Me), 3.28 (3H, H-3''OMe), 2.89 (1H, H-4''), 3.86; 3.74 (2H, H-4''a), 1.87 (2H, H-4''b), 3.43; 3.65 (2H, H-4''c), 7.58 (1H, H-4''d), 4.29 (1H, H-5''), 1.29 (3H, H-5''Me); purity: 95.4 %
4''_h (**41**)	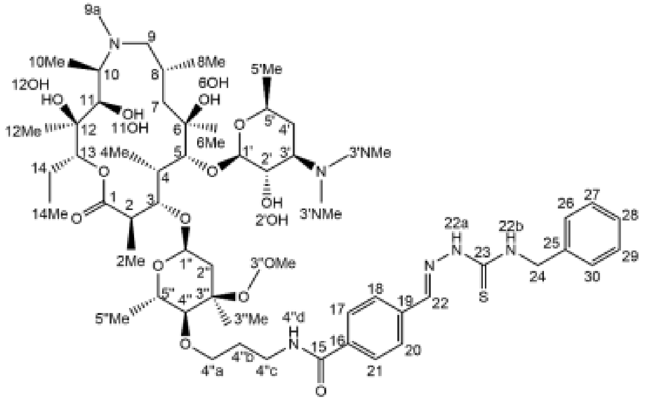	2.63 (1H, H-2), 1.09 (3H, H-2Me), 4.18 (1H, H-3), 1.82 (1H, H-4), 0.95 (3H, H-4Me), 3.39 (1H, H-5), 1.29 (3H, H-6Me), 1.71; 1.19 (2H, H-7), 1.96 (1H, H-8), 0.87 (3H, H-8Me), 2.56; 2.14 (2H, H-9), 2.34 (3H, H-9a), 2.78 (1H, H-10), 1.07 (3H, H-10Me), 3.58 (1H, H-11), 1.04 (3H, H-12Me), 4.67 (1H, H-13), 1.81; 1.41 (2H, H-14), 0.78 (3H, H-14Me), 8.05 (2H, H17,21), 7.80 (2H, H-18,20), 8.41 (1H, H-22), 4.89 (2H, H-24), 7.33 (2H, H-26, 30), 7.25 (2H, H-27,29), 7.18 (1H, H-28), 4.44 (1H, H-1'), 3.17 (1H, H-2'), 2.64 (1H, H-3'), 2.71 (6H, H-3' NMe_2), 1.61; 1.12 (2H, H-4'), 3.57 (1H, H-5'), 1.13 (3H, H-5'Me), 4.99 (1H, H-1''), 2.28; 1.44 (2H, H-2''), 1.23 (3H, H-3''Me), 3.20 (3H, H-3''OMe), 2.75 (1H, H-4''), 3.79; 3.65 (2H, H-4''a), 1.81 (2H, H-4''b), 3.33; 3.68 (2H, H-4''c), 4.23 (1H, H-5''), 1.29 (3H, H-5''Me); purity: 96.1 %
4''_i (**42**)	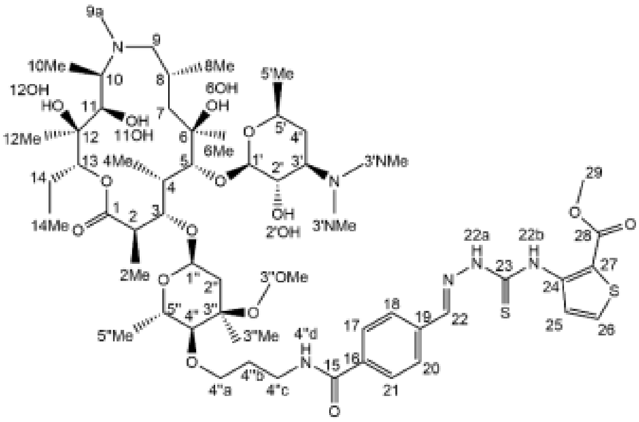	2.70 (1H, H-2), 1.17 (3H, H-2Me), 4.19 (1H, H-3), 1.95 (1H, H-4), 1.04 (3H, H-4Me), 3.59 (1H, H-5), 1.34 (3H, H-6Me), 1.72; 1.30 (2H, H-7), 2.05 (1H, H-8), 0.94 (3H, H-8Me), 2.65; 2.26 (2H, H-9), 2.48 (3H, H-9a), 2.87 (1H, H-10), 1.17 (3H, H-10Me), 3.75 (1H, H-11), 1.09 (3H, -12Me), 4.72 (1H, H-13), 1.84; 1.46 (2H, H-14), 0.88 (3H, H-14Me), 7.95 (2H, H17,21), 7.99 (2H, H-18,20), 8.06 (1H, H-22), 12.29 (1H, H-23a), 7.45 (1H, H-25), 8.97 (1H, H26), 3.93 (3H, H-29), 4.61 (1H, H-1'), 3.42 (1H, H-2'), 3.12 (1H, H-3'), 2.62 (6H, H-3'NMe_2), 1.84; 1.26 (2H, H-4'), 3.77 (1H, H-5'), 1.23 (3H, H-5'Me), 5.09 (1H, H-1''), 2.36; 1.53 (2H, H2''), 1.26 (3H, H-3''Me), 3.38 (3H, H-3''OMe), 2.83 (1H, H-4''), 3.71; 3.85 (2H, H-4''a), 1.91(2H, H-4''b), 3.53; 3.61 (2H, H-4''c), 7.76 (1H, H-4''d), 4.26 (1H, H-5''), 1.33 (3H, H-5''Me); purity: 97.3 %

Comp.	Structure	^{1}H NMR [δ / ppm]
4''_j (**43**)	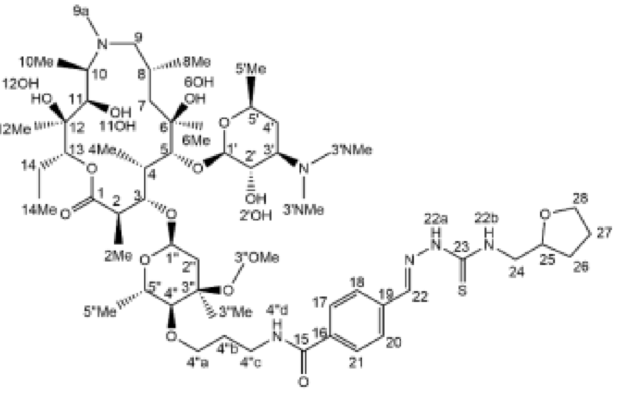	2.79 (1H, H-2), 1.19 (3H, H-2Me), 4.29 (1H, H-3), 2.01 (1H, H-4), 1.07 (3H, H-4Me), 3.59 (1H, H-5), 1.30 (3H, H-6Me), 1.63; 1.43 (2H, H-7), 2.03 (1H, H-8), 0.93 (3H, H-8Me), 2.32; 2.54 (2H, H-9), 2.37 (3H, H-9a), 2.87 (1H, H-10), 1.11 (3H, H-10Me), 3.61 (1H, H-11), 1.08 (3H, H-12Me), 4.81 (1H, H-13), 1.86; 1.47 (2H, H-14), 0.88 (3H, H-14Me), 7.97 (2H, H17,21), 7.90 (2H, H-18,20), 8.03 (1H, H-22), 8.09 (1H, H-22a), 3.62; 3.86 (2H, H-24), 4.15 (1H, H-25), 2.00; 1.65 (2H, H-26), 1.91 (2H, H-27), 3.88; 3.75 (2H, H-28), 4.53 (1H, H-1'), 3.14 (1H, H-2'), 2.55 (1H, H-3'), 2.23 (6H, H-3'NMe$_2$), 1.67; 1.14 (2H, H-4'), 3.69 (1H, H-5'), 1.19 (3H, H-5'Me), 4.95 (1H, H-1''), 2.36; 1.58 (2H, H-2''), 1.25 (3H, H-3''Me), 3.28 (3H, H-3''OMe), 2.89 (1H, H-4''), 3.86; 3.74 (2H, H-4''a), 1.87 (2H, H-4''b), 3.41; 3.61 (2H, H-4''c), 7.53 (1H, H-4''d), 4.32 (1H, H-5''), 1.31 (3H, H-5''Me); purity: 98.0 %
4''_k (**44**)	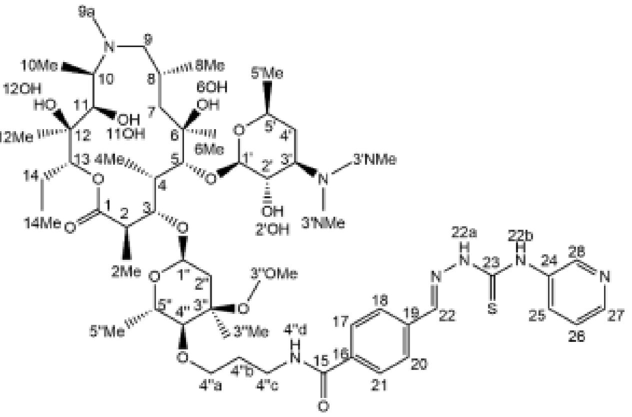	2.77 (1H, H-2), 1.17 (3H, H-2Me), 4.22 (1H, H-3), 2.01 (1H, H-4), 1.04 (3H, H-4 Me), 3.60 (1H, H-5), 1.32 (3H, H-6Me), 1.47; 1.63 (2H, H-7), 2.06 (1H, H-8), 0.96 (3H, H-8Me), 2.47; 2.66 (2H, H-9), 2.38 (3H, H-9a), 2.99 (1H, H-10), 1.16 (3H, H-10Me), 3.69 (1H, H-11), 1.09 (3H, H-12Me), 4.75 (1H, H-13), 1.84; 1.47 (2H, H-14), 0.88 (3H, H-14Me), 7.91 (2H, H17,21), 7.96 (2H, H-18,20), 8.12 (1H, H-22), 8.11 (1H, H-25), 7.39 (1H, H-26), 8.44 (1H, H27), 8.78 (1H, H 28), 4.56 (1H, H-1'), 3.20 (1H, H-2'), 2.77 (1H, H-3'), 2.35 (6H, H-3' NMe$_2$), 1.78; 1.21 (2H, H-4'), 3.75 (1H, H-5'), 1.21 (3H, H-5'Me), 4.94 (1H, H-1''), 2.38; 1.57 (2H, H2''), 1.24 (3H, H-3''Me), 3.30 (3H, H-3''OMe), 2.88 (1H, H 4''), 3.85; 3.74 (2H, H-4''a), 1.87 (2H, H-4''b), 3.43; 3.60 (2H, H-4''c), 4.28 (1H, H-5''), 1.32 (3H, H-5''Me); purity: 95.2 %
4''_l (**45**)	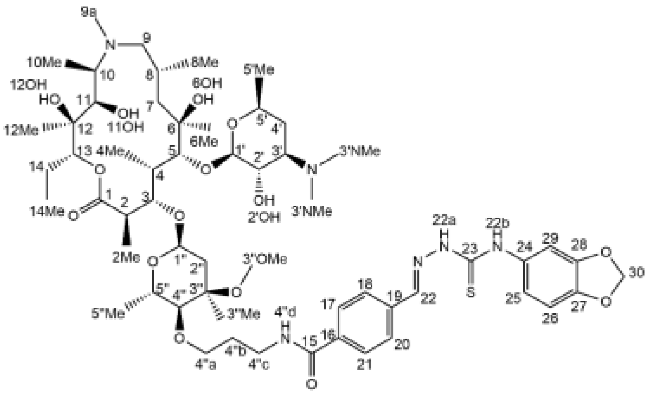	2.76 (1H, H-2), 1.17 (3H, H-2Me), 4.30 (1H, H-3), 1.96 (1H, H-4), 1.04 (3H, H-4Me), 3.61 (1H, H-5), 1.28 (3H, H-6Me), 1.36; 1.63 (2H, H-7), 1.97 (1H, H-8), 0.91 (3H, H-8Me), 2.16; 2.47 (2H, H-9), 2.29 (3H, H-9a), 2.76 (1H, H-10), 1.05 (3H, H-10Me), 3.57 (1H, H-11), 1.06 (3H, H-12Me), 4.75 (1H, H-13), 1.83; 1.47 (2H, H-14), 0.87 (3H, H-14Me), 7.90 (2H, H17,21), 7.93 (2H, H-18,20), 8.07 (1H, H-22), 7.22 (1H, H-25), 6.85 (1H, H-26), 6.98 (1H, H29), 6.02 (2H, H-30), 4.51 (1H, H-1'), 3.06 (1H, H-2'), 2.36 (1H, H-3'), 2.12 (6H, H-3'NMe$_2$), 1.58; 1.08 (2H, H-4'), 3.66 (1H, H-5'), 1.24 (3H, H-5'Me), 4.95 (1H, H-1''), 2.38; 1.57 (2H, H2''), 1.17 (3H, H-3''Me), 3.24 (3H, H-3''OMe), 2.88 (1H, H-4''), 3.85; 3.74 (2H, H-4''a), 1.87(2H, H-4''b), 3.39; 3.62 (2H, H-4''c), 4.32 (1H, H-5''), 1.30 (3H, H-5''Me); purity: 98.0 %

CC BY

Comp.	Structure	^{1}H NMR [δ / ppm]
4''_o (**46**)	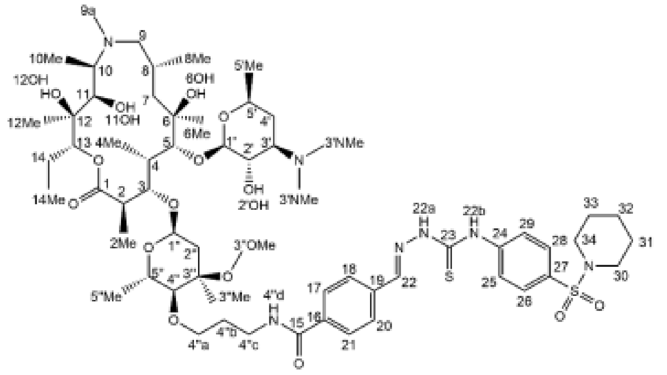	2.74 (1H, H-2), 1.16 (3H, H-2Me), 4.30 (1H, H-3), 1.95 (1H, H-4), 1.06 (3H, H-4 Me), 3.62 (1H, H-5), 1.31 (3H, H-6Me), 1.71; 1.49 (2H, H-7), 2.01 (1H, H-8), 0.95 (3H, H-8Me), 2.33; 2.52 (2H, H-9), 2.34 (3H, H-9a), 2.83 (1H, H-10), 1.07 (3H, H-10Me), 3.59 (1H, H-11), 1.02 (3H, H-12Me), 4.75 (1H, H-13), 1.82; 1.46 (2H, H-14), 0.87 (3H, H-14Me), 7.92 (2H, H17,21), 7.75 (2H, H-18,20), 8.14 (1H, H-22), 7.93 (2H, H-25,29), 8.12 (2H, H-26,28), 3.00 (2H, H-30,34), 1.63 (2H, H-31,33), 1.44 (1H, H-32), 4.55 (1H, H-1'), 3.13 (1H, H-2'), 2.46 (1H, H3'), 2.14 (6H, H-3'NMe$_2$), 1.64; 1.13 (2H, H-4'), 3.67 (1H, H-5'), 1.24 (3H, H-5'Me), 4.93 (1H, H 1"), 2.34; 1.57 (2H, H-2"), 1.20 (3H, H-3"Me), 3.19 (3H, H-3"OMe), 2.90 (1H, H-4"), 3.89; 3.76 (2H, H-4"a), 1.88 (2H, H-4"b), 3.36; 3.71 (2H, H-4"c), 7.67 (1H, H-4"d), 4.32 (1H, H5"), 1.31 (3H, H-5"Me); purity: 97.5 %
4''_1 (**47**)	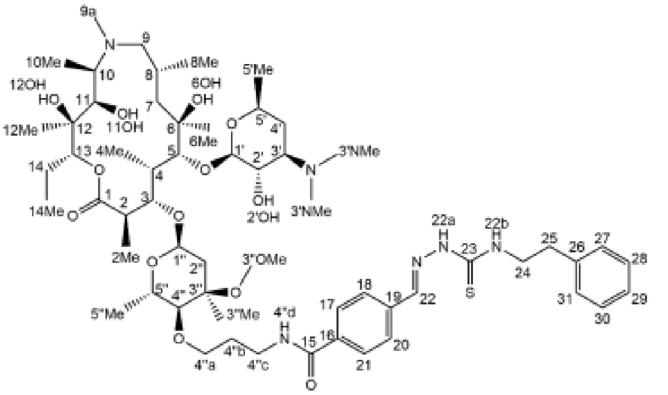	2.70 (1H, H-2), 1.17 (3H, H-2Me), 4.27 (1H, H-3), 1.95 (1H, H-4), 1.04 (3H, H-4Me), 3.59 (1H, H-5), 1.34 (3H, H-6Me), 1.72; 1.30 (2H, H-7), 2.05 (1H, H-8), 0.94 (3H, H-8Me), 2.65; 2.26 (2H, H-9), 2.48 (3H, H-9a), 2.85 (1H, H-10), 1.17 (3H, H-10Me), 3.60 (1H, H-11), 1.09(3H, H-12Me), 4.72 (1H, H-13), 1.84; 1.46 (2H, H-14), 0.88 (3H, H-14Me), 7.88 (2H, H17,21), 7.79 (2H, H-18,20), 7.98 (1H, H-22), 3.91 (2H, H-24), 3.01 (2H, H-25), 7.37 (2H, H27,31), 7.34 (2H, H-28,30), 7.28 (1H, H-29), 4.53 (1H, H-1'), 3.14 (1H, H-2'), 2.50 (1H, H-3'), 2.62 (6H, H-3'NMe$_2$), 1.67; 1.15 (2H, H-4'), 3.77 (1H, H-5'), 1.23 (3H, H-5'Me), 4.95 (1H, H1"), 2.36; 1.53 (2H, H-2"), 1.26 (3H, H-3"Me), 3.38 (3H, H-3"OMe), 2.83 (1H, H-4"), 3.71; 3.85 (2H, H-4"a), 1.91 (2H, H-4"b), 3.56 (2H, H-4"c), 7.76 (1H, H-4"d), 4.26 (1H, H-5"), 1.33(3H, H-5"Me); purity: 97.7 %
4''_9 (**48**)	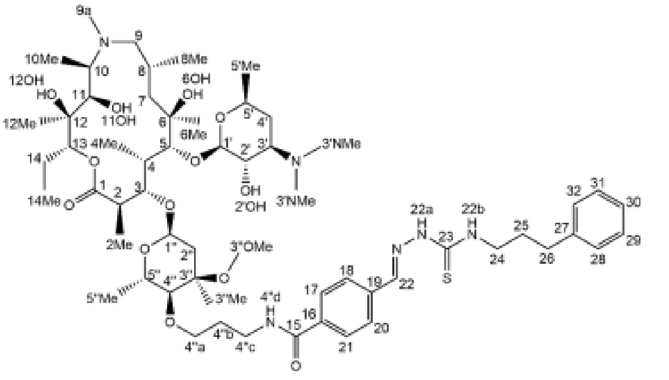	2.81 (1H, H-2), 1.20 (3H, H-2Me), 4.28 (1H, H-3), 2.00 (1H, H-4), 1.07 (3H, H-4Me), 3.61 (1H, H-5), 1.30 (3H, H-6Me), 1.43; 1.67 (2H, H-7), 2.01 (1H, H-8), 0.92 (3H, H-8Me), 2.25; 2.56 (2H, H-9), 2.38 (3H, H-9a), 3.00 (1H, H-10), 1.10 (3H, H-10Me), 3.56 (1H, H-11), 1.10 (3H, H-12Me), 4.78 (1H, H-13), 1.84; 1.49 (2H, H-14), 0.88 (3H, H-14Me), 7.88 (2H, H17,21), 7.86 (2H, H-18,20), 7.96 (1H, H-22), 3.71 (2H, 24), 2.01 (1H, H-25), 2.72 (1H, H-26),7.28 (2H, H-28,32), 7.31 (2H, H-29,31), 7.21 (1H, H-30), 4.53 (1H, H-1'), 3.20 (1H, H-2'), 2.77(1H, H-3'), 2.20 (6H, H-3'NMe$_2$), 1.66; 1.13 (2H, H-4'), 3.75 (1H, H-5'), 1.19 (3H, H-5'Me), 4.92 (1H, H-1"), 2.37; 1.57 (2H,H-2"), 1.25 (3H, H-3" Me), 3.25 (3H,H-3"OMe), 2.89 (1H, H-4"), 3.85; 3.74 (2H, H-4"a), 1.87 (2H, H-4"b), 3.39;3.62 (2H, H-4"c), 7.44 (1H, H-4"d),4.31 (1H, H-5"), 1.31 (3H, H-5"Me); purity: 96.5 %

Comp.	Structure	^{1}H NMR [δ / ppm]
4''_4abaR4 (**52**)	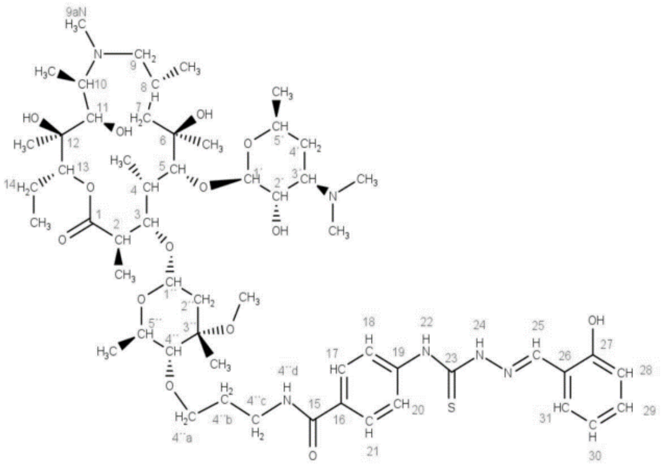	2.98 (1H, H-2), 0.95 (3H, H-2Me), 4.55 (1H, H-3), 1.37 (1H, H-4), 1.02 (3H, H-4Me), 4.75 (1H, H-5), 1.19 (3H, H-6Me), 2.98 (2H, H-7), 1.90 (1H, H-8), 0.87 (3H, H-8Me), 2.95 (2H, H-9), 2.35 (3H, H-9aNMe), 3.46 (1H, H-10), 1.14 (3H, H-10Me), 4.07 (1H, H-11), 0.99 (3H, H-12Me), 4.85 (1H, H-13), 1.51–2.27 (2H, H-14), 1.26 (3H, H-14), 4.62 (1H, H-1'), 3.09 (1H, H-2'), 2.35 (1H, H-3'), 2.84 (6H, H-3'NMe$_2$), 2.12 (2H, H-4'), 3.51 (1H, H-5'), 1.10 (3H, H-5'Me), 4.74 (1H, H-1''), 1.77–1.40 (2H, H-2''), 1.36 (3H, H-3''Me), 3.12 (3H, H-3''OMe), 3.43 (1H, H-4''), 4.07 (1H, H-5''), 1.14 (3H, H-5'' Me), 3.69 (2H, H-4''a), 1.57 (2H, H-4''b), 2.98 (2H, H-4''c), 6.75 (1H, H-4''d), 7.89 (1H, H-17), 7.69 (1H, H-18), 7.96 (1H, H-20), 7.92 (1H, H-21), 10.82 (1H, H-22), 10.26 (1H, H-24), 8.30 (1H, H-26), 8.10 (1H, H-29), 7.35 (1H, H-30), 7.15 (1H, H-31), 6.85 (1H, H-32); purity: 95.7 %
3-aminopropyl-AZI (**54**)	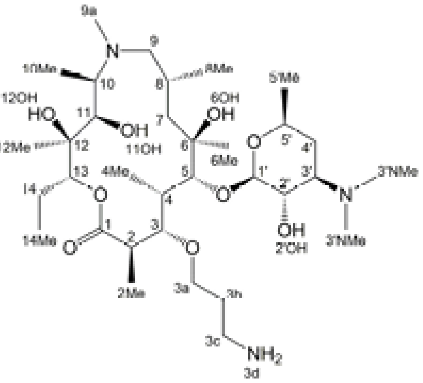	2.88 (1H, H-2), 1.24 (3H, H-2Me), 3.43 (1H, H-3), 3,75 (1H, H-3a), 1.78; 1.86 (2H, H-3b), 2.72 (1H, H-3c), 2.07 (1H, H-4), 0.90 (3H, H-4Me), 3.63 (1H, H-5), 1.19 (3H, H-6Me), 1.55; 1.30 (2H, H-7), 1.88 (1H, H-8), 0.85 (3H, H-8Me), 2.21; 2.48 (2H, H-9), 3.05; 2.26 (2H, H-9aN), 2.95 (1H, H-10), 1.02 (3H, H-10Me), 3.48 (1H, H-11), 1.05 (3H, H-12Me), 4.91 (1H, H13), 1.73; 1.52 (2H, H-14), 0.80 (3H, H-14Me), 4.40 (1H, H-1'), 3.32 (1H, H-2'), 2.70 (1H, H-3'), 2.23 (6H, H-3'NMe$_2$), 1.83; 1.30 (2H, H-4'), 3.52 (1H, H-5'), 1.19 (3H, H-5'Me); purity: 95.5 %
3_a (**55**)	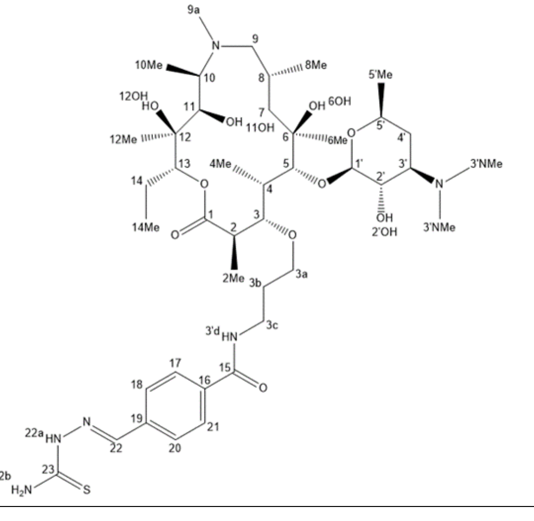	2.81 (1H, H-2), 1.28 (3H, H-2Me), 3.41 (1H, H-3), 2.14 (1H, H-4), 0.97 (3H, H-4Me), 3.63 (1H, H-5), 1.19 (3H, H-6Me), 1.54; 1.35 (2H, H-7), 1.87 (1H, H-8), 0.90 (3H, H-8Me), 2.43; 2.16 (2H, H-9), 2.31 (3H, H-9a), 2.80 (1H, H-10), 1.05 (3H, H-10Me), 3.57 (1H, H-11), 1.03 (3H, H-12Me), 4.77 (1H, H-13), 1.85; 1.51 (2H, H-14), 0.87 (3H, H-14Me), 7.84 (2H, H-17,21), 7.84 (2H, H-18,20), 8.02 (1H, H-22), 4.48 (1H, H-1'), 3.08 (1H, H-2'), 2.52 (1H, H-3'), 2.25 (6H, H-3'NMe$_2$), 1.67; 1.15 (2H, H-4'), 3.51 (1H, H-5'), 1.13 (3H, H-5'Me), 3.81 (2H, H-3a), 1.97 (2H, H-3b), 3.48 (2H, H-3c), 7.26 (1H,H-3d); purity: 96.1 %

(cc) BY

Comp.	Structure	^{1}H NMR [*δ* / ppm]
3_b (**56**)	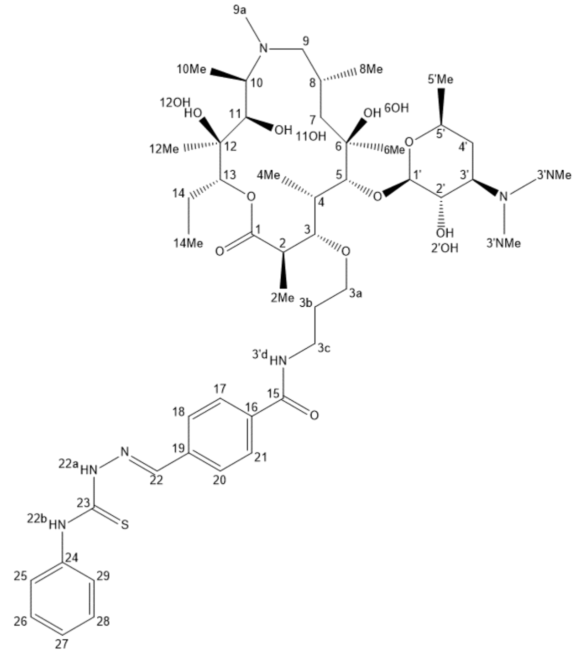	2.81 (1H, H-2), 1.28 (3H, H-2Me), 3.42 (1H, H-3), 2.14 (1H, H-4), 0.97 (3H, H-4Me), 3.63 (1H, H-5), 1.18 (3H, H-6Me), 1.53; 1.33 (2H, H-7), 1.87 (1H, H-8), 0.90 (3H, H-8Me), 2.43; 2.16 (2H, H-9), 2.31 (3H, H-9a), 2.81 (1H, H-10), 1.06 (3H, H-10Me), 3.57 (1H, H-11), 1.03 (3H, H-12Me), 4.77 (1H, H-13), 1.84; 1.50 (2H, H-14), 0.87 (3H, H-14Me), 7.86 (2H, H-17,21), 7.92 (2H, H-18,20), 8.08 (1H, H-22), 9.50 (1H, H-22b), 7.43 (2H, H-26,28) 7.64 (2H, H-25,29), 7.27 (1H, H-27) 4.48 (1H, H-1'), 3.09 (1H, H-2'), 2.52 (1H, H-3'), 2.26 (6H, H-3'NMe_2), 1.66; 1.14 (2H, H-4'), 3.52 (1H, H-5'), 1.14 (3H, H-5'Me), 3.80 (2H, H-3a), 1.97 (2H, H-3b), 3.48 (2H, H-3c), 7.27 (1H, H-3d); purity: 97.0 %
3_c (**57**)	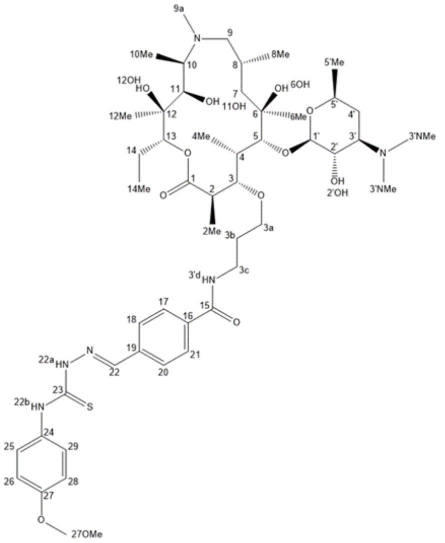	2.81 (1H, H-2), 1.28 (3H, H-2Me), 3.41 (1H, H-3), 2.14 (1H, H-4), 0.97 (3H, H-4Me), 3.63 (1H, H-5), 1.19 (3H, H-6Me), 1.52; 1.33 (2H, H-7), 1.87 (1H, H-8), 0.90 (3H, H-8Me), 2.43; 2.15 (2H, H-9), 2.31 (3H, H-9a), 2.81 (1H, H-10), 1.05 (3H, H-10Me), 3.56 (1H, H-11), 1.03 (3H, H-12Me), 4.77 (1H, H-13), 1.84; 1.50 (2H, H-14), 0.87 (3H, H-14Me), 7.85 (2H, H-17,21), 7.91 (2H, H-18,20), 8.06 (1H, H-22), 7.46 (2H, H-25,29), 6.96 (1H, H-26,28), 3.83 (3H, H-27OMe), 4.48 (1H, H-1'), 3.07 (1H, H-2'), 2.51 (1H, H-3'), 2.26 (6H, H-3'NMe_2), 1.67; 1.14 (2H, H-4'), 3.51 (1H, H-5'), 1.15 (3H, H-5'Me), 3.80 (2H, H-3a), 1.97 (2H, H-3b), 3.48 (2H, H-3c), 7.24 (1H, H-3d); purity: 98.5 %
3_d (**58**)	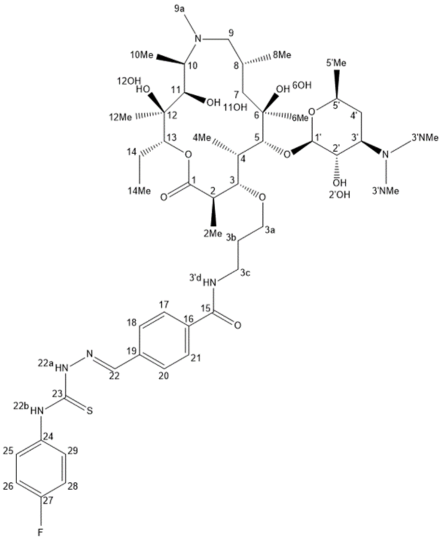	2.81 (1H, H-2), 1.28 (3H, H-2Me), 3.41 (1H, H-3), 2.14 (1H, H-4), 0.97 (3H, H-4Me), 3.63 (1H, H-5), 1.19 (3H, H-6Me), 1.53; 1.33 (2H, H-7), 1.88 (1H, H-8), 0.89 (3H, H-8Me), 2.44; 2.16 (2H, H-9), 2.31 (3H, H-9a), 2.81 (1H, H-10), 1.06 (3H, H-10Me), 3.56 (1H, H-11), 1.02 (3H, H-12Me), 4.77 (1H, H-13), 1.84; 1.50 (2H, H-14), 0.87 (3H, H-14Me), 7.86 (2H, H-17,21), 7.92 (2H, H-18,20), 8.07 (1H, H-22), 7.61 (2H, H-25,29), 7.16 (2H, H-26,28), 4.46 (1H, H-1'), 3.08 (1H, H-2'), 2.52 (1H, H-3'), 2.27 (6H, H-3'NMe_2), 1.66; 1.14 (2H, H-4'), 3.51 (1H, H-5'), 1.14 (3H, H-5'Me), 3.80 (2H, H-3a), 1.97 (2H, H-3b), 3.47 (2H, H-3c), 7.26 (1H, H-3d); purity: 97.0 %

Comp.	Structure	^{1}H NMR [δ / ppm]
3_e (**59**)	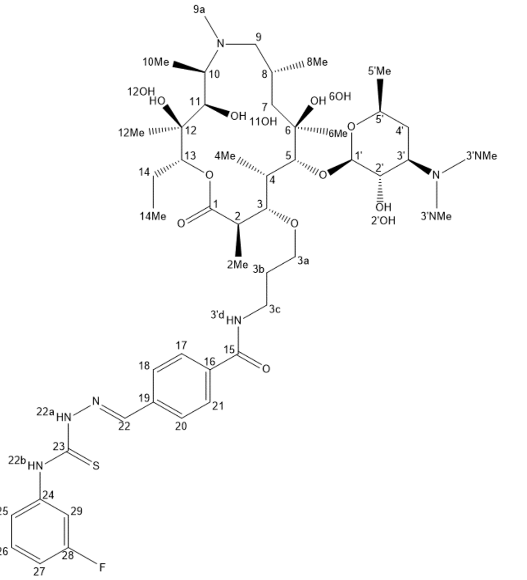	2.81 (1H, H-2), 1.28 (3H, H-2Me), 3.41 (1H, H-3), 2.13 (1H, H-4), 0.97 (3H, H-4Me), 3.64 (1H, H-5), 1.19 (3H, H-6Me), 1.52; 1.32 (2H, H-7), 1.87 (1H, H-8), 0.90 (3H, H-8Me), 2.43; 2.18 (2H, H-9), 2.31 (3H, H-9a), 2.80 (1H, H-10), 1.06 (3H, H-10Me), 3.56 (1H, H-11), 1.03 (3H, H-12Me), 4.77 (1H, H-13), 1.87; 1.51 (2H, H-14), 0.87 (3H, H-14Me), 7.87 (2H, H-17,21), 7.93 (2H, H-18,20), 8.09 (1H, H-22), 7.45 (1H, H-25), 7.42 (1H, H-26), 7.02 (1H, H-27), 7.66 (1H, H-29), 4.48 (1H, H-1'), 3.09 (1H, H-2'), 2.52 (1H, H-3'), 2.27 (6H, H-3'NMe_2), 1.67; 1.15 (2H, H-4'), 3.51 (1H, H-5'), 1.15 (3H, H-5'Me), 3.81 (2H, H-3a), 1.97 (2H, H-3b), 3.49 (2H, H-3c), 7.26 (1H,H-3d); purity: 96.8 %
3_5 (**60**)	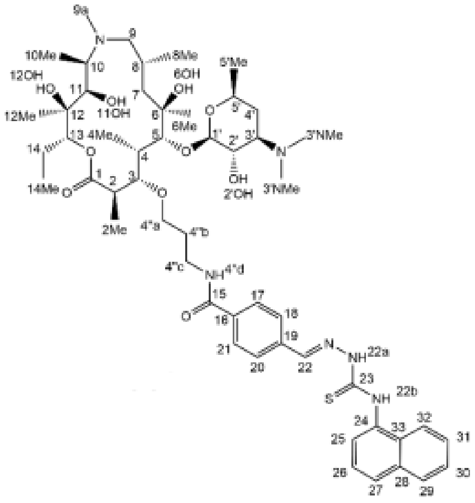	2.81 (1H, H-2), 1.28 (3H, H-2Me), 3.41 (1H, H-3), 2.15 (1H, H-4), 0.97 (3H, H-4Me), 3.64 (1H, H-5), 1.18 (3H, H-6Me), 1.51; 1.32 (2H, H-7), 1.90 (1H, H-8), 0.90 (3H, H-8Me), 2.42; 2.14 (2H, H-9), 2.30 (3H, H-9a), 2.81 (1H, H-10), 1.05 (3H, H-10Me), 3.55 (1H, H-11), 1.02 (3H, H-12Me), 4.76 (1H, H-13), 1.83; 1.51 (2H, H-14), 0.87 (3H, H-14Me), 7.86 (2H, H-17,21), 7.94 (2H, H-18,20), 8.15 (1H, H 22), 7.98 (3H, H-25, 26, 27), 7.58 (4H, H-29,30,31,32), 4.48 (1H, H-1'), 3.07 (1H, H-2'), 2.53 (1H, H-3'), 2.24 (6H, H 3'NMe_2), 1.65; 1.15 (2H, H-4'), 3,49 (1H, H-5'), 1.13 (3H, H-5'Me), 3.81 (2H, H-3a), 1.96 (2H, H-3b), 3.47 (2H, H-3c), 7.43 (2H,H-3d); purity: 95.3 %
3_16 (**61**)	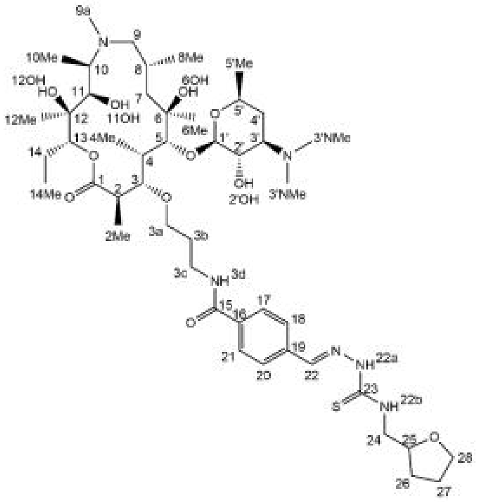	2.78 (1H, H-2), 1.25 (3H, H-2Me), 3.39 (1H, H-3), 2.10 (1H, H-4), 0.94 (3H, H-4Me), 3.59 (1H, H-5), 1.15 (3H, H-6Me), 1.51; 1.30 (2H, H-7), 1.84 (1H, H-8), 0.86 (3H, H-8Me), 2.40; 2.12 (2H, H-9), 2.28 (3H, H-9a), 2.76 (1H, H-10), 1.02 (3H, H-10Me), 3.53 (1H, H-11), 1.00 (3H, H-12Me), 4.75 (1H, H-13), 1.81; 1.47 (2H, H-14), 0.83 (3H, H-14Me), 7.82 (2H, H17,21), 7.78 (2H, H-18,20), 7.96 (1H, H-22), 7.97 (1H, H-22b), 3.63; 3.81 (2H, H-24), 4.13 (1H, H-25), 1.97; 1.63 (2H, H-26), 1.88 (2H, H-27), 3.85; 3.71 (2H, H-28), 4.43 (1H, H-1'), 3.05 (1H, H-2'), 2.48 (1H, H-3'), 2.22 (6H, H 3'NMe_2), 1.62; 1.11 (2H, H-4'), 3.47 (1H, H-5'), 1.09 (3H, H-5'Me), 3.77 (2H, H-3a), 1.94 (2H, H-3b), 3.44 (2H, H-3c), 7.29 (2H, H-3d); purity: 95.7 %

CC BY

Comp.	Structure	^{1}H NMR [δ / ppm]
3_10 (**62**)	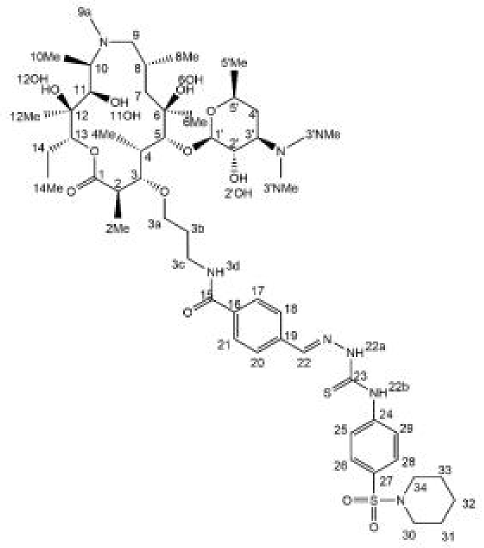	2.77 (1H, H-2), 1.25 (3H, H-2Me), 3.41 (1H, H-3), 2.13 (1H, H-4), 0.95 (3H, H-4Me), 3.63 (1H, H-5), 1.18 (3H, H-6Me), 1.53; 1.34 (2H, H-7), 1.84 (1H, H-8), 0.89 (3H, H-8Me), 2.42; 2.16 (2H, H-9), 2.31 (3H, H-9a), 2.80 (1H, H-10), 1.05 (3H, H-10Me), 3.56 (1H, H-11), 0.99 (3H, H-12Me), 4.81 (1H, H-13), 1.82; 1.47 (2H, H-14), 0.86 (3H, H-14Me), 7.87 (2H, H17,21), 7.94 (2H, H-18,20), 8.11 (1H, H-22), 7.95 (2H, H-25,29), 8.13 (2H, H-26,28), 3.01 (2H, H-30,34), 1.60 (2H, H-31,33), 1.42 (1H, H-32), 4.42 (1H, H-1'), 3.08 (1H, H-2'), 2.47 (1H, H3'), 2.25 (6H, H-3'NMe_2), 1.63; 1.14 (2H, H-4'), 3.47 (1H, H-5'), 1.14 (3H, H-5'Me), 3.81 (2H, H-3a), 1.98 (2H, H-3b), 3.50 (2H, H-3c); purity: 96.4 %
3_1 (**63**)	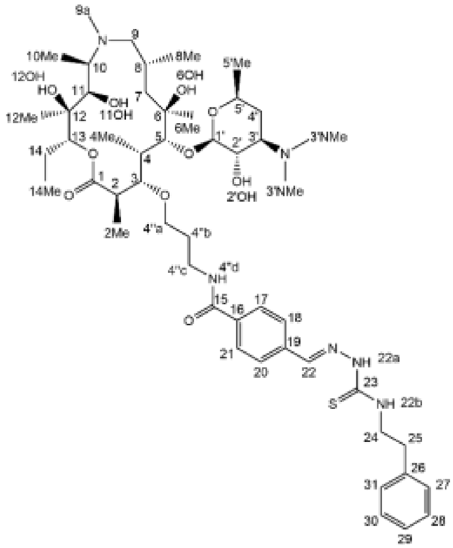	2.83 (1H, H-2), 1.30 (3H, H-2Me), 3.46 (1H, H-3), 2.10 (1H, H-4), 0.94 (3H, H-4Me), 3.64 (1H, H-5), 1.23 (3H, H-6Me), 1.57; 1.38 (2H, H-7), 1.94 (1H, H-8), 0.91 (3H, H-8Me), 2.50; 2.17 (2H, H-9), 2.43 (3H, H-9a), 2.85 (1H, H-10), 1.08 (3H, H-10Me), 3.56 (1H, H-11), 1.05 (3H, H-12Me), 4.79 (1H, H-13), 1.85; 1.52 (2H, H-14), 0.89 (3H, H-14Me), 7.85 (2H, H17,21), 7.77 (2H, H-18,20), 7.96 (1H, H-22), 3.91 (2H, H-24), 3.01 (2H, H-25), 7.37 (2H, H27,31), 7.34 (2H, H-28,30), 7.28 (1H, H-29), 4.48 (1H, H 1'), 3.17 (1H, H-2'), 2.76 (1H, H-3'), 2.39 (6H, H-3'NMe_2), 1.78; 1.26 (2H, H-4'), 3.57 (1H, H-5'), 1.18 (3H, H-5'Me), 3.80 (2H, H3a), 1.98 (2H, H-3b), 3.48 (2H, H 3c), 7.43 (2H, H-3d); purity: 95.5 %
3_9 (**64**)	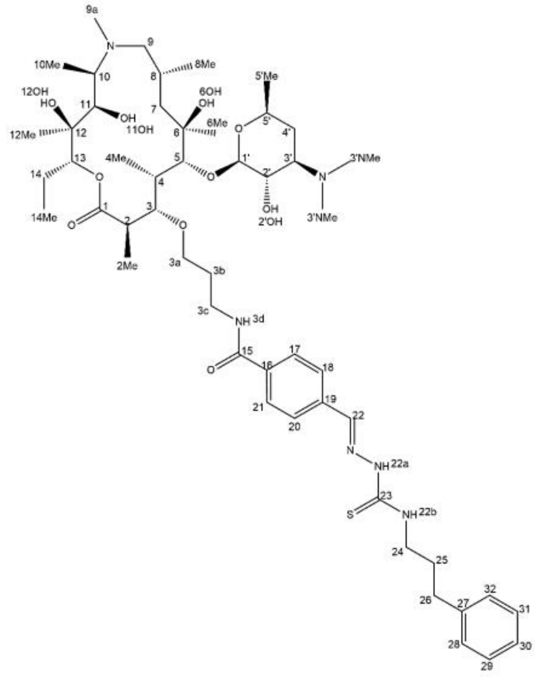	2.77 (1H, H-2), 1.25 (3H, H-2Me), 3.38 (1H, H-3), 2.11 (1H, H-4), 0.94 (3H, H-4Me), 3.59 (1H, H-5), 1.15 (3H, H-6Me), 1.50; 1.30 (2H, H-7), 1.84 (1H, H-8), 0.87 (3H, H-8Me), 2.40; 2.12 (2H, H-9), 2.27 (3H, H-9a), 2.77 (1H, H-10), 1.02 (3H, H-10Me), 3.53 (1H, H-11), 0.99 (3H, H-12Me), 2.91 (1H, H-12O), 4.74 (1H, H-13), 1.81; 1.47 (2H, H-14), 0.83 (3H, H-14Me), 7.81 (1H, H-17; H-21), 7.81 (1H, H-18; H-20), 7.93 (1H, H-22), 7.92 (1H, H-4'' 22b), 3.66 (2H, H-24), 1.98 (2H, H-25), 1.97; 2.68 (2H, H-26), 7.25 (1H, H-28; H-32), 7.28 (1H, H-29; H-31), 4.44 (1H, H-1'), 3.05 (1H, H-2'), 2.47 (1H, H-3'), 2.22 (6H, H-3'NMe_2), 1.63; 1.11 (2H, H-4'), 3.47 (1H, H-5'), 1.10 (3H, H-5'Me), 3.77 (2H, H-3a), 1.94 (2H, H-3b), 3.44 (2H, H-3c), 7.24 (1H, H-3d); purity: 95.6 %

Comp.	Structure	^{1}H NMR [δ / ppm]
3_6 (**65**)	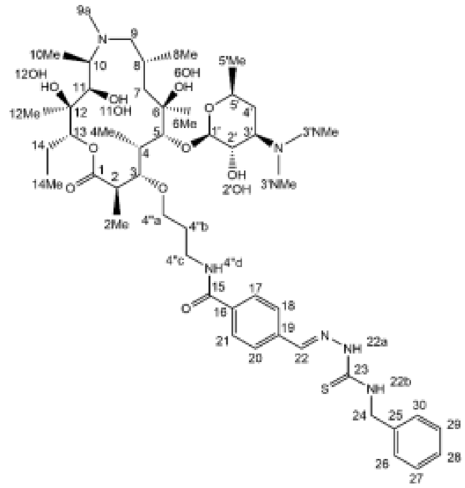	2.83 (1H, H-2), 1.30 (3H, H-2Me), 3.46 (1H, H-3), 2.10 (1H, H-4), 0.94 (3H, H-4Me), 3.64 (1H, H-5), 1.23 (3H, H-6Me), 1.57; 1.38 (2H, H-7), 1.94 (1H, H-8), 0.91 (3H, H-8Me), 2.50; 2.17 (2H, H-9), 2.30 (3H, H-9a), 2.80 (1H, H-10), 1.05 (3H, H-10Me), 3.56 (1H, H-11), 1.05 (3H, H-12Me), 4.79 (1H, H-13), 1.85; 1.52 (2H, H-14), 0.89 (3H, H-14Me), 7.85 (2H, H17,21), 7.77 (2H, H-18,20), 7.96 (1H, H-22), 4.93 (2H, H-24), 3.01 (2H, H-25), 7.37 (2H, H27,31), 7.34 (2H, H-28,30), 7.28 (1H, H-29), 4.48 (1H, H 1'), 3.17 (1H, H-2'), 2.51 (1H, H-3'), 2.24 (6H, H-3'NMe$_2$), 1.66; 1.14 (2H, H-4'), 3.57 (1H, H-5'), 1.18 (3H, H-5'Me), 3.80 (2H, H3a), 1.98 (2H, H-3b), 3.48 (2H, H 3c), 7.43 (2H, H-3d); purity: 95.9 %
3_8 (**66**)	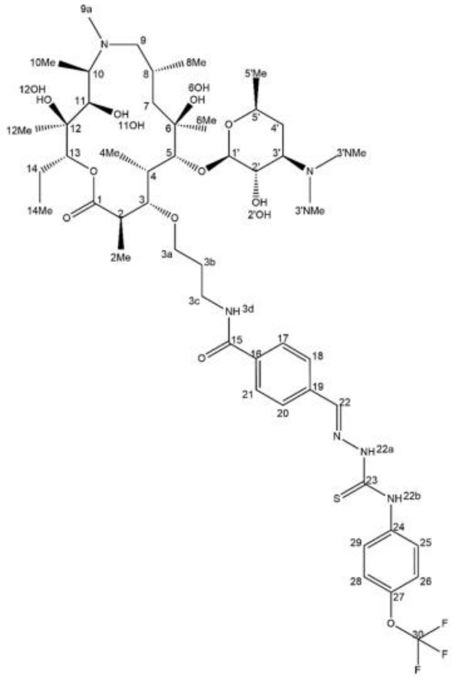	2.77 (1H, H-2), 1.24 (3H, H-2Me), 3.39 (1H, H-3), 2.07 (1H, H-4), 0.92 (3H, H-4Me), 3.59 (1H, H-5), 1.16 (3H, H-6Me), 1.53; 1.29 (2H, H-7), 1.84 (1H, H-8), 0.85 (3H, H-8Me), 2.40; 2.12 (2H, H-9), 2.26 (3H, H-9a), 2.76 (1H, H-10), 1.01 (3H, H-10Me), 3.51 (1H, H-11), 0.99 (3H, H-12.Me), 4.79 (1H, H-13), 1.82; 1.46 (2H, H-14), 0.81 (3H, H-14Me), 7.83 (1H, H-17; H-21), 7.90 (1H, H-18; H-20), 8.08 (1H, H-22), 7.69 (1H, H-25; H-29), 7.30 (1H, H-26; H-28), 4.42 (1H, H-1'), 3.09 (1H, H-2'), 2.52 (1H, H-3'), 2.22 (6H, H-3'NMe$_2$), 1.62; 1.12 (2H, H-4'), 3.46 (1H, H-5'), 1.08 (3H, H-5'Me), 3.76 (2H, H-3a), 1.94 (2H, H-3b), 3.44 (2H, H-3c), 7.57 (1H, H-3d); purity: 95.1 %
3_12 (**67**)	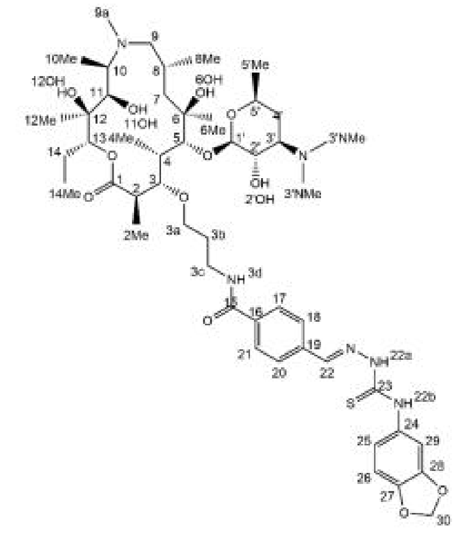	2.77 (1H, H-2), 1.25 (3H, H-2Me), 3.38 (1H, H-3), 2.11 (1H, H-4), 0.94 (3H, H-4Me), 3.59 (1H, H-5), 1.19 (3H, H-6Me), 1.54; 1.33 (2H, H-7), 1.84 (1H, H-8), 0.87 (3H, H-8Me), 2.44; 2.16 (2H, H-9), 2.31 (3H, H-9a), 2.80 (1H, H-10), 1.05 (3H, H-10Me), 3.57 (1H, H-11), 0.99 (3H, H-12Me), 4.74 (1H, H-13), 1.82; 1.47 (2H, H-14), 0.87 (3H, H-14Me), 7.86 (2H, H17,21), 7.90 (2H, H-18,20), 8.07 (1H, H-22), 7.22 (1H, H-25), 6.85 (1H, H-28), 6.98 (1H, H29), 6.02 (2H, H-30), 4.44 (1H, H-1'), 3.08 (1H, H-2'), 2.47 (1H, H-3'), 2.25 (6H, H-3'NMe$_2$), 1.63; 1.14 (2H, H-4'), 3.47 (1H, H-5'), 1.13 (3H, H-5'Me), 3.80 (2H, H-3a), 1.94 (2H, H-3b), 3.47 (2H, H-3c), 7.24 (2H, H-3d); purity: 96.3 %

CC BY

3_7 (**68**)	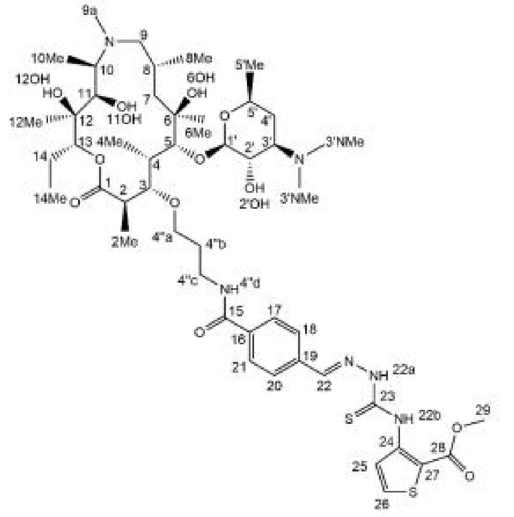	2.77 (1H, H-2), 1.25 (3H, H-2Me), 3.41 (1H, H-3), 2.13 (1H, H-4), 0.98 (3H, H-4Me), 3.63 (1H, H-5), 1.18 (3H, H-6Me), 1.53; 1.34 (2H, H-7), 1.84 (1H, H-8), 0.90 (3H, H-8Me), 2.42;2.16 (2H, H-9), 2.31 (3H, H-9a), 2.80 (1H, H-10), 1.05 (3H, H-10Me), 3.56 (1H, H-11), 0.99 (3H, H-12Me), 4.79 (1H, H-13), 1.82; 1.47 (2H, H-14), 0.86 (3H, H-14Me), 7.90 (2H, H17,21), 8.05 (2H, H-18,20), 8.11 (1H, H-22), 12.29 (1H, H-23a), 8.98 (1H, H-25), 7.70 (1H, H26), 3.93 (3H, H-29), 4.44 (1H, H-1'), 3.08 (1H, H-2'), 2.47 (1H, H-3'), 2.25 (6H, H-3'NMe$_2$), 1.63; 1.14 (2H, H-4'), 3.47 (1H, H-5'), 1.14 (3H, H-5'Me), 3.81 (2H, H-3a), 1.98 (2H, H-3b), 3.50 (2H, H-3c), 7.24 (2H, H-3d); purity: 96.8 %
